# Integrative Genomic Analyses Identify and Prioritize Candidate Loci for Metatarsal Length and Circumference in Broilers

**DOI:** 10.3390/ani16203189

**Published:** 2026-10-11

**Authors:** Hengcong Chen, Xinquan Li, Xin Fan, Cheng Chang, Jiamei Zhou, Desheng Wang, Shengwei Yang, Qihan Yang, Zhiping Cao, Peng Luan, Yumao Li, Hui Zhang

**Affiliations:** College of Animal Science and Technology, Northeast Agricultural University, Harbin 150030, China; hengcongchen@neau.edu.cn (H.C.); lixinquan2001@163.com (X.L.); 15938875013@163.com (X.F.); b230501003@neau.edu.cn (C.C.); zhoujiamei@neau.edu.cn (J.Z.); w19135816468@163.com (D.W.); yangshengwei@neau.edu.cn (S.Y.); yqh1354@outlook.com (Q.Y.); caozhiping@neau.edu.cn (Z.C.); luan0901@neau.edu.cn (P.L.); liyumao@neau.edu.cn (Y.L.)

**Keywords:** broiler chicken, skeletal morphology, whole-genome resequencing, bivariate association analysis, linkage disequilibrium, genomic prediction

## Abstract

The objective of this study was to characterize the genetic basis of metatarsal length (MeL) and circumference (MeC), identify candidate loci and genes associated with these traits, and evaluate the predictive utility of integrated candidate-marker sets for broiler leg traits. A total of 330 male broilers phenotyped at 49 days of age and subjected to whole-genome resequencing were used for the analyses. Genomic heritability estimates were 0.266 for MeL and 0.516 for MeC. Single-trait GWAS identified 102 and 165 suggestive SNPs for MeL and MeC, respectively, while bivariate analysis identified 263 SNPs, including 159 not detected by either single-trait analysis. Integration with genetic differentiation and nucleotide diversity prioritized 55 candidate SNPs for MeL and 62 for MeC, with three loci shared between the two traits. Linkage disequilibrium refinement and functional annotation identified 120 candidate variants and 64 candidate genes, including *APP*, *LDB3*, *ADH6*, *MTTP*, and a missense variant in *ANKRD50*. Across six models, mean heritability-adjusted prediction estimates ranged from 0.197 to 0.230 for MeL and 0.273 to 0.322 for MeC. Exploratory within-population analyses indicated that the candidate-marker sets were enriched for trait-relevant predictive information. These results provide insights into the genetic architecture of broiler leg traits and provide candidate markers for further validation and evaluation in genomic prediction.

## Simple Summary

Fast-growing broilers may experience leg problems when rapid body weight gain exceeds skeletal development, potentially affecting movement, welfare, and production. We measured the length and circumference of the metatarsal bone in 330 male broilers and analyzed whole-genome data to identify genetic regions associated with these traits. Integrating multiple complementary analyses identified 55 candidate markers for metatarsal length and 62 for circumference, including three markers shared by both traits. We also identified 64 candidate genes, including genes that may be related to bone, muscle, and energy metabolism. In an exploratory analysis within the same population, the selected markers showed greater trait-related predictive information than matched random marker sets. These findings improve understanding of the genetic basis of broiler leg development and provide candidate markers for future validation, which may support future breeding efforts focused on improving broiler leg traits.

## 1. Introduction

Chicken meat is a major source of animal protein, and sustained selection has markedly increased broiler growth rate and breast-muscle yield. However, skeletal development may not keep pace with rapid body-weight gain, increasing the risk of leg disorders, impaired locomotion, production losses, and compromised welfare [1,2,3,4]. Improving leg development while maintaining growth performance is therefore an important breeding objective.

Metatarsal length (MeL) and metatarsal circumference (MeC) describe longitudinal and transverse shank development, respectively, and provide practical indicators of leg morphology and skeletal support. Both traits are complex and are influenced by genetic and environmental factors [3,4,5,6]. Previous genome-wide association studies (GWASs) have identified loci associated with chicken skeletal growth and metatarsal development [5,6], but single-trait analyses may not fully exploit information shared between correlated traits. Bivariate GWAS can model trait covariance and may identify shared or weak association signals that are missed in separate analyses [7].

GWAS-associated loci often represent statistical associations rather than causal variants and may encompass broad linkage-disequilibrium (LD) regions. Therefore, complementary genomic evidence is important for prioritizing biologically relevant candidate variants. Differences in allele frequency and nucleotide diversity between individuals with divergent phenotypes can provide complementary evidence for identifying trait-associated regions [6,8]. Subsequent LD refinement and functional annotation can further narrow associated signals and facilitate the identification of candidate variants and genes underlying complex traits.

Genomic prediction provides an important bridge between genetic discovery and practical breeding applications. Models such as genomic best linear unbiased prediction (GBLUP), BayesA, BayesB, BayesCπ, Bayesian LASSO, and reproducing kernel Hilbert space (RKHS) regression differ in their assumptions about marker effects and genetic architecture [9,10,11,12,13,14]. Prediction can also depend on marker composition: genome-wide sets capture polygenic background, whereas candidate-marker sets may concentrate trait-relevant information. Evaluating the predictive performance of candidate markers therefore provides a means to assess their potential value for genomic selection [15].

This study investigated the genetic architecture of MeL and MeC in 330 male broilers using single-trait and bivariate GWAS, genetic differentiation and nucleotide diversity analyses of extreme phenotypic groups, LD-based signal refinement, and functional annotation. We further compared six genomic prediction models using a common representative SNP set and conducted an exploratory within-population assessment of candidate-marker sets against full-density and matched-random marker sets. Compared with previous studies based primarily on single-trait GWAS or selection-signature analyses, the present study integrates single-trait and bivariate GWAS with phenotype-based genetic differentiation and nucleotide diversity analyses, followed by LD-based refinement and functional annotation, to prioritize candidate loci supported by multiple complementary lines of genomic evidence. The objectives were to characterize the genetic basis of metatarsal morphology, identify candidate loci and genes, and assess the within-population informativeness of the integrated candidate-marker sets.

## 2. Materials and Methods

### 2.1. Experimental Animals

A total of 330 male chickens from generation 19 (G19) of the Northeast Agricultural University broiler lines divergently selected for abdominal fat content were used, including 160 birds from the high-fat line and 170 from the low-fat line [16]. All birds were reared in the same experimental facility under standardized conditions, with a nutritionally complete diet and water provided ad libitum.

All birds were slaughtered at 49 days of age after 12 h of feed withdrawal, while water remained available. Live body weight (BW) was recorded before slaughter. Metatarsal length (MeL) of the left leg was measured as the straight-line distance between the proximal and distal ends of the tarsometatarsus using a vernier caliper and was recorded in millimeters (mm). Metatarsal circumference (MeC) was measured at the junction of the middle and distal thirds of the tarsometatarsus using a flexible tape held horizontally and without constriction. The tape was wrapped twice completely around the shank; the recorded two-turn value was divided by 2 to obtain the circumference of one complete turn, which was used in all analyses and reported in centimeters (cm). Measurements were conducted with reference to the Agricultural Industry Standard of the People’s Republic of China, NY/T 823-2020 [17], Poultry Performance Terms and Measurement Methods, and were performed by one operator. Animal procedures were approved by the Laboratory Animal Ethics Committee of Northeast Agricultural University (approval no. NEAUEC20220228).

### 2.2. Methods

#### 2.2.1. Phenotypic and Genotypic Data Processing and Heritability Estimation

Descriptive statistical analyses of MeL and MeC were performed using R. The sample size, mean, standard deviation, standard error, median, minimum, maximum, and coefficient of variation were calculated. The coefficient of variation was calculated as the ratio of the standard deviation to the mean. All individuals with complete phenotypic records were retained, and no individuals were excluded on the basis of extreme phenotypic values.

Blood samples were collected from the wing vein of all experimental chickens 2 days before slaughter using EDTA as the anticoagulant. Genomic DNA was extracted from whole blood and subjected to whole-genome resequencing at an average sequencing depth of approximately 10×. After quality control, the sequencing reads were aligned to the *Gallus gallus* GRCg7b reference genome. Variant calling was subsequently performed, and only biallelic single-nucleotide polymorphisms (SNPs) were retained, yielding an initial genotype dataset containing 13,042,622 SNPs.

Genotype quality control was performed using PLINK v1.90b6.21 [18]. Genotype and phenotype identifiers were first matched, and only individuals with complete records for MeL, MeC, and BW were retained. Individuals with genotype missingness greater than 5% were excluded using --mind 0.05, and SNPs with missingness greater than 5% were removed using --geno 0.05. SNPs with a minor allele frequency lower than 5% were excluded using --maf 0.05. Considering the inheritance characteristics of avian sex chromosomes, SNPs on the W chromosome were excluded, whereas SNPs located on autosomes 1–39 and the Z chromosome were retained. After quality control, 330 individuals and 9,843,930 SNPs remained for subsequent analyses, of which 9,434,258 autosomal SNPs were used to construct the genomic relationship matrix.

Genomic (SNP-based) heritabilities of MeL and MeC were obtained from the null linear mixed model fitted in GEMMA v0.98.5 before marker testing [7,19]. The model jointly analyzed the two traits, included line as a fixed effect and BW as a covariate, and fitted an individual polygenic effect using the genomic relationship matrix constructed from quality-controlled autosomal SNPs. For each trait, genomic heritability was calculated from the corresponding diagonal elements of the estimated genetic and residual covariance matrices.

*y* = *Xb* + *Za* + *e* (1)

where *y* is the phenotype vector for MeL or MeC; *X* is the design matrix for the fixed effects and covariates, including the intercept, line, and BW; *b* is the corresponding vector of fixed-effect parameters and regression coefficients; *Z* is the incidence matrix linking phenotypic records to individual additive genetic effects; *a* is the vector of individual additive genetic effects; and *e* is the vector of random residuals. The random effects were assumed to follow:

$$a∼N\left(0,G\sigma_{a}^{2}\right),e∼N\;(0,I\sigma_{e}^{2})$$

where *G* is the genomic relationship matrix constructed from autosomal SNP genotypes, *I* is an identity matrix, and $\sigma_{a}^{2}$ and $\sigma_{e}^{2}$ denote the additive genetic variance and residual variance, respectively. Genomic heritability for MeL and MeC was calculated as:

(2) $$h^{2}=\frac{\sigma_{a}^{2}}{\sigma_{a}^{2}+\sigma_{e}^{2}}$$

#### 2.2.2. Single-Trait and Bivariate Genome-Wide Association Analyses

Single-trait GWASs were performed separately for MeL and MeC using linear mixed models implemented in GEMMA v0.98.5. A bivariate MeL–MeC GWAS was subsequently conducted using a multivariate linear mixed model [7,19].

The single-trait GWAS model was expressed as:

*y* = *Wα* + *xβ* + *u* + *ε* (3)

where *y* is the phenotype vector for MeL or MeC; *W* the design matrix for fixed effects and covariates, including the intercept, line, and BW; *α* is the corresponding vector of fixed-effect parameters and regression coefficients; *x* is the genotype dosage vector for the SNP being tested; *β* is the additive effect of the tested SNP; *u* is the vector of individual polygenic random effects; and *ε* is the vector of random residuals. The random effects were assumed to follow:

$$\mu \;\sim \;N\left(0,K\sigma_{g}^{2}\right),\varepsilon \;\sim \;N\;(0,I\sigma_{e}^{2})$$

where *K* is the genomic relationship matrix constructed from the quality-controlled autosomal SNPs, *I* is an identity matrix, and $\sigma_{g}^{2}$ and $\sigma_{e}^{2}$ denote the polygenic and residual variances, respectively.

The bivariate GWAS jointly fitted MeL and MeC using the following model:

*Y* = *WA* + *xβ^T^* + *U* + *E* (4)

where *Y* is the phenotype matrix containing MeL and MeC; *W* is the design matrix for fixed effects and covariates, including the intercept, line, and BW; *A* is the corresponding matrix of fixed-effect parameters and regression coefficients; *x* is the genotype dosage vector for the SNP being tested; *β* is the vector of SNP effects on MeL and MeC; and *U* and *E* are the matrices of polygenic random effects and residuals, respectively. The random effects were assumed to follow:

*U* ~ *MN*(0, *K*, *V_g_*), *E* ~ *MN* (0, *I*, *V_e_*)

where *V_g_* and *V_e_* are the genetic and residual covariance matrices for MeL and MeC, respectively.

Wald tests were used for both the single-trait and bivariate GWASs. SNPs located on autosomes 1–39 and the Z chromosome were included in the association analyses, whereas only quality-controlled autosomal SNPs were used to construct the genomic relationship matrix. Multiple testing was considered using Bonferroni correction based on the number of SNPs tested, corresponding to a genome-wide significance threshold of approximately 5.08 × 10^−9^ (0.05/9,843,930). Because whole-genome resequencing generates a large number of highly correlated markers due to linkage disequilibrium, this Bonferroni threshold is highly conservative. Therefore, *p* < 1 × 10^−5^ was additionally used as a suggestive association threshold for candidate-locus discovery and subsequent integration with genetic differentiation and nucleotide diversity analyses. SNPs meeting this threshold were regarded as suggestive rather than genome-wide significant associations. Manhattan and Q–Q plots were generated using Matplotlib in Python v3.11, and the genomic inflation factor *λ*_GC_ was calculated to evaluate systematic deviations in the association test results.

#### 2.2.3. Genetic Differentiation and Nucleotide Diversity Analyses Based on Extreme Phenotypic Groups

To reduce the effects of BW and line-specific genetic background on extreme-phenotype grouping, MeL and MeC were first adjusted separately within each line using linear regression with BW as a covariate. The resulting residuals were used as the adjusted phenotypic values for the corresponding traits. Within each line, individuals were subsequently ranked from the highest to the lowest residual value, and the highest and lowest 10% were assigned to the high- and low-phenotype groups, respectively. The high- and low-phenotype groups each contained 16 individuals from the high-fat line and 17 individuals from the low-fat line. After pooling individuals with the same phenotypic direction across the two lines, the high- and low-phenotype groups for each trait each contained 33 individuals and had identical line compositions.

Using autosomal SNP genotype data from the high- and low-phenotype groups, weighted Weir–Cockerham fixation index (*F_ST_*) values were calculated between the two groups using VCFtools v0.1.16 [20]. Nucleotide diversity (*π*) was also calculated separately within the high- and low-phenotype groups. The *F_ST_* statistic was used to quantify genetic differentiation between the two extreme phenotypic groups, whereas *π* was used to measure genetic diversity within each group.

Both *F_ST_* and *π* were calculated using a sliding-window approach, with a window size of 100 kb and a step size of 10 kb. To reduce statistical instability caused by low SNP density, only windows containing at least 50 valid SNPs in the *F_ST_*, high-phenotype-group *π*, and low-phenotype-group *π* analyses were retained.

Weighted *F_ST_* values for the retained windows were standardized using a *Z* transformation:

(5) $$Z_{F_{\mathrm{ST}}}=\frac{F_{\mathrm{ST}}-\bar{F_{\mathrm{ST}}}}{\mathrm{SD}\;(F_{\mathrm{ST}})}$$

The nucleotide diversity ratio between the low- and high-phenotype groups was calculated as:

(6) $$R_{\pi }=\log_{2}\;\left(\frac{\pi_{\mathrm{low}}}{\pi_{\mathrm{high}}}\right)$$

where *π_low_* and *π_high_* represent nucleotide diversity in the low- and high-phenotype groups, respectively. Windows within the upper 5% of the genome-wide $Z_{F_{\mathrm{ST}}}$ distribution were defined as highly differentiated windows. For *R_π_*, windows within the lower 5% indicated relatively reduced nucleotide diversity in the low-phenotype group, whereas windows within the upper 5% indicated relatively reduced nucleotide diversity in the high-phenotype group.

Candidate differentiated windows were defined using the following criteria: (i) windows within the upper 5% of $Z_{F_{\mathrm{ST}}}$ and the lower 5% of *R_π_* were classified as candidate regions associated with the low-phenotype group; and (ii) windows within the upper 5% of both $Z_{F_{\mathrm{ST}}}$ and *R_π_* were classified as candidate regions associated with the high-phenotype group. Overlapping candidate windows associated with the same phenotypic direction were merged to obtain nonredundant candidate differentiated regions. Joint *F_ST_*–*π* plots and the corresponding frequency and cumulative distributions of $Z_{F_{\mathrm{ST}}}$ and *R_π_* were generated using R.

#### 2.2.4. Integration of GWAS and Genetic Differentiation Signals

To integrate the complementary genetic evidence provided by the single-trait GWASs, the bivariate GWAS, and the genetic differentiation analysis of the extreme phenotypic groups, trait-specific GWAS candidate SNP sets were constructed separately for MeL and MeC and subsequently matched to the corresponding candidate differentiated regions based on their genomic positions. All GWAS and *F_ST_*–*π* analyses were conducted using the same 330 individuals from the study population; therefore, their overlap was considered complementary evidence from different analytical approaches rather than independent replication. All results were based on the *Gallus gallus* GRCg7b reference genome, and chromosome names and genomic coordinate formats were standardized before the integrative analysis.

For MeL, SNPs reaching the suggestive association threshold (*p* < 1 × 10^−5^) in either the MeL single-trait GWAS or the MeL–MeC bivariate GWAS were combined. Duplicate variants were removed according to chromosome and physical position to construct the MeL GWAS candidate SNP set. The same procedure was applied to MeC by combining and deduplicating SNPs reaching the suggestive association threshold in the MeC single-trait GWAS or the MeL–MeC bivariate GWAS. The source of evidence for each SNP was retained, and the candidate SNPs were classified into three categories: SNPs detected exclusively by the single-trait GWAS, SNPs detected exclusively by the bivariate GWAS, and SNPs detected by both analyses.

The GWAS candidate SNP sets for MeL and MeC were then matched separately to the candidate differentiated regions identified for the corresponding trait. Specifically, MeL GWAS candidate SNPs were matched only to MeL candidate differentiated regions, whereas MeC GWAS candidate SNPs were matched only to MeC candidate differentiated regions. SNPs that reached the suggestive association threshold in either the single-trait or bivariate GWAS and were also located within a candidate differentiated region for the corresponding trait were defined as prioritized candidate SNPs jointly supported by the GWAS and genetic differentiation analyses.

Finally, the prioritized candidate SNP sets for MeL and MeC were compared to identify trait-specific candidate SNPs and candidate SNPs shared by the two traits. These prioritized candidate SNPs were subsequently used for candidate-region refinement, local linkage disequilibrium analysis, functional variant annotation, and candidate gene identification.

#### 2.2.5. Candidate-Region Refinement and Linkage Disequilibrium Analysis

Initial candidate intervals were defined by extending 500 kb upstream and downstream from each jointly prioritized candidate SNP. Overlapping intervals located on the same chromosome were merged to generate nonredundant initial candidate regions, which were constructed and analyzed separately for MeL and MeC. Genome-wide LD decay was evaluated in the study population, and the resulting LD-decay pattern is shown in Supplementary Figure S2. The ±500 kb interval was used as an initial candidate-region window to capture nearby potentially relevant variants, rather than to define the physical extent of LD.

Within each initial candidate region, jointly prioritized SNPs showing low pairwise LD with one another (*r*^2^ < 0.2) were assigned to different LD-defined candidate signals. For each LD-refined signal, the SNP with the smallest *p*-value across the single-trait and bivariate GWAS analyses was defined as the lead SNP. Linkage disequilibrium between the lead SNP and the other SNPs within the corresponding signal was calculated using PLINK v1.90b6.21 and expressed as the squared genotype correlation coefficient (*r*^2^). SNPs were classified as being in high (*r*^2^ ≥ 0.8), moderate (0.2 ≤ *r*^2^ < 0.8), or low (*r*^2^ < 0.2) LD with the lead SNP.

Local association plots were generated by integrating GWAS association strength, physical position, and LD relationships among SNPs within each signal to visualize the local association pattern and LD structure.

#### 2.2.6. Functional Annotation of Variants and Candidate Gene Identification

Functional annotation of the prioritized candidate SNPs jointly supported by the GWAS and genetic differentiation analyses was performed using SnpEff v4.3.1t based on the *Gallus gallus* GRCg7b reference genome and the GFF3 gene annotation file from Ensembl release 115 [21]. According to their genomic positions and predicted effects on gene structure, SNPs were annotated as intergenic, upstream, downstream, intronic, exonic, untranslated-region, or splice-related variants. SNPs located in protein-coding regions were further classified as synonymous, missense, or other variants potentially affecting protein structure.

Candidate genes were classified into two categories according to their physical relationships with the prioritized SNPs. Direct candidate genes included genes containing prioritized SNPs within their gene bodies and genes located within 5 kb upstream or downstream of a prioritized SNP. Regional candidate genes included genes overlapping candidate regions or genomic intervals containing SNPs in high linkage disequilibrium with the lead SNP. For prioritized intergenic SNPs that could not be assigned to a gene within 5 kb, the nearest gene within 500 kb upstream or downstream was identified and classified as a regional candidate gene.

Direct and regional candidate genes were summarized separately for MeL and MeC. The candidate gene sets for the two traits were subsequently compared to identify trait-specific genes and genes jointly supported for both traits.

#### 2.2.7. Construction of Genomic Prediction Models and Evaluation of Candidate-Marker Performance

Genomic breeding values were estimated separately for MeL and MeC. To compare the predictive performance of different models, GBLUP, BayesA, BayesB, BayesCπ, Bayesian LASSO, and RKHS models were fitted. Because Bayesian models estimate individual marker effects during Markov chain Monte Carlo sampling, repeated cross-validation using the full-density genotype data would be computationally intensive. Therefore, highly redundant SNPs in strong linkage disequilibrium were first removed, followed by further thinning at 100 kb intervals according to genomic position. This procedure retained 10,305 LD-pruned and position-thinned SNPs that were approximately evenly distributed across the genome. The same set of 10,305 SNPs was used to train and evaluate all six models, ensuring a fair comparison of predictive performance among models.

BayesA, BayesB, BayesCπ, Bayesian LASSO, and RKHS were implemented in R 4.2.3 using the BGLR v1.1.2 package [22]. Each of these five models was run for 12,000 Markov chain Monte Carlo (MCMC) iterations, with the first 3000 iterations discarded as burn-in and a thinning interval of 4, resulting in 2250 retained posterior samples. Marker-inclusion probabilities in BayesB and BayesCπ were estimated from their posterior distributions, with the initial inclusion probability in BayesB set to 0.5. For Bayesian LASSO, minAbsBeta was set to 1 × 10^−6^ to reduce numerical instability caused by marker effects approaching zero. The RKHS model used a single Gaussian radial basis kernel with a data-adaptive bandwidth. The bandwidth was set to the median of the nonzero pairwise mean squared Euclidean distances among individuals in the corresponding training set and was re-estimated within each cross-validation training fold.

To evaluate the predictive performance of trait-associated candidate markers, candidate marker unions were constructed separately for MeL and MeC. For each trait, the candidate marker union consisted of three groups of SNPs: SNPs reaching the suggestive association threshold (*p* < 1 × 10^−5^) in the corresponding single-trait GWAS, SNPs reaching the same threshold in the MeL–MeC bivariate GWAS, and all quality-controlled SNPs located within the corresponding candidate differentiated regions. The SNPs from these three sources were merged and duplicate loci were removed according to chromosome and physical position. The candidate marker unions were constructed using GWAS and genetic differentiation results obtained from all 330 individuals. The candidate-marker sets were constructed from the full dataset because this analysis was intended as an internal assessment of whether the loci identified by the integrative genomic analyses were enriched for trait-relevant predictive information within the study population, rather than as an unbiased estimate of prediction performance in independent individuals.

To determine whether the predictive performance of the candidate markers was attributable solely to marker number, chromosomal distribution, or allele-frequency structure, five random SNP control sets were generated separately for MeL and MeC. Each random SNP set contained the same number of SNPs as the corresponding candidate marker union and was matched by chromosome and minor allele frequency (MAF). MAF was divided into bins with a width of 0.05, and the required number of SNPs was randomly sampled from the corresponding chromosome and MAF bin.

Additive genomic relationship matrices were constructed for the different marker sets using the --make-xrm --add option in HIBLUP v1.6.0 [23]. The genome-wide relationship matrix was constructed using all quality-controlled SNPs, the candidate-marker relationship matrix was constructed using the candidate marker union for the corresponding trait, and five random-marker relationship matrices were constructed using the respective random SNP sets. In addition, the background-marker relationship matrix was constructed using the remaining genome-wide SNPs after excluding the corresponding candidate marker union.

GBLUP and genomic feature best linear unbiased prediction (GFBLUP) models were fitted using ASReml-R v4.2 [24]. Genome-wide-marker GBLUP, candidate-union GBLUP, and random-control GBLUP each fitted a single additive genomic relationship matrix constructed from the corresponding marker set. GFBLUP jointly fitted the candidate-marker and background-marker relationship matrices to separately estimate the genomic effects explained by the candidate markers and the remaining genome-wide markers [25]. For genomic prediction models, line and BW were included as fixed effects to account for systematic differences among selection lines and families. Genome-wide-marker GBLUP was used as the reference model in both the comparison of prediction models and the comparison of marker sets.

All models were evaluated using repeated fivefold cross-validation with 10 repetitions. Cross-validation folds were assigned according to sire family, and all individuals from the same sire family were retained within the same fold. Identical training and validation partitions were used for all models and marker sets within each repetition. Predictive ability (*r*) was defined as the Pearson correlation between genomic estimated breeding values (GEBVs) and phenotypic values adjusted for line and BW in the validation individuals. A heritability-adjusted prediction estimate was then calculated as:

(7) $$\mathrm{Heritability}-\mathrm{adjusted}\;\mathrm{prediction}\;\mathrm{estimate}\;=\frac{r}{\sqrt{h^{2}}}$$

where (*h*^2^) is the trait-specific genomic heritability estimated using all 330 individuals. Because (*h*^2^) was estimated from the complete dataset, this transformed measure should be interpreted as a derived, heritability-adjusted estimate rather than a fully independent estimate of out-of-sample prediction accuracy. Root mean square error (RMSE), mean absolute error (MAE), mean prediction bias, and calibration slope were also calculated as supplementary measures of the internal predictive performance of the different models and marker sets.

Detailed information on the bioinformatics and statistical software used in this study, including version numbers, websites/URLs, and access dates, is provided in Supplementary Table S3.

## 3. Results

### 3.1. Descriptive Statistics and Heritability Estimates for Metatarsal Traits

Descriptive statistics and genomic heritability estimates for MeL and MeC are summarized in Table 1. Genomic heritability estimates were 0.266 for MeL and 0.516 for MeC, with a higher point estimate for MeC than for MeL. MeL and MeC showed a strong negative phenotypic correlation (r = −0.77), whereas the estimated genetic correlation was 0.19. These estimates provide context for the bivariate GWAS, but the genetic correlation should be interpreted cautiously given the sample size. Principal component analysis based on genome-wide SNPs revealed genetic differentiation between the HAF and LAF lines, consistent with their divergent selection history (Supplementary Figure S1).

### 3.2. Genome-Wide Association Analyses for MeL and MeC

#### 3.2.1. Single-Trait GWAS for MeL

The single-trait GWAS for MeL identified 102 SNPs reaching the suggestive association threshold of *p* < 1 × 10^−5^. The most strongly associated SNP was 15_4577235 on chromosome 15 (*p* = 1.97 × 10^−7^), followed by 13_3982321 on chromosome 13 (*p* = 2.58 × 10^−7^) and 4_7903200 on chromosome 4 (*p* = 4.51 × 10^−7^). Additional suggestive association signals were observed on chromosomes 1, 3, 6, and 17, as well as on the Z chromosome.

**Figure 1:**
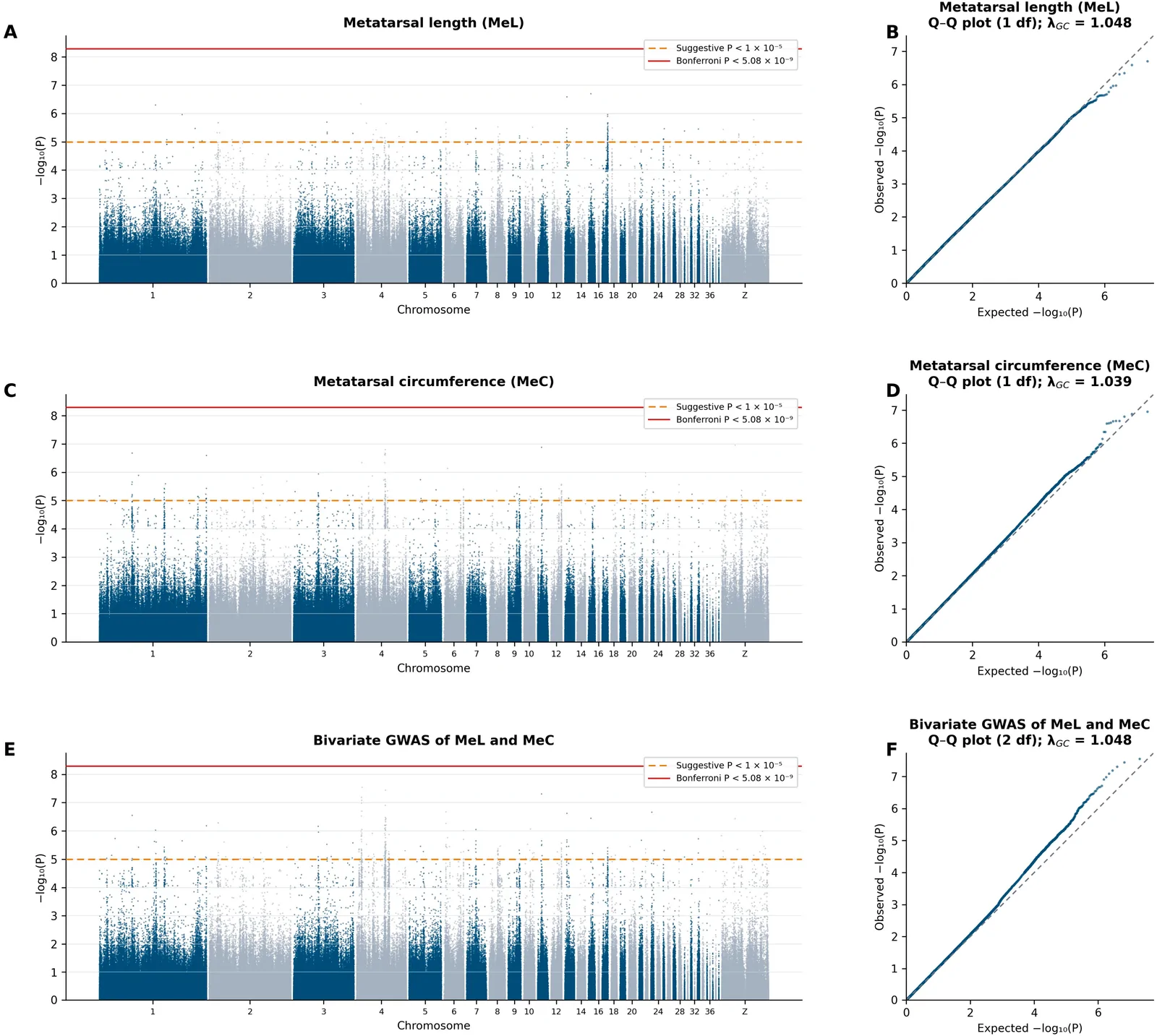
Genome-wide association analyses of MeL and MeC using single-trait and bivariate models. Note: (**A**) Manhattan plot of the single-trait GWAS for MeL; (**B**) Q–Q plot of the single-trait GWAS for MeL; (**C**) Manhattan plot of the single-trait GWAS for MeC; (**D**) Q–Q plot of the single-trait GWAS for MeC; (**E**) Manhattan plot of the bivariate GWAS for MeL and MeC; and (**F**) Q–Q plot of the bivariate GWAS for MeL and MeC. Alternating blue and gray colors are used to distinguish adjacent chromosomes (autosomes 1–39 and the Z chromosome) in the Manhattan plots. The orange dashed line indicates the suggestive association threshold of *p* = 1 × 10^−5^, while the red solid line indicates the Bonferroni-corrected genome-wide significance threshold (*p* ≈ 5.08 × 10^−9^). In the Q–Q plots, the dashed line represents the expected distribution under complete agreement between the observed and theoretical *p*-values.

The Manhattan plot showed that the association signals for MeL were distributed across multiple chromosomes (Figure 1A). In the Q–Q plot, the observed values for most SNPs closely followed the theoretical expectation, with no obvious systematic deviation. An upward deviation was observed only in the upper tail of the distribution (Figure 1B), consistent with the presence of association signals at a subset of loci.

#### 3.2.2. Single-Trait GWAS for MeC

The single-trait GWAS for MeC identified 165 SNPs reaching the suggestive association threshold. The strongest association signal was located at 24,478,892 bp on the Z chromosome and corresponded to SNP Z_24478892 (*p* = 1.10 × 10^−7^). This was followed by 11_7373893 on chromosome 11 (*p* = 1.29 × 10^−7^) and 4_52498560 on chromosome 4 (*p* = 1.56 × 10^−7^).

Several additional suggestively associated SNPs with relatively low *p*-values were detected on chromosomes 1 and 4. In particular, association signals were concentrated within the approximately 50.9–52.5 Mb region on chromosome 4, suggesting that this region may contain genetic variants associated with phenotypic variation in MeC. The Manhattan and Q–Q plots for MeC are shown in Figure 1C and Figure 1D, respectively. In the Q–Q plot, the observed values for most SNPs were generally consistent with the theoretical expectation, with an upward deviation occurring only in the upper tail of the distribution.

#### 3.2.3. Bivariate GWAS for MeL and MeC

To evaluate the joint genetic associations of MeL and MeC, a bivariate GWAS was further conducted. A total of 263 SNPs reached the suggestive association threshold of *p* < 1 × 10^−5^. The strongest association signal was located at 9,887,179 bp on chromosome 4 and corresponded to SNP 4_9887179 (*p* = 2.82 × 10^−8^), which had a lower *p*-value than the most significant SNPs identified in either single-trait GWAS. The Manhattan and Q–Q plots for the bivariate GWAS are shown in Figure 1E and Figure 1F, respectively.

**Figure 2:**
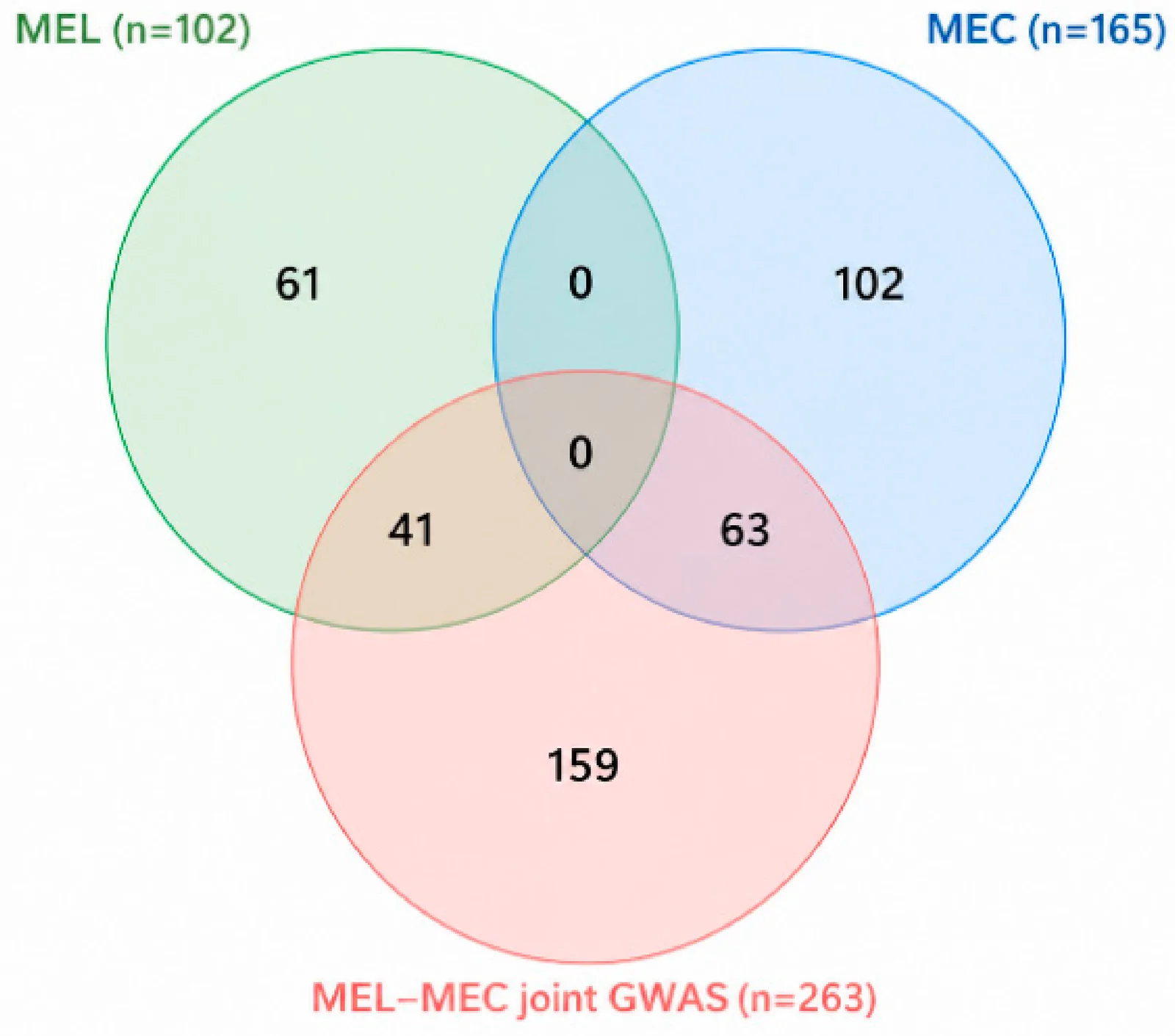
Venn diagram of SNPs reaching the suggestive threshold in the single-trait and bivariate GWAS analyses for MeL and MeC. Note: The suggestive association threshold was set at *p* < 1 × 10^−5^. Numbers within the circles indicate the numbers of suggestively associated SNPs that were unique to or shared among the three GWAS analyses.

Among the 263 SNPs identified in the bivariate GWAS, 41 also reached the suggestive threshold in the single-trait GWAS for MeL and 63 did so for MeC. Thus, 104 SNPs overlapped with at least one single-trait analysis, whereas 159 were detected only by the bivariate analysis. No suggestive SNP was shared directly between the two single-trait GWASs (Figure 2). This absence of exact SNP overlap does not imply that MeL and MeC are unrelated: the traits were phenotypically correlated, but distinct lead markers can arise when their architectures are partly trait specific and when correlated signals are captured more efficiently by the bivariate model.

### 3.3. Joint Analysis Based on F_ST_ and Nucleotide Diversity

#### 3.3.1. Candidate Regions for MeL

A total of 93,620 autosomal windows meeting the filtering criteria were obtained for MeL. Joint screening based on standardized weighted *F_ST_* and the nucleotide diversity ratio identified 1902 candidate windows, of which 831 showed reduced nucleotide diversity in the low-phenotype group and 1071 showed reduced nucleotide diversity in the high-phenotype group.

**Figure 3:**
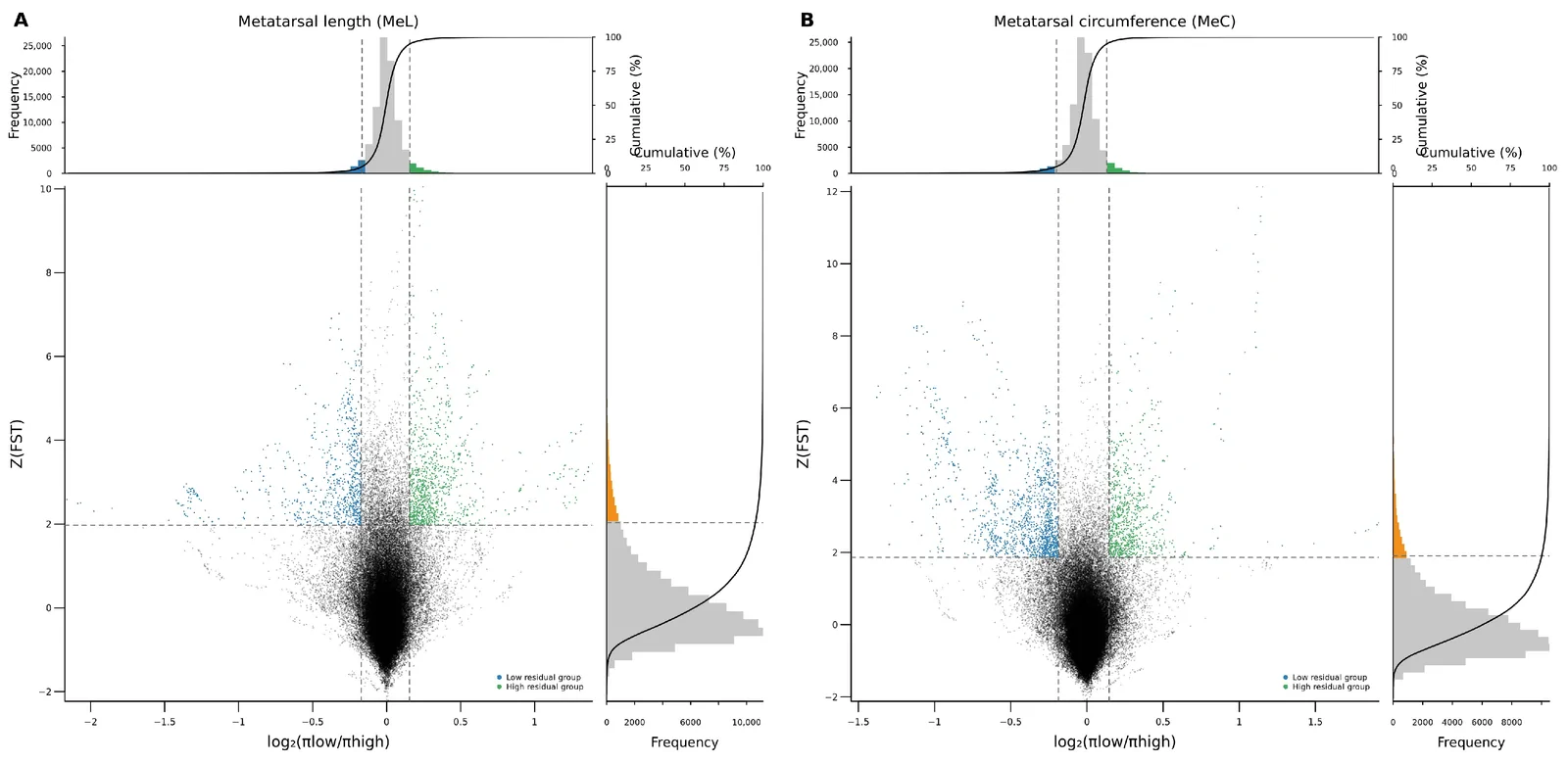
Joint analysis of genetic differentiation and nucleotide diversity for MeL and MeC. Note: (**A**) Joint distribution of *F_ST_* and the nucleotide diversity ratio for MeL; (**B**) joint distribution of *F_ST_* and the nucleotide diversity ratio for MeC. The x-axis represents the log-transformed nucleotide diversity ratio between the low- and high-phenotype groups, and the y-axis represents standardized weighted *F_ST_* values. The horizontal dashed line indicates the upper 5% threshold for *F_ST_*, whereas the two vertical dashed lines indicate the lower and upper 5% thresholds for the nucleotide diversity ratio. Blue points represent candidate windows with reduced nucleotide diversity in the low-phenotype group, green points represent candidate windows with reduced nucleotide diversity in the high-phenotype group, and black points represent windows that did not simultaneously meet the joint screening criteria. The upper and right panels show the frequency and cumulative distributions of the nucleotide diversity ratio and standardized *F_ST_*, respectively.

Because a window size of 100 kb and a step size of 10 kb were used, adjacent windows overlapped by 90 kb. Candidate windows that overlapped in genomic position and corresponded to the same phenotypic group were therefore merged. After merging, 203 candidate differentiated regions were identified for MeL, including 98 regions corresponding to the low-phenotype group and 105 regions corresponding to the high-phenotype group. These regions collectively spanned approximately 38.76 Mb and contained 315,202 nonredundant quality-controlled SNPs. The joint distribution of *F_ST_* and the nucleotide diversity ratio for MeL is shown in Figure 3A.

#### 3.3.2. Candidate Regions for MeC

A total of 93,615 autosomal windows meeting the filtering criteria were obtained for MeC. Joint screening based on standardized weighted *F_ST_* and the nucleotide diversity ratio identified 2324 candidate windows, of which 1423 showed reduced nucleotide diversity in the low-phenotype group and 901 showed reduced nucleotide diversity in the high-phenotype group.

After candidate windows that overlapped in genomic position and corresponded to the same phenotypic group were merged, 186 candidate differentiated regions were identified for MeC. These included 93 regions corresponding to the low-phenotype group and 93 regions corresponding to the high-phenotype group, collectively spanning approximately 42.12 Mb. These regions contained 342,143 nonredundant quality-controlled SNPs. The joint distribution of *F_ST_* and the nucleotide diversity ratio for MeC is shown in Figure 3B.

### 3.4. Integration of GWAS and F_ST_–π Analyses and Refinement of Candidate Regions

To integrate association evidence from GWAS with evidence of genetic differentiation and nucleotide diversity between the extreme phenotypic groups, suggestively associated SNPs identified by the single-trait and bivariate GWAS analyses were overlapped with the trait-specific *F_ST_*–*π* candidate regions. Candidate variants and genes were subsequently prioritized through candidate-region construction, linkage disequilibrium analysis, functional annotation of variants, and gene mapping.

#### 3.4.1. Candidate SNPs Prioritized by Integrating GWAS and *F_ST_*–*π* Analyses

After merging the 102 suggestively associated SNPs identified by the single-trait GWAS for MeL with the 263 SNPs identified by the MeL–MeC bivariate GWAS and removing duplicate loci, 324 SNPs were retained in the MeL GWAS union set. Among these, 55 autosomal SNPs were located within the *F_ST_*–*π* candidate regions for MeL and were retained as jointly prioritized candidate SNPs. These included 17 SNPs unique to the single-trait GWAS for MeL, 32 SNPs unique to the bivariate GWAS, and six SNPs detected by both GWAS analyses. Because the *F_ST_*–*π* analysis was restricted to autosomes, one additional GWAS candidate SNP on the Z chromosome was not included in the joint-screening count and was retained separately as a supplementary candidate. The regional association and linkage disequilibrium pattern for this signal is shown in Supplementary Figure S3.

Integration of the GWAS and *F_ST_*–*π* results substantially narrowed the candidate sets for both traits, resulting in 55 prioritized candidate SNPs for MeL and 62 for MeC (Table 2). Comparison of the jointly prioritized candidate SNP sets for MeL and MeC identified three shared loci: 4_59470884, 4_59483846, and 4_59544384. All three loci were detected by the bivariate GWAS and were located within the respective *F_ST_*–*π* candidate regions for both MeL and MeC. These loci were therefore considered shared candidate variants potentially associated with both traits.

#### 3.4.2. Delineation of Candidate Regions and LD Structure Analysis

A 500 kb region upstream and downstream of each jointly screened candidate SNP was defined, and regions overlapping in genomic position were merged. This resulted in 20 initial autosomal candidate regions for MeL and 14 initial autosomal candidate regions for MeC.

**Figure 4:**
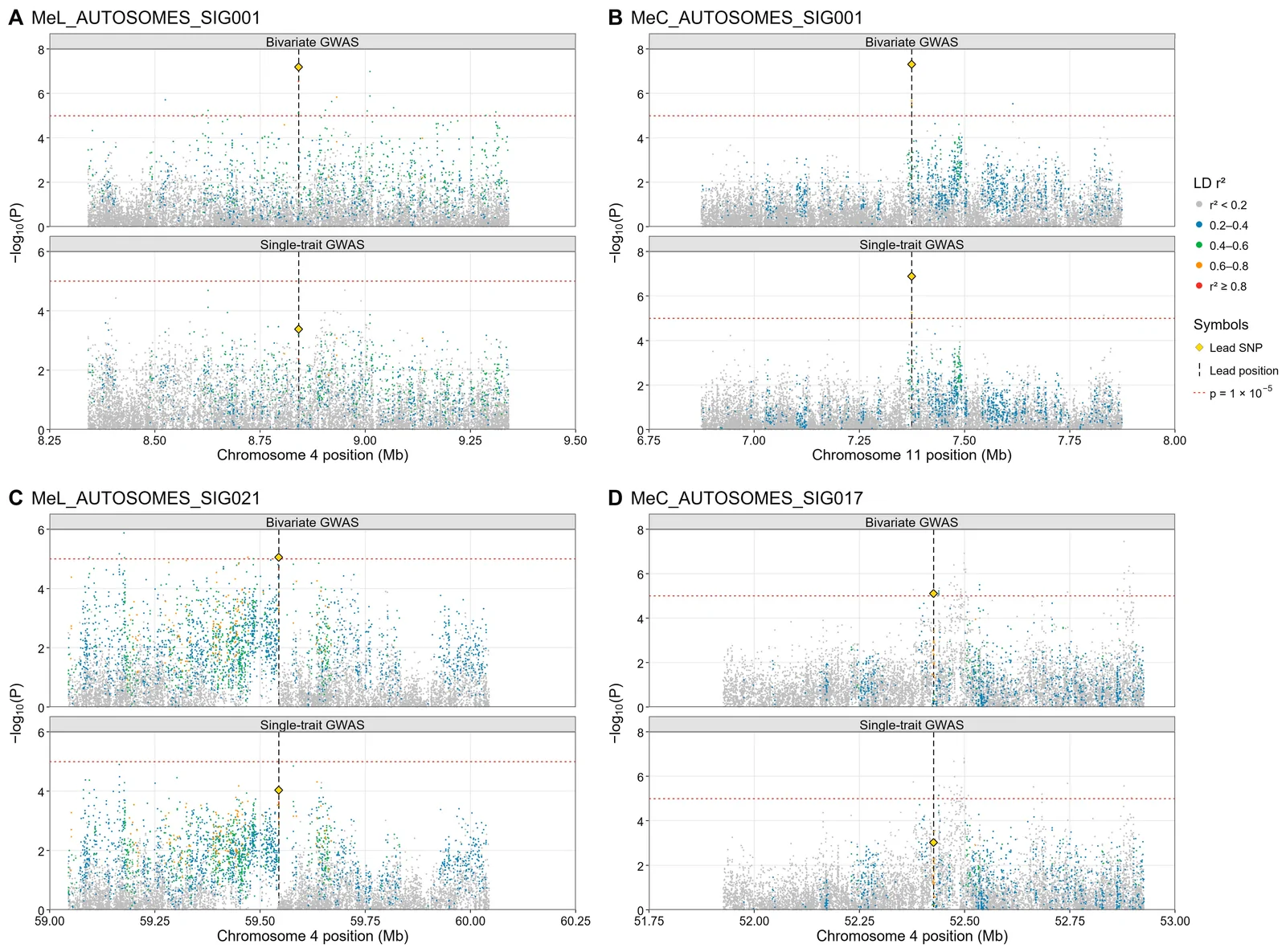
Local association signals and LD structures of representative candidate regions. Note: (**A**) MeL_AUTOSOMES_SIG001, the strongest association signal for MeL; (**B**) MeC_AUTOSOMES_SIG001, the strongest association signal for MeC; (**C**) MeL_AUTOSOMES_SIG021, containing three candidate SNPs shared between MeL and MeC; and (**D**) MeC_AUTOSOMES_SIG017, containing a candidate missense variant in *ANKRD50*. The remaining 41 autosomal LD-refined signals are shown in Supplementary Figures S4 and S5. The yellow diamonds indicate the lead SNPs, and the black vertical dashed lines indicate their genomic positions. The red horizontal dotted lines represent the suggestive association threshold (*p* = 1 × 10^−5^). The colors of the points indicate the degree of linkage disequilibrium (LD r^2^) between each SNP and the corresponding lead SNP, as shown in the legend.

The initial candidate regions were further refined according to the linkage disequilibrium (LD) relationships among the jointly screened candidate SNPs within each region. A total of 45 autosomal LD-defined candidate signals were identified, including 26 for MeL and 19 for MeC. Within each LD-refined signal, the SNP with the lowest *p*-value across the single-trait and bivariate GWAS results was defined as the lead SNP. The strongest refined signal for MeL was MeL_AUTOSOMES_SIG001, with 4_8841620 as the lead SNP and a minimum *p*-value of 6.49 × 10^−8^. The strongest refined signal for MeC was MeC_AUTOSOMES_SIG001, with 11_7373893 as the lead SNP and a minimum *p*-value of 4.91 × 10^−8^. Figure 4 presents four representative candidate signals, including the strongest signals for MeL and MeC, a region containing candidate SNPs shared between the two traits, and a region containing a potentially functional variant. Local association and LD patterns for the remaining 41 autosomal LD-defined candidate signals are presented in Supplementary Figures S4 and S5.

#### 3.4.3. Functional Annotation of Candidate Variants and Identification of Candidate Genes

**Figure 5:**
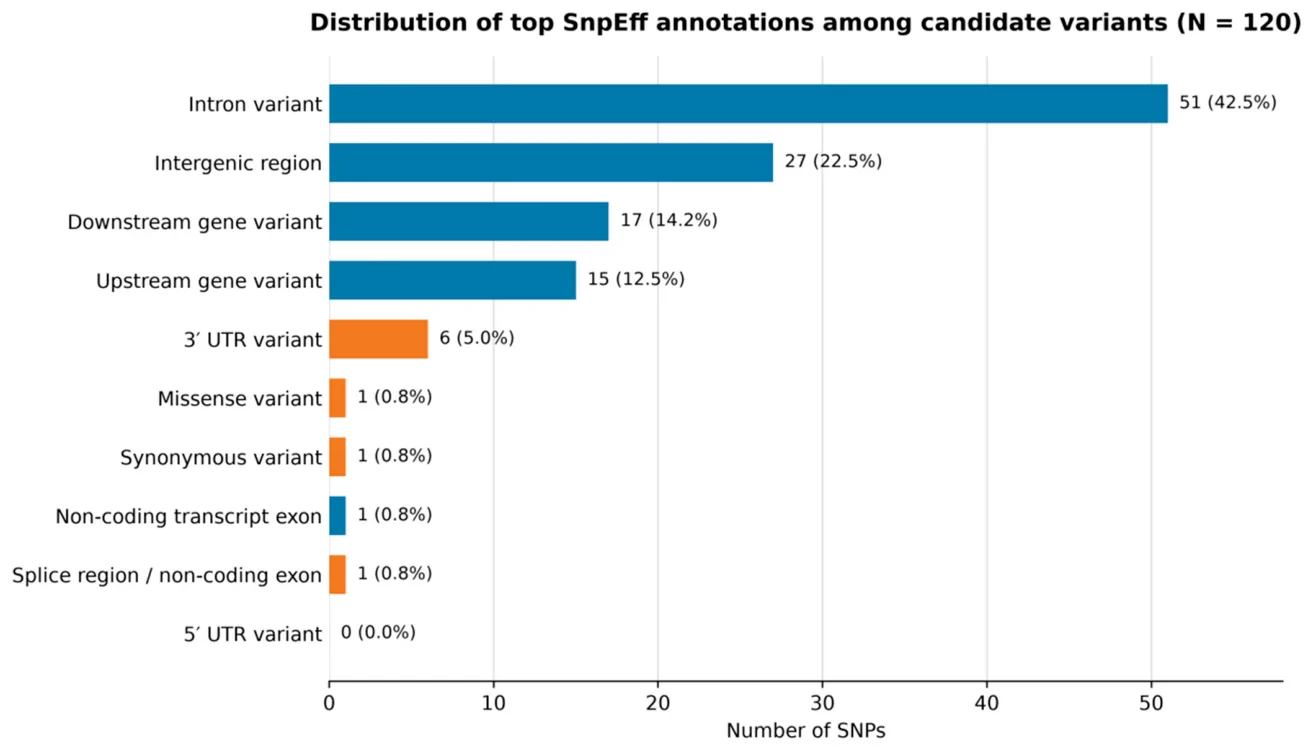
Distribution of the major SnpEff functional annotation categories for the 120 candidate variants.

After merging the jointly screened candidate SNPs with variants in high LD and removing duplicate loci, a total of 120 candidate variants were retained. Each variant was assigned to a mutually exclusive category according to its most severe SnpEff annotation. Intronic variants were the most abundant, accounting for 51 variants (42.50%), followed by 27 intergenic variants (22.50%), 17 downstream gene variants (14.17%), 15 upstream gene variants (12.50%), and six 3′UTR variants (5.00%). Two coding exon variants were also identified, comprising one missense variant and one synonymous variant. An additional two variants were annotated as non-coding transcript exon- or splice-region-related variants, whereas no 5′UTR variants were detected (Figure 5).

According to the functional impact categories predicted by SnpEff, 117 candidate variants were classified as MODIFIER, two as LOW, and one as MODERATE, whereas no HIGH-impact variants were identified. The MeC candidate SNP 4_52426323 was a missense variant in *ANKRD50* and was the only variant predicted to have a MODERATE impact. SNP 4_52438421, located within the unnamed gene *ENSGALG00010005750*, was annotated as a splice-region/non-coding transcript exon variant and was classified as LOW impact. Among the three candidate SNPs shared between MeL and MeC, 4_59483846 was a synonymous variant in *ADH6*, 4_59544384 was located in the *C4H4orf54/MTTP* region and was annotated as a 3′UTR variant, and 4_59470884 was located in the intergenic region between *METAP1* and *ADH6*.

Based on candidate-variant positions and LD-region information, 64 nonredundant candidate genes were identified. Of these, 57 had direct variant-location evidence and 48 had LD-region evidence, with some genes supported by both types of evidence. A total of 38 and 30 candidate genes were identified for MeL and MeC, respectively. Four candidate genes were shared between the two traits: *ADH6*, *C4H4orf54*, *MTTP*, and *ENSGALG00010001631*. Table 3 summarizes the SNPs shared between MeL and MeC, variants with potential functional effects, and representative lead SNPs with relatively strong association evidence. Complete results for the candidate variants and genes are provided in Supplementary Table S1.

### 3.5. Comparison of Genomic Prediction Models and Evaluation of Candidate-Marker Performance

#### 3.5.1. Comparison of the Performance of Different Genomic Prediction Models

**Figure 6:**
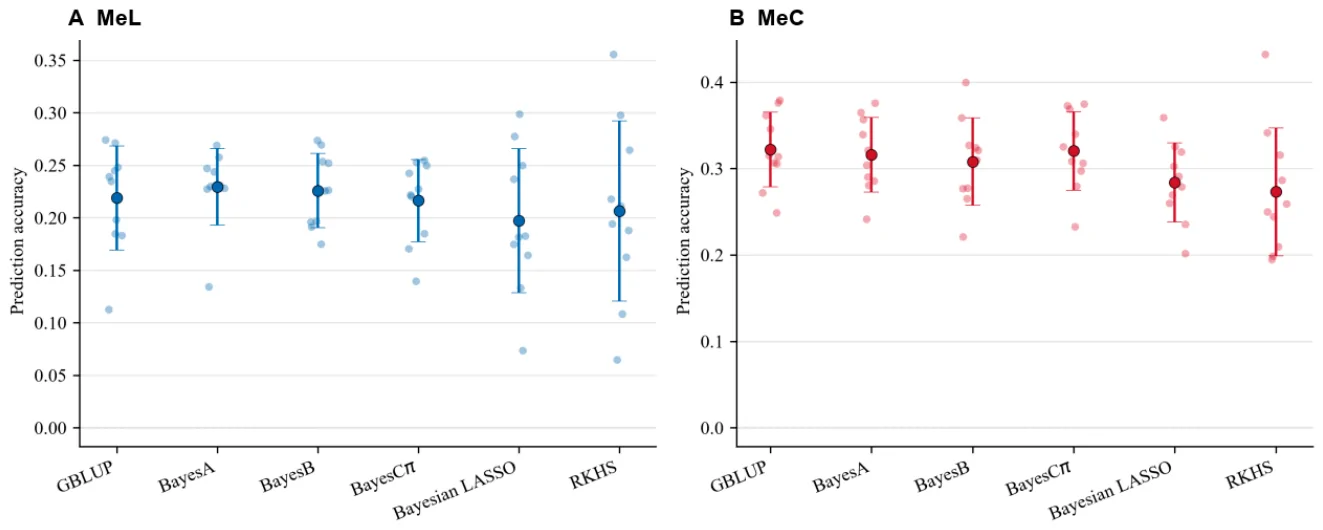
Genomic prediction accuracy of different models. Note: Small points represent the results from the 10 repeated cross-validation runs, whereas solid points and error bars represent the mean and standard deviation, respectively.

Using the same 10,305 LD-pruned and position-thinned SNPs, mean heritability-adjusted prediction estimates across the six models ranged from 0.197 to 0.230 for MeL and from 0.273 to 0.322 for MeC. BayesA yielded a mean accuracy of 0.230 for MeL, whereas GBLUP yielded 0.322 for MeC; however, the distributions across the 10 repeated cross-validation runs overlapped, indicating modest model-dependent differences rather than a consistent overall ranking. Results are shown in Figure 6 and Supplementary Table S2; RMSE, MAE, prediction bias, and calibration slope are provided in Supplementary Figures S6 and S7.

#### 3.5.2. Comparison of Predictive Performance Among Different Marker Sets

Using the full-density quality-controlled SNP set without LD pruning or position-based thinning, the internal predictive performance of full-density marker GBLUP, candidate-union GBLUP, GFBLUP, and matched-random-marker GBLUP was further compared.

**Figure 7:**
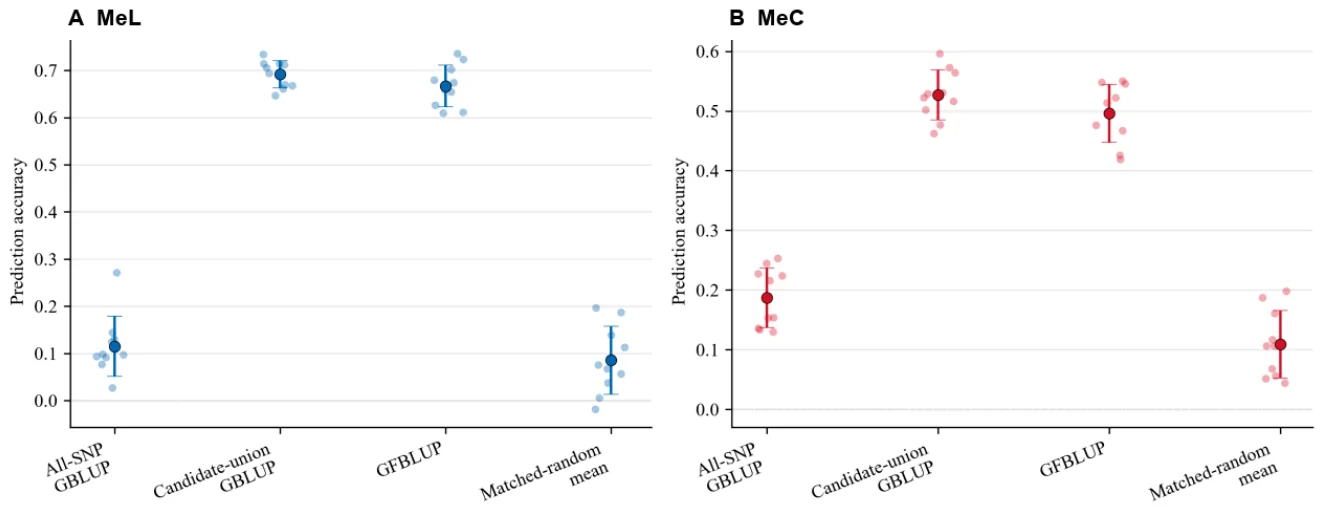
Comparison of prediction accuracy among full-density markers, candidate markers, GFBLUP, and matched random marker sets. Note: The matched-random-marker result represents the mean prediction accuracy across the five matched random marker sets.

For both MeL and MeC, candidate-union GBLUP and GFBLUP showed higher within-population predictive performance than full-density GBLUP and matched-random-marker GBLUP. Candidate-union GBLUP showed the highest mean prediction accuracy for both traits, followed by GFBLUP (Figure 7; Supplementary Table S2). Additional prediction metrics are provided in Supplementary Figures S8 and S9.

## 4. Discussion

This study was designed to characterize the genetic basis of MeL and MeC, identify candidate loci and genes, and evaluate the informativeness of integrated candidate-marker sets within the study population. The combination of single-trait and bivariate GWAS with genetic differentiation and nucleotide diversity analyses enabled the prioritization of 55 candidate SNPs for MeL and 62 for MeC, including three loci shared between the two traits, thereby addressing the objective of identifying trait-associated genomic regions. Subsequent LD-based refinement and functional annotation further narrowed these signals to 120 candidate variants and 64 candidate genes, providing biological context for the identified associations. In addition, comparison of genomic prediction models and marker sets showed that the integrated candidate-marker sets contained substantial trait-relevant information within the present population. Together, these findings addressed the major objectives of the study by linking locus discovery, candidate refinement, and internal evaluation of marker informativeness.

### 4.1. Phenotypic Variation and Heritability of MeL and MeC

MeL and MeC exhibited coefficients of variation of 5.82% and 6.28%, respectively, indicating measurable phenotypic variation in both traits within the study population. Their genomic heritabilities were estimated at 0.266 for MeL and 0.516 for MeC, suggesting a moderate genomic contribution to variation in MeL and a relatively stronger genetic contribution to variation in MeC. The higher heritability of MeC indicates that a greater proportion of its phenotypic variance was attributable to additive genomic effects under the present population structure and statistical model.

Previous studies using the same divergently selected broiler lines reported heritability estimates of approximately 0.24–0.37 for metatarsal traits [26], while another study reported an estimate of 0.18 for MeL and values above 0.30 for MeC [27]. Although the magnitude of these estimates varies among studies, the generally higher heritability reported for MeC is consistent with the pattern observed here. Such variation in heritability estimates may arise from differences in generation, age at measurement, population structure, sample size, phenotyping procedures, genomic marker density, and statistical models. Because heritability is population- and environment-specific, the estimates obtained in the present study should not be interpreted as fixed properties of the traits. Nevertheless, the moderate-to-high genomic heritabilities observed for MeL and particularly MeC indicate that both traits contain exploitable additive genetic variation and may respond to genomic selection.

### 4.2. Candidate Loci Identified Through Integrative Analyses and Their Potential Biological Relevance

The single-trait GWASs identified 102 suggestive SNPs for MeL and 165 for MeC, with no identical SNP detected for both traits. In contrast, the bivariate GWAS identified 263 associated SNPs, including 159 that were not detected by either single-trait analysis. Notably, all three loci ultimately shared between the prioritized MeL and MeC candidate sets were detected by the bivariate analysis. These results illustrate the additional information provided by joint analysis of correlated traits, which can increase power to detect loci contributing to their shared genetic architecture. Nevertheless, the shared loci identified here should be regarded as candidate regions rather than confirmed pleiotropic variants, because the associated SNPs may tag nearby functional variants through linkage disequilibrium.

Integration of the GWAS results with trait-specific genetic differentiation and nucleotide diversity further prioritized 55 SNPs for MeL and 62 for MeC. The three SNPs shared between the two traits were also located within candidate differentiated regions for both traits, highlighting a common interval on chromosome 4. Combining association evidence with genomic differentiation therefore helped narrow the initial GWAS signals and identify loci supported by complementary analytical evidence from the same experimental population. Because the contrasting phenotypic groups were derived from the same experimental population, however, the detected differentiation reflects genomic differences between individuals with divergent phenotypes rather than evidence of historical selection, and functional validation is still required to establish causal relationships. It should be noted that genetic differentiation between extreme phenotype groups does not by itself demonstrate a causal or functional relationship with metatarsal traits. Such differentiation may also arise from population structure, genetic drift, or linkage with variants affecting correlated traits. Therefore, the *F_ST_*–*π* results were interpreted as candidate regional signals rather than direct evidence of causal loci. Only regions supported by additional evidence, including GWAS associations, LD structure, and functional annotation, were prioritized as candidate loci for further investigation.

Most of the candidate variants were located in intronic, intergenic, upstream, or downstream regions, consistent with the predominantly regulatory nature of many variants underlying complex quantitative traits [28]. These variants may influence gene expression or regulatory activity rather than directly alter protein sequence, although their functional effects remain to be determined. Among the annotated variants, 4_52426323 was the only variant predicted to have a MODERATE impact and resulted in an amino acid substitution in *ANKRD50*. *ANKRD50* participates in endosomal recycling and membrane-protein trafficking [29], but direct evidence linking this gene to skeletal development in chickens is currently lacking. Thus, the biological significance of this missense variant requires further investigation through expression, cellular, and functional assays.

Several candidate genes within the prioritized regions have plausible biological connections to skeletal or muscular development. *APP* has been shown to promote osteoblast survival and bone formation, suggesting a potential role in maintaining bone homeostasis [30]. *LDB3* encodes a Z-disc-associated protein in striated muscle and contributes to the maintenance of sarcomere integrity and muscle function [31]. Given that metatarsal morphology reflects both skeletal growth and the mechanical environment generated by surrounding musculature, these genes represent biologically plausible candidates for variation in MeL and MeC. However, the available functional evidence is derived largely from mammalian systems, and their specific roles in chicken metatarsal development remain to be established.

The chromosome 4 candidate interval also contained *MTTP*, *ADH6*, *C4H4orf54*, and an unnamed gene. Among these, *MTTP* is involved in lipid transport and has previously been associated with hepatic lipid metabolism and promoter methylation in chickens [32]. This observation is particularly relevant because the population used in the present study originated from broiler lines divergently selected for abdominal fat content. The association of this region with metatarsal traits may therefore reflect biological links between energy metabolism, growth, and skeletal development, but it may also partly reflect the genetic background generated by long-term divergent selection for abdominal fat.

### 4.3. Genomic Prediction Performance and Candidate-Marker Efficiency

Using the common set of 10,305 LD-pruned and position-thinned SNPs, mean prediction accuracies differed only modestly among the six genomic prediction models. BayesA achieved the highest numerical mean accuracy for MeL, whereas GBLUP performed best numerically for MeC. However, the substantial overlap among repeated cross-validation results indicated that no single model consistently outperformed the others. Differences in genomic prediction performance can depend on assumptions regarding marker effects, the underlying genetic architecture of the trait, reference-population size and relatedness, and the extent of LD between markers and causal variants [9,33]. The generally higher prediction accuracies observed for MeC may also be related, at least in part, to its higher genomic heritability.

More pronounced differences were observed when marker composition rather than prediction model was compared. Candidate-union GBLUP and GFBLUP yielded higher apparent within-population heritability-adjusted prediction estimates than full-density GBLUP and matched-random-marker GBLUP for both MeL and MeC. The random marker sets were matched to the candidate sets by marker number, chromosome, and MAF distribution; however, unlike the candidate sets, they were not selected using phenotype-informed analyses of all 330 individuals. Because the random marker sets were matched to the candidate sets with respect to marker number, chromosome, and MAF distribution, their lower predictive performance suggests that the integrated candidate-marker sets captured a greater proportion of trait-associated genetic signal than comparable randomly selected markers. Previous studies have shown that GFBLUP can improve genomic prediction when predefined genomic features contain an increased concentration of variants related to the target trait while reducing noise from less informative markers [25]. Consistent with this principle, the present results indicate that integrating GWAS with *F_ST_*–*π* evidence can identify marker subsets with potential value for genomic prediction. Nevertheless, the performance of GFBLUP also indicates that genome-wide background information remains relevant, and the candidate markers should not be considered a complete substitute for genome-wide markers.

Several limitations should be considered when interpreting these findings. First, the study included 330 broilers and approximately 9.84 million SNPs, and no a priori power analysis was performed. The relatively limited sample size may have reduced the power to detect small-effect loci and increased uncertainty in the estimated association effects. It may also affect the stability and reproducibility of individual associations, particularly for suggestive loci detected in the univariate GWAS analyses. Therefore, the 102 SNPs identified for MeL and the 165 SNPs identified for MeC should be interpreted as candidate associations rather than definitive causal loci. However, integration with bivariate GWASs, genetic differentiation analyses, LD-based refinement, and functional annotation provided additional evidence for prioritizing a subset of these candidate loci. Second, the candidate-marker sets were selected using the full dataset before cross-validation, meaning that phenotypic information from individuals subsequently assigned to validation folds indirectly contributed to marker selection. This design may have inflated the apparent predictive performance of candidate-union GBLUP and GFBLUP [34]. Therefore, the candidate-marker results should be interpreted primarily as evidence of within-population enrichment of trait-relevant information rather than as unbiased estimates of prediction accuracy in independent populations. In addition, because all individuals originated from a single experimental population, the transferability of the identified loci and markers to other broiler populations remains uncertain. Larger sample sizes, nested cross-validation, subsequent generations, and independent populations will be required to validate the robustness and generalizability of these findings.

## 5. Conclusions

This study provides new genomic evidence for understanding the genetic basis of metatarsal morphology in broilers. By integrating single-trait and bivariate GWASs, *F_ST_*–*π* analysis, LD-based refinement, and functional annotation, this study identified 55 candidate SNPs for MeL and 62 for MeC, including three loci shared between the two traits, as well as several biologically plausible candidate genes. Exploratory within-population analyses further suggested that the integrated candidate-marker sets were enriched for trait-associated information. These findings improve our understanding of the genetic architecture underlying MeL and MeC and provide candidate loci and markers for further functional validation and genomic improvement of broiler leg traits. Further validation in larger and independent populations will be necessary to confirm the robustness and practical value of these candidate loci and markers.

## Figures and Tables

**Table 1:** Descriptive statistics and genomic heritability estimates for MeL and MeC.

Trait (Unit)	Number (*n*)	Max	Min	Mean ± SE	Median	SD	CV	*h*^2^ (SE)
MeL (mm)	330	97.48	69.00	85.46 ± 0.27	85.78	4.97	5.82%	0.266 (0.188)
MeC (cm)	330	6.00	4.25	4.91 ± 0.02	4.90	0.31	6.28%	0.516 (0.177)

Note: MeL, metatarsal length; MeC, metatarsal circumference; SE, standard error; SD, standard deviation; CV, coefficient of variation; *h*^2^, genomic heritability.

**Table 2:** Summary of SNPs prioritized by integrating GWAS and *F_ST_*–*π* analyses.

Trait	Single-Trait GWAS	Bivariate GWAS	GWAS Union	SNPs in *F_ST_–π* Regions	Candidate SNPs
MeL	102	263	324	315,202	55
MeC	165	263	365	342,143	62

Note: All values represent numbers of SNPs. The GWAS union was obtained by merging the suggestively associated SNPs from the corresponding single-trait GWAS and the MeL–MeC bivariate GWAS and removing duplicate loci. SNPs in *F_ST_*–*π* regions represent nonredundant quality-controlled SNPs located within the candidate regions for the corresponding trait. Jointly prioritized SNPs were autosomal SNPs present in both the GWAS union and the corresponding *F_ST_*–*π* candidate regions.

**Table 3:** Functional annotations of representative candidate SNPs.

Trait	SNP	Minimum *p*-Value	Functional Annotation	Candidate Gene/Region
MeC	11_7373893	4.909 × 10^−8^	3′UTR variant	*CDCA9*, *DNAJA2*
MeC	4_52499329	1.219 × 10^−7^	Intergenic variant	Intergenic region
MeC	6_3146546	2.127 × 10^−7^	Intronic variant	*LDB3*
MeC	4_51317483	4.496 × 10^−7^	Intergenic variant	Intergenic region
MeC	4_52438421	7.490 × 10^−6^	Splice-region/non-coding transcript exon variant	*ENSGALG00010005750*
MeC	4_52426323	7.739 × 10^−6^	Missense variant	*ANKRD50*
MeC/MeL	4_59544384	8.836 × 10^−6^	3′UTR variant	*C4H4orf54, MTTP*
MeC/MeL	4_59470884	8.897 × 10^−6^	Intergenic variant	*METAP1-ADH6*
MeC/MeL	4_59483846	9.993 × 10^−6^	Synonymous variant	*ADH6*
MeL	4_8841620	6.488 × 10^−8^	Intronic variant	*ENSGALG00010019979*
MeL	1_102807177	4.908 × 10^−7^	Intronic variant	*APP*

## Data Availability

All data is included in this paper.

## Supplementary Materials

The following supporting information can be downloaded at: https://www.mdpi.com/article/10.3390/ani16203189/s1, Figure S1: Principal component analysis of population structure in the two broiler lines; Figure S2: LD decay diagram; Figure S3: Regional association and linkage disequilibrium plot for the MeL chromosome Z signal; Figure S4: Local association and linkage disequilibrium plots for MeL autosomal signals; Figure S5: Local association and linkage disequilibrium plots for MeC autosomal signals; Figure S6: Predictive ability, phenotypic prediction correlation, and root mean square error for the six genomic prediction models; Figure S7: Error and calibration metrics for the six genomic prediction models; Figure S8: Predictive ability, phenotypic prediction correlation, and root mean square error for the marker-set comparisons; Figure S9: Error and calibration metrics for the marker-set comparisons; Table S1: Complete integrated candidate-locus, variant, and gene results; Table S2: Complete genomic prediction results; Table S3: Bioinformatics and statistical software used in this study, including versions, websites/URLs; Table S4: Retention of prioritized SNPs across alternative cutoffs.
