# Supplementary material for: Integrative Genomic Analyses Identify and Prioritize Candidate Loci for Metatarsal Length and Circumference in Broilers
Source: Animals (Basel). 2026 Oct 11;16(20):3189. doi: 10.3390/ani16203189 (PMC13700583; doi:10.3390/ani16203189)
Supplement: Supplementary file 1 [file animals-16-03189-s001.zip › animals-4580849-supplementary.pdf]

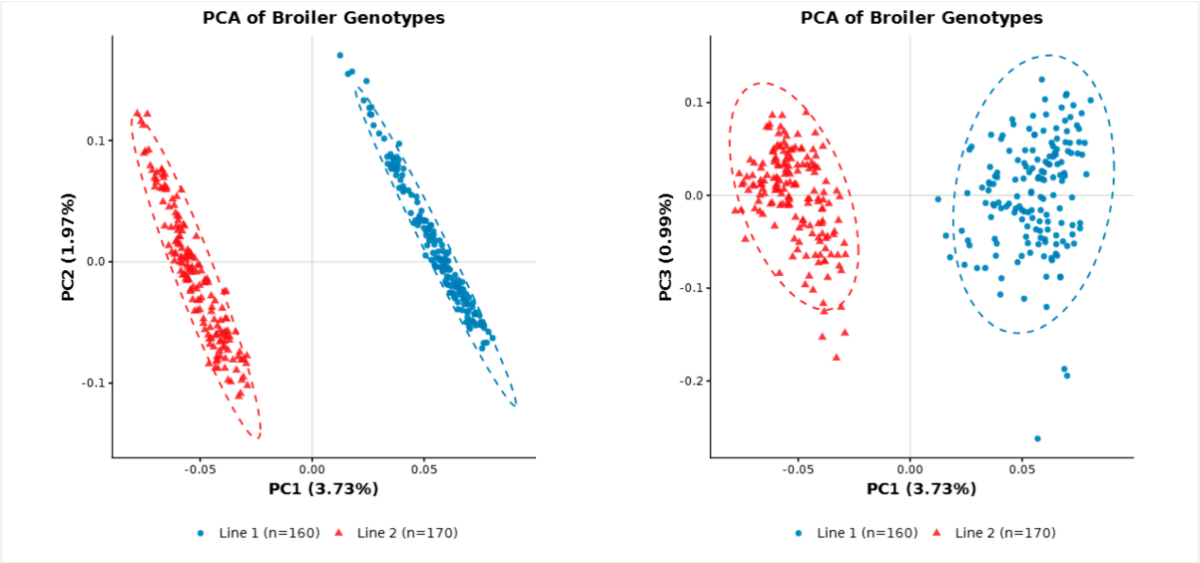

Figure S1. Principal component analysis of population structure in the two broiler lines.

Note: Principal component analysis was performed for 330 broilers using 440,775 LD-pruned SNPs located on autosomes 1–39. (A) Scatter plot of PC1 and PC2; (B) scatter plot of PC1 and PC3. Each point represents one bird; blue circles indicate the high-fat line, and red triangles indicate the low-fat line. Dashed ellipses indicate the 95% normal-probability regions for the corresponding lines. PC1, PC2, and PC3 explained 3.73%, 1.97%, and 0.99% of the total genotypic variance, respectively.

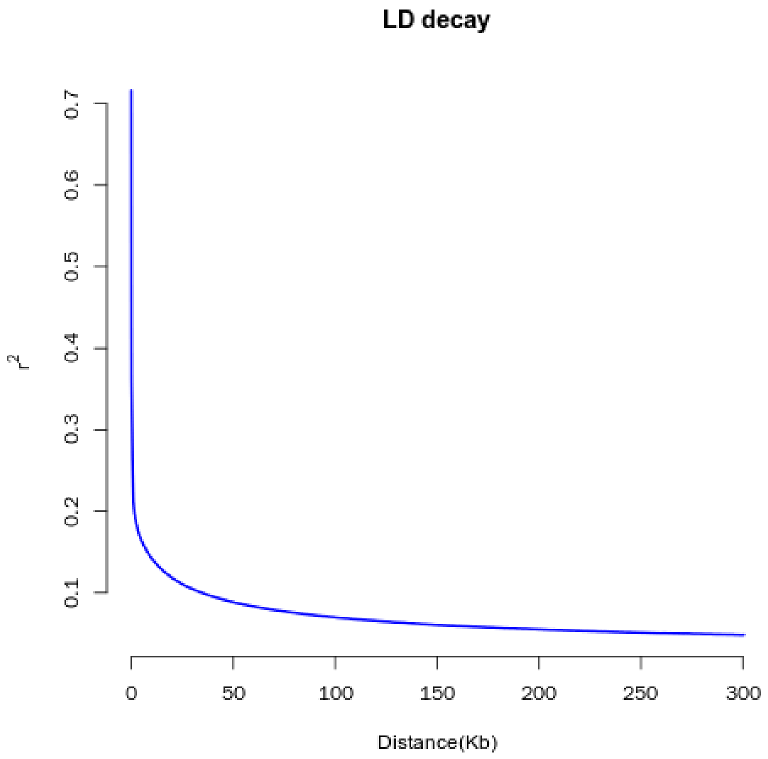

Figure S2. LD decay diagram

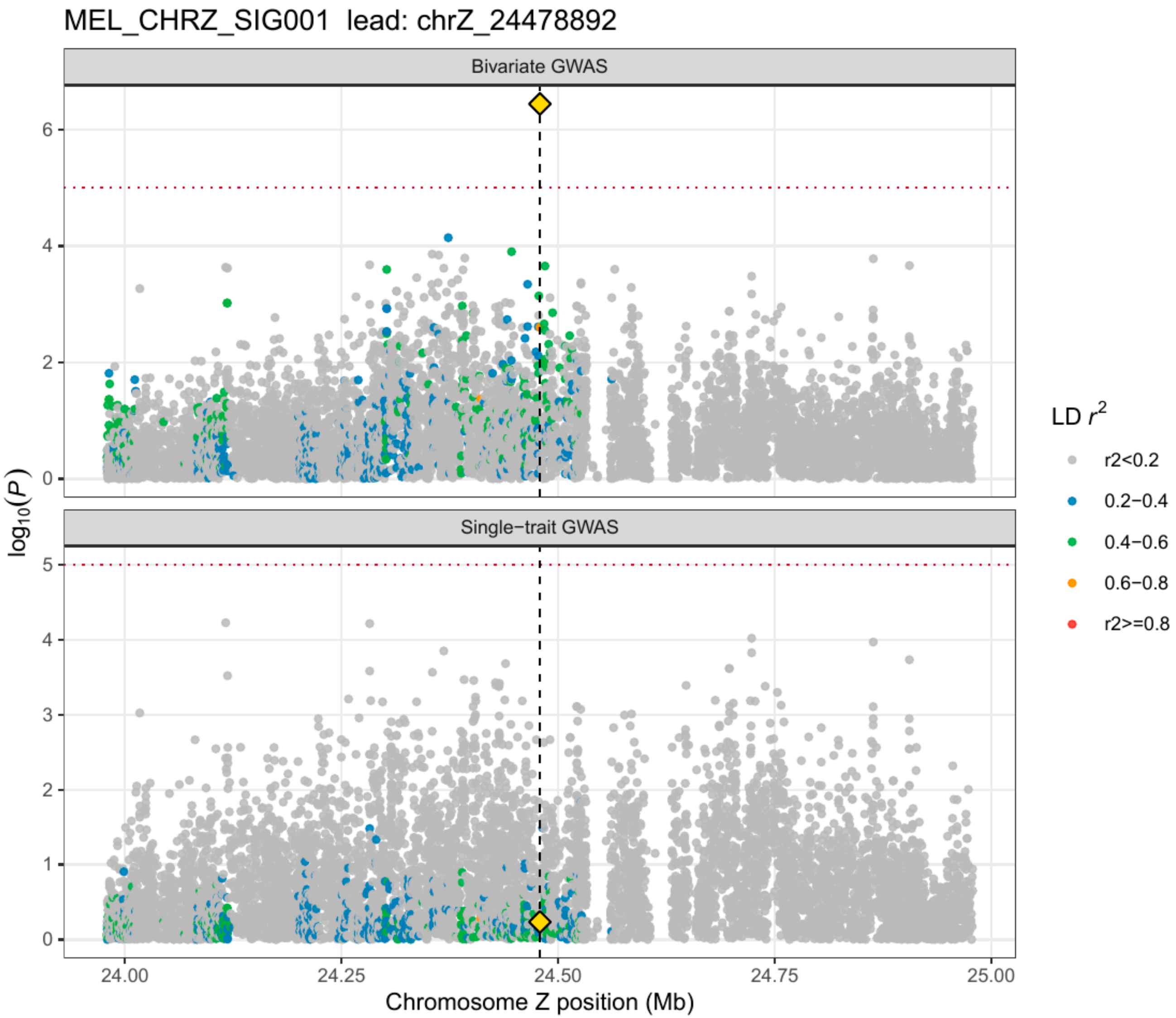

Figure S3. Regional association and linkage disequilibrium plot for the MeL chromosome Z signal.

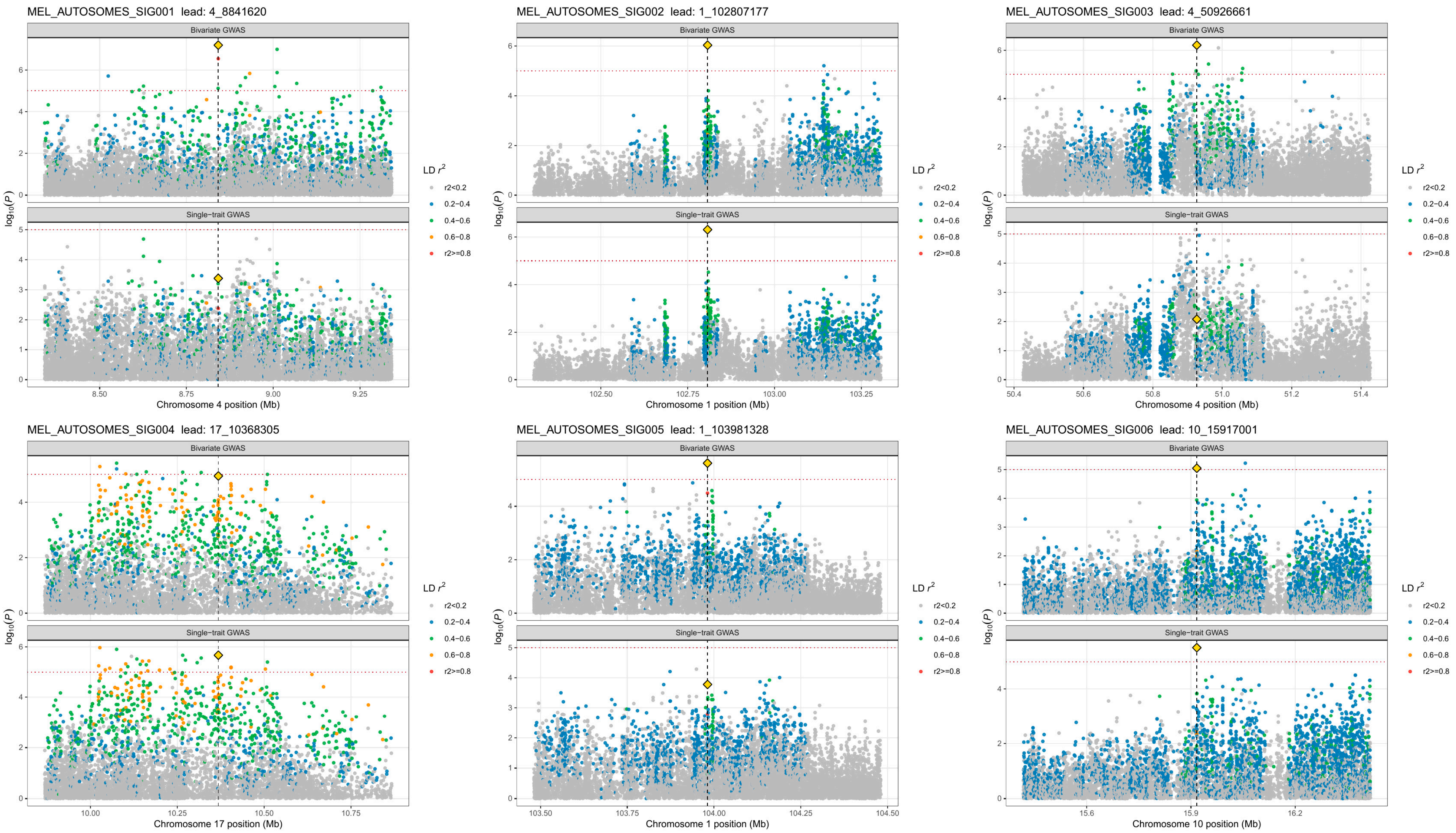

Figure S4. Local association and linkage disequilibrium plots for MeL autosomal signals.

(To be continued on next page)

(Figure S4 continued from previous page)

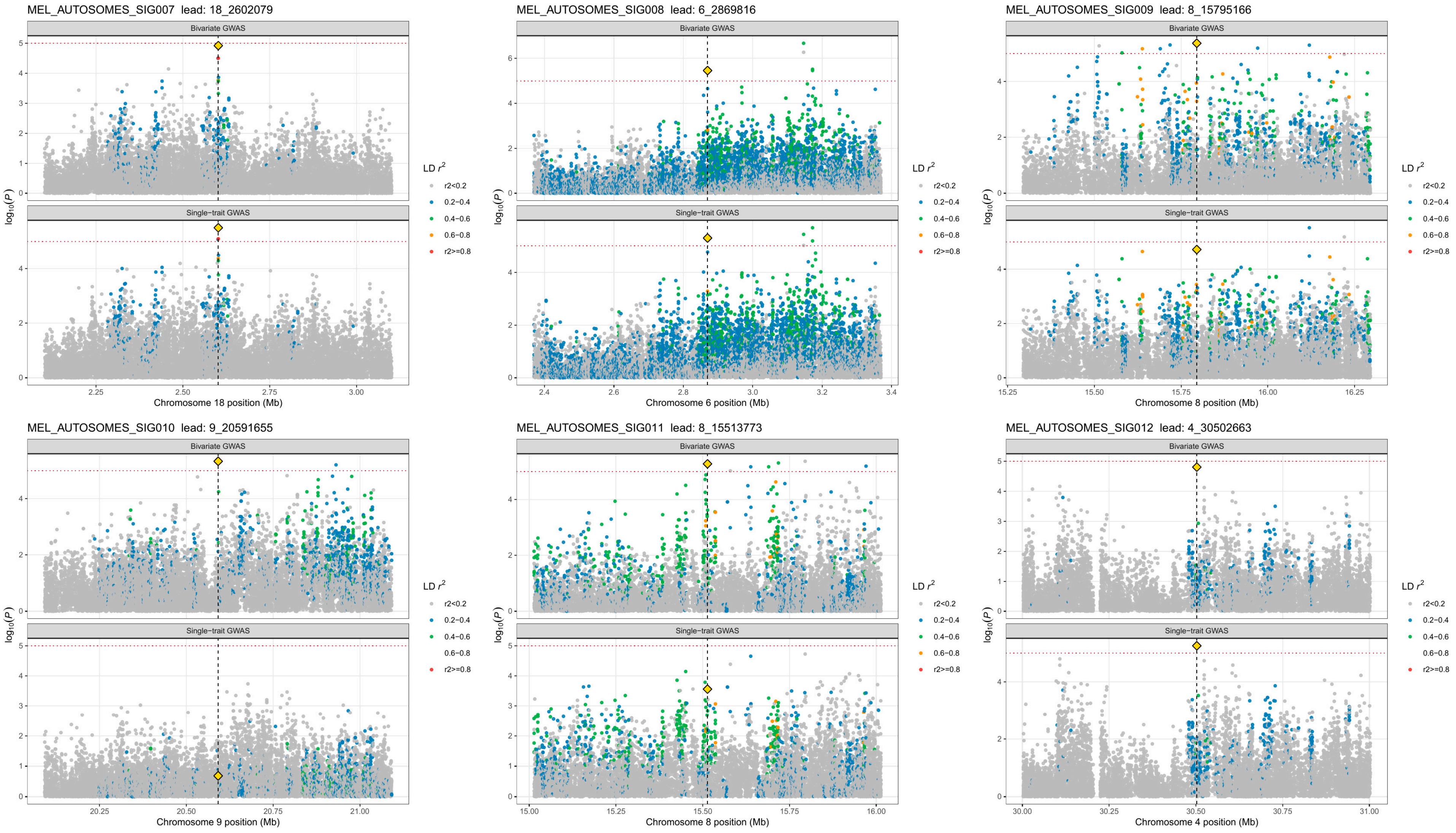

Figure S4 (continued).

(To be continued on next page)

(Figure S4 continued from previous page)

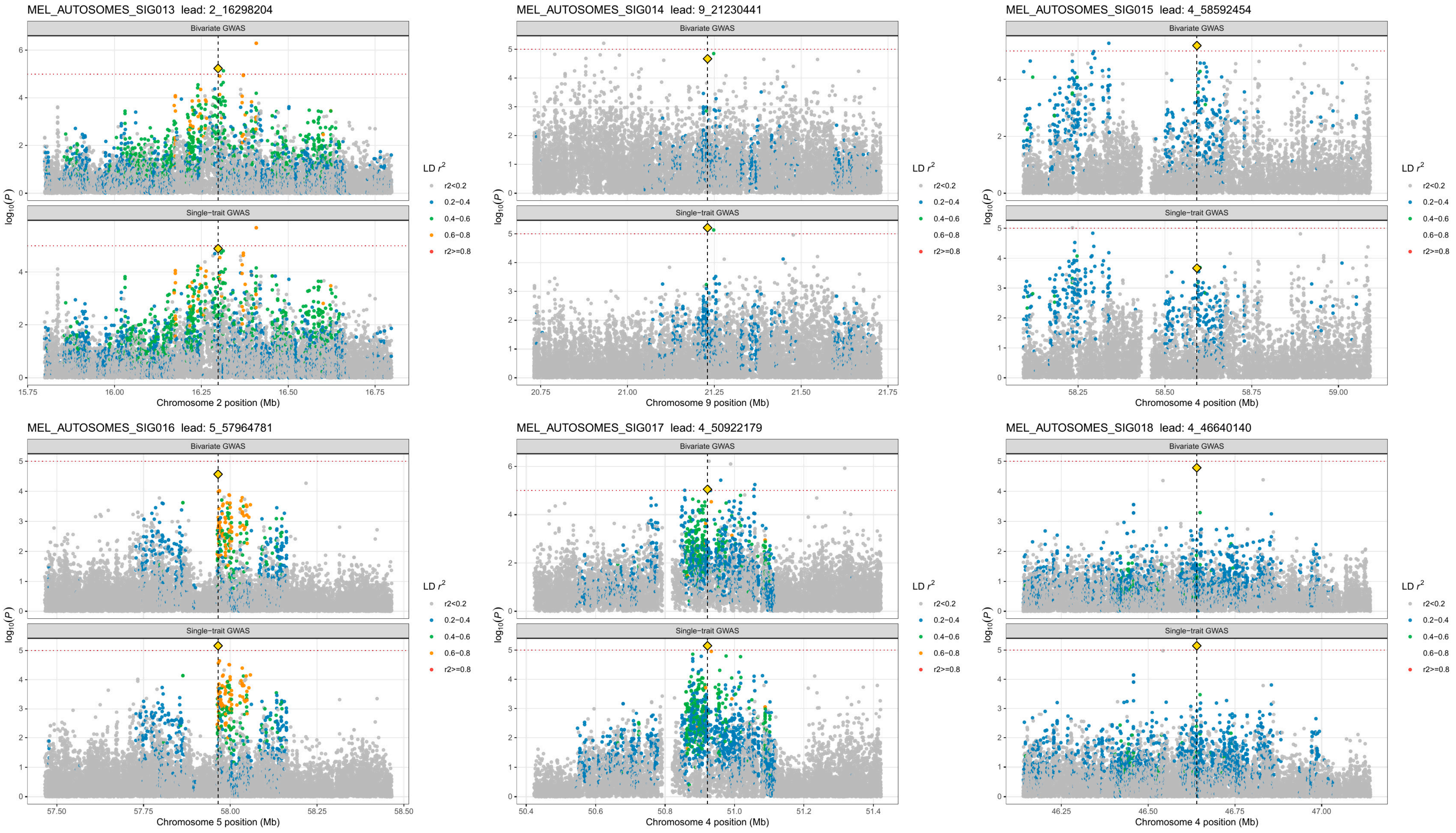

Figure S4 (continued).

(To be continued on next page)

(Figure S4 continued from previous page)

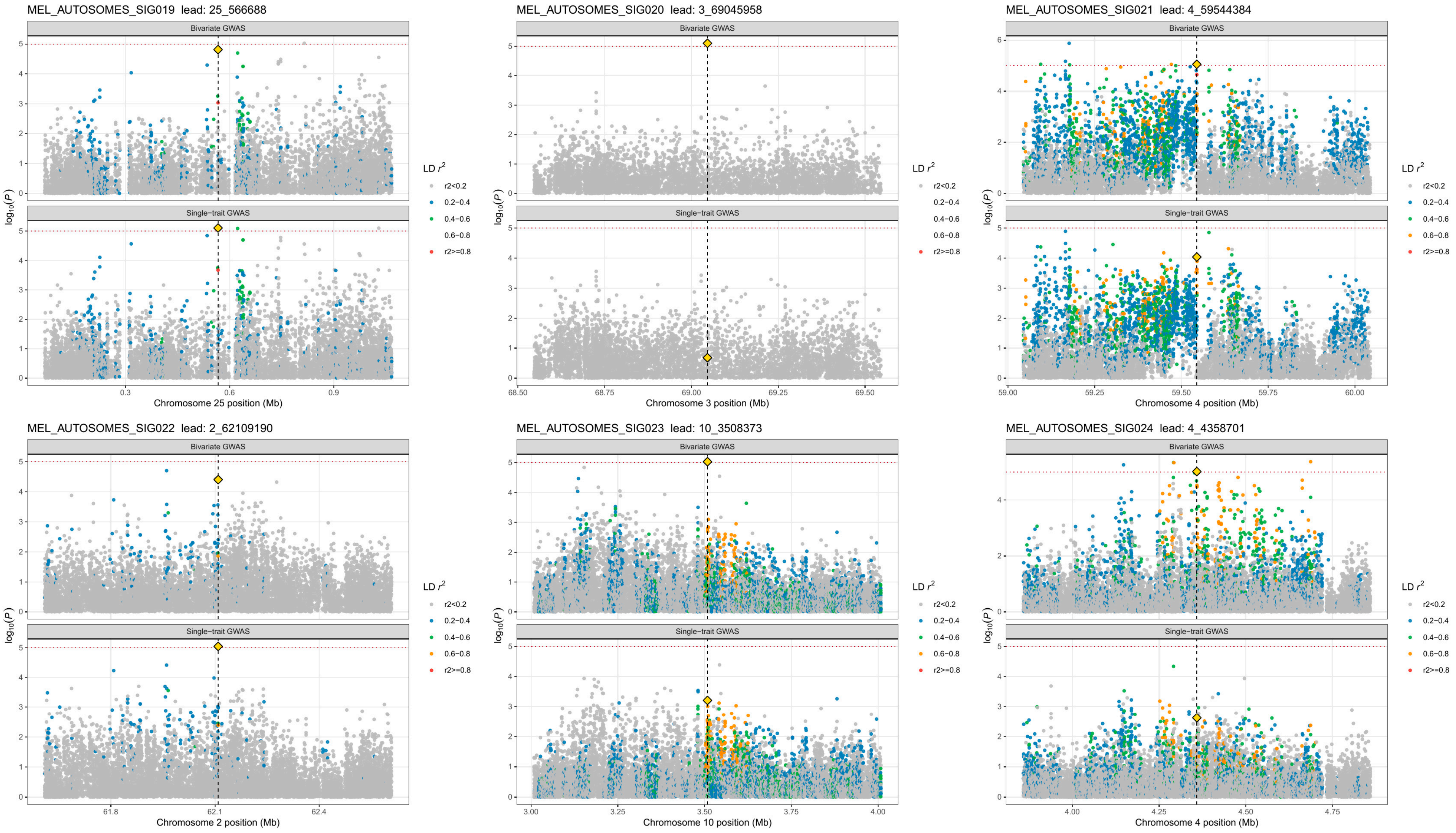

Figure S4 (continued).

(To be continued on next page)

(Figure S4 continued from previous page)

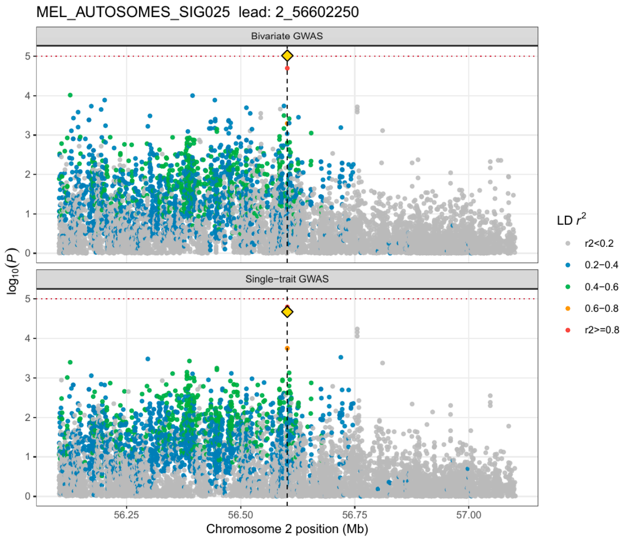

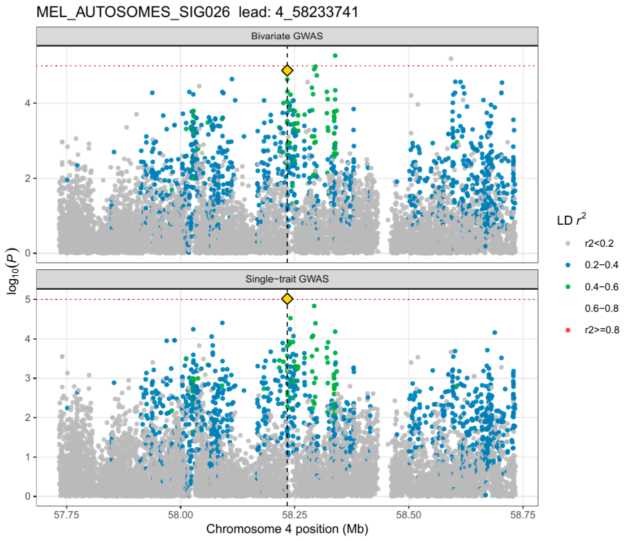

Figure S4 (continued).

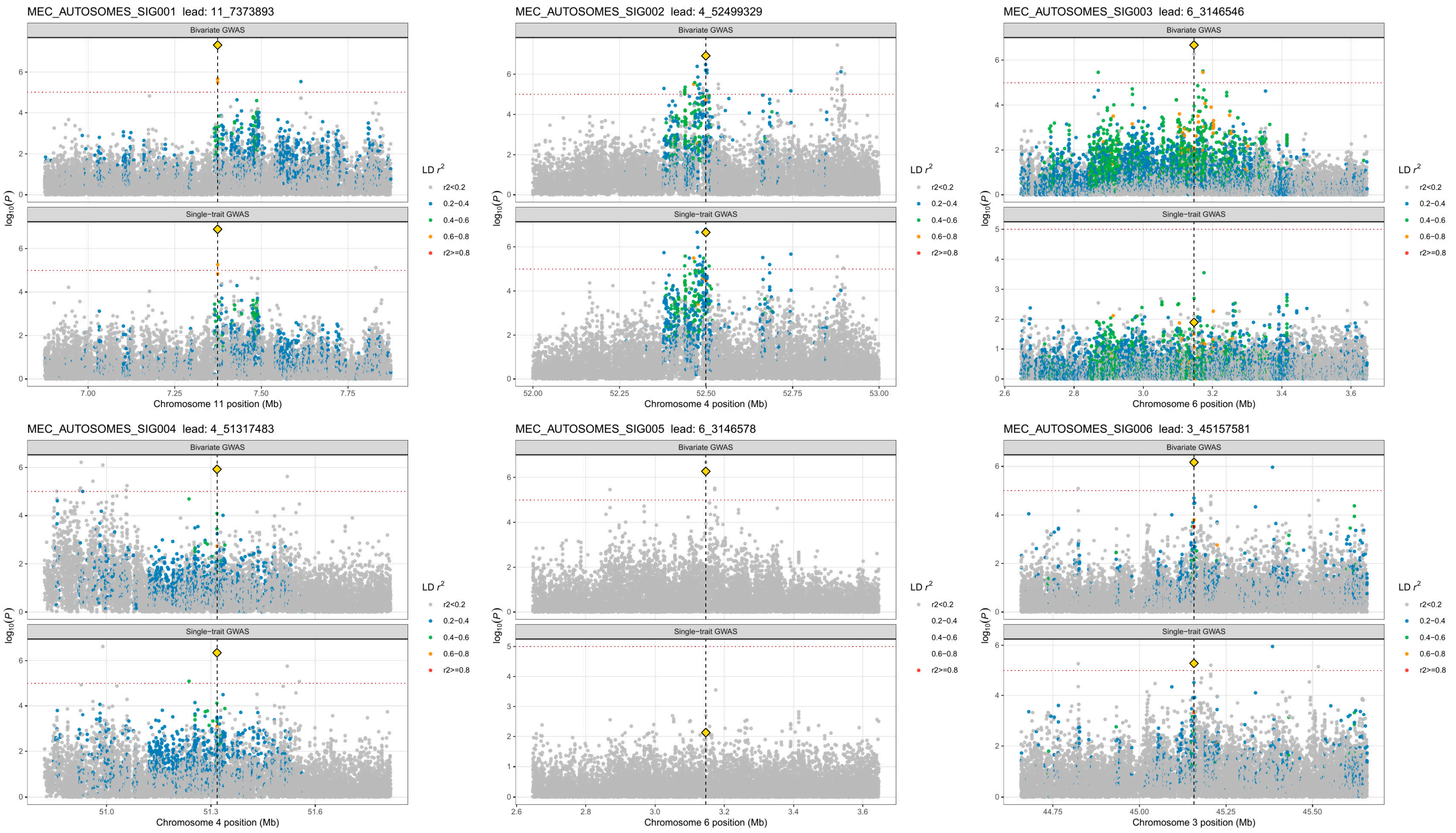

Figure S5. Local association and linkage disequilibrium plots for MeC autosomal signals.

(To be continued on next page)

(Figure S5 continued from previous page)

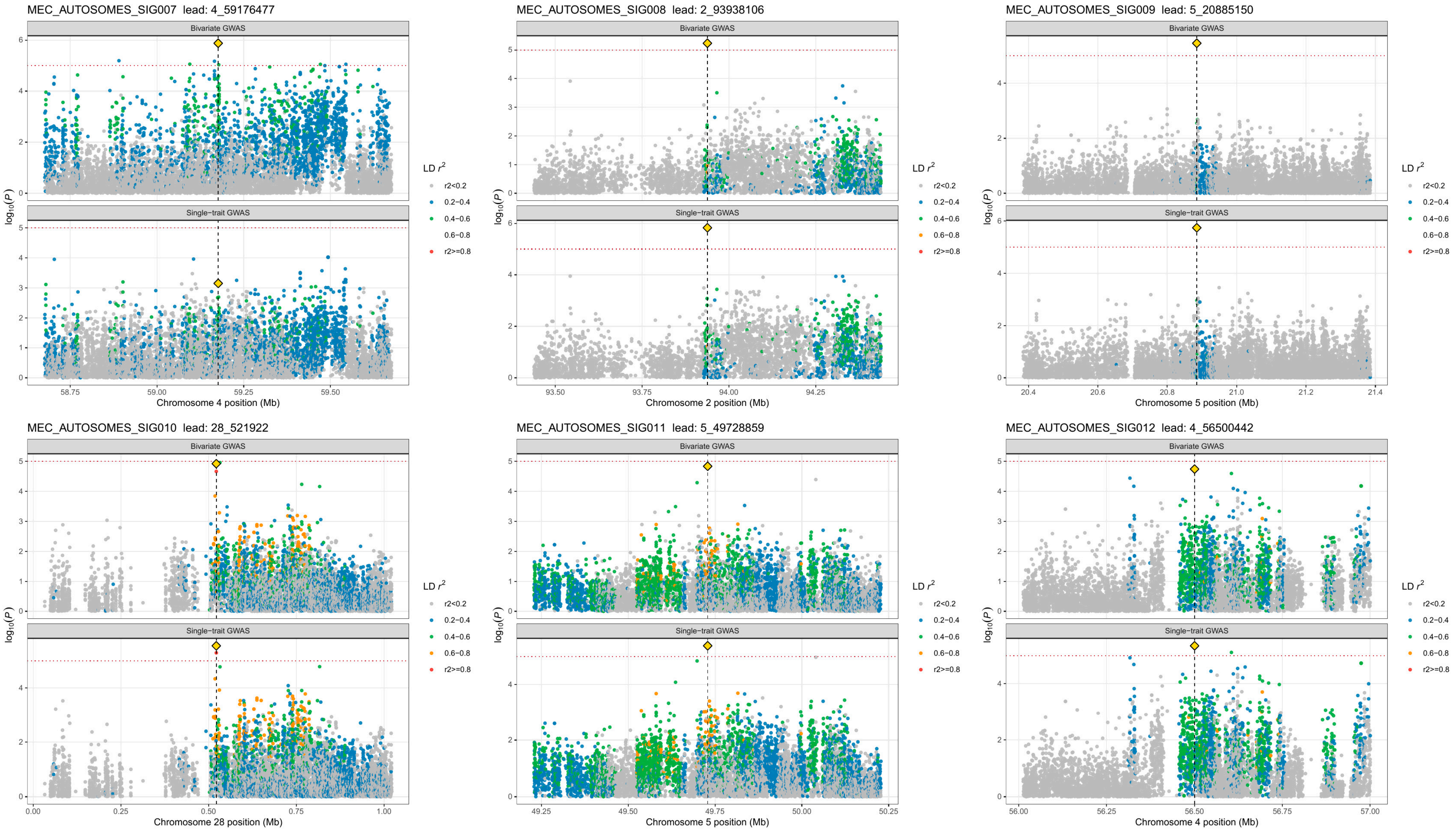

Figure S5 (continued).

(To be continued on next page)

(Figure S5 continued from previous page)

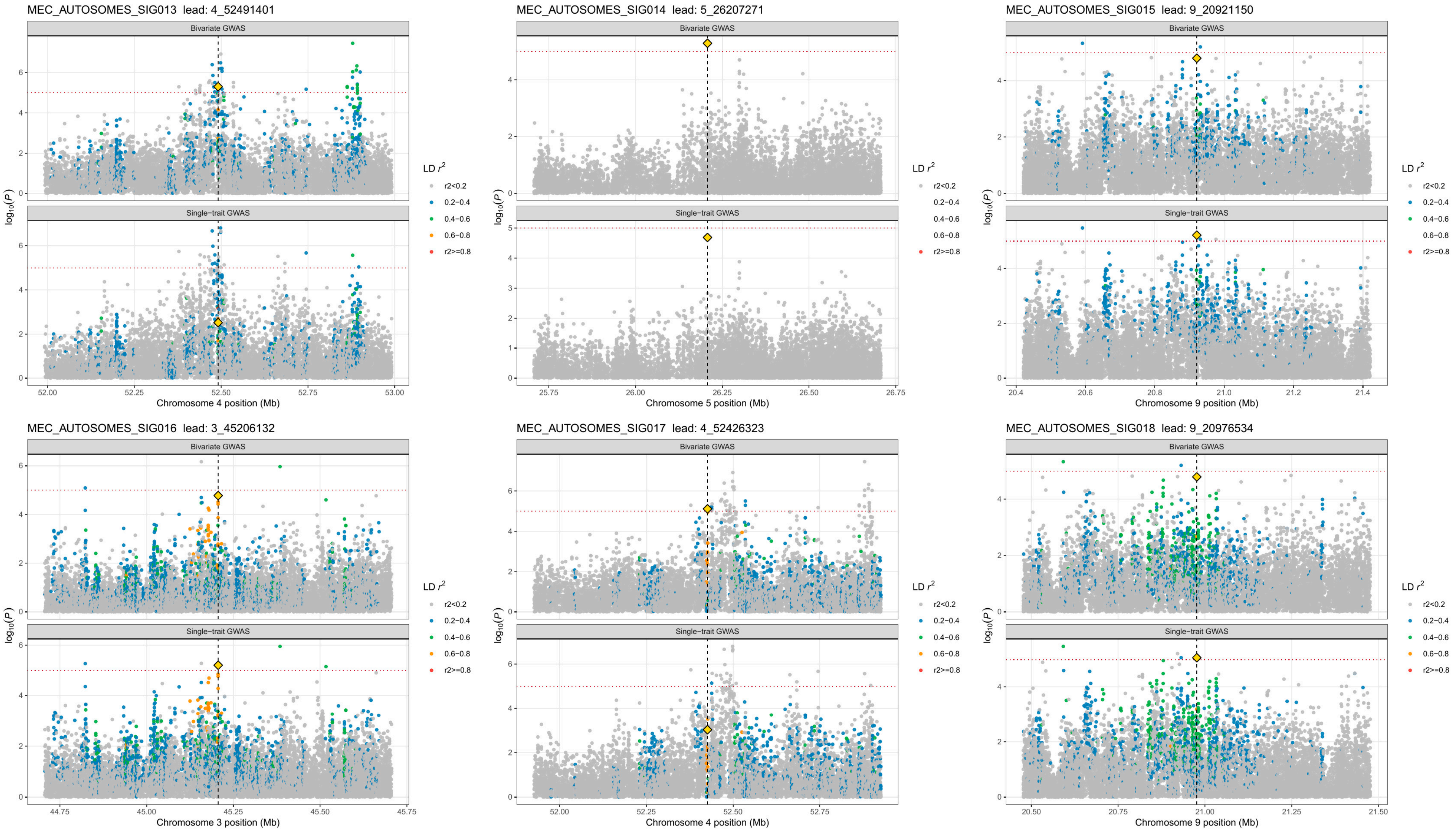

Figure S5 (continued).

(To be continued on next page)

(Figure S5 continued from previous page)

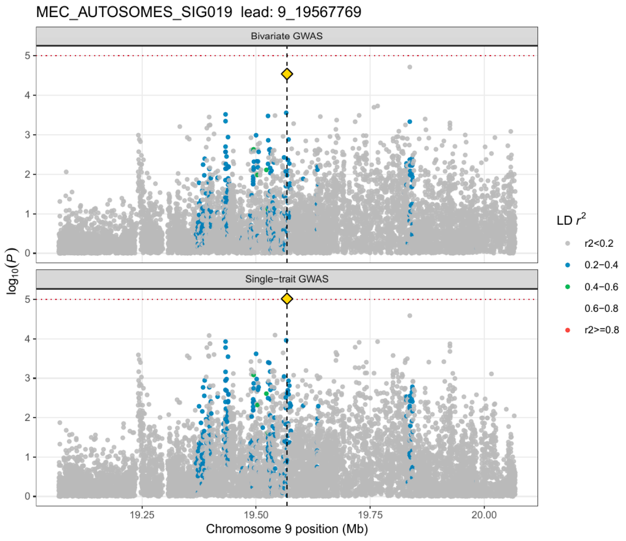

Figure S5 (continued).

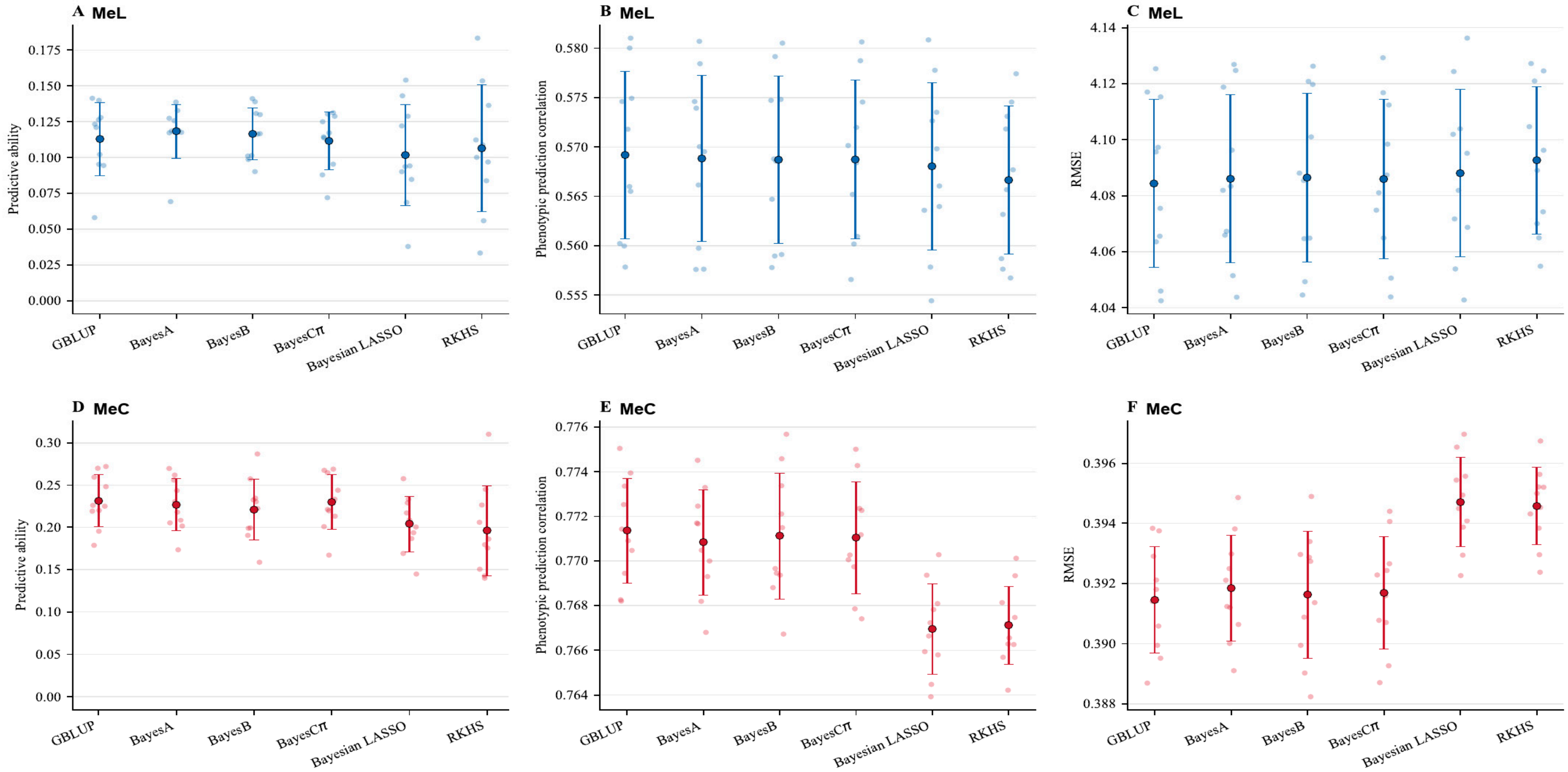

Figure S6. Predictive ability, phenotypic prediction correlation, and root mean square error for the six genomic prediction models.

Note: Small points represent the results from the 10 repeated fivefold cross-validation runs. Solid points represent the mean across the 10 repetitions, and error bars represent the standard deviation (SD).

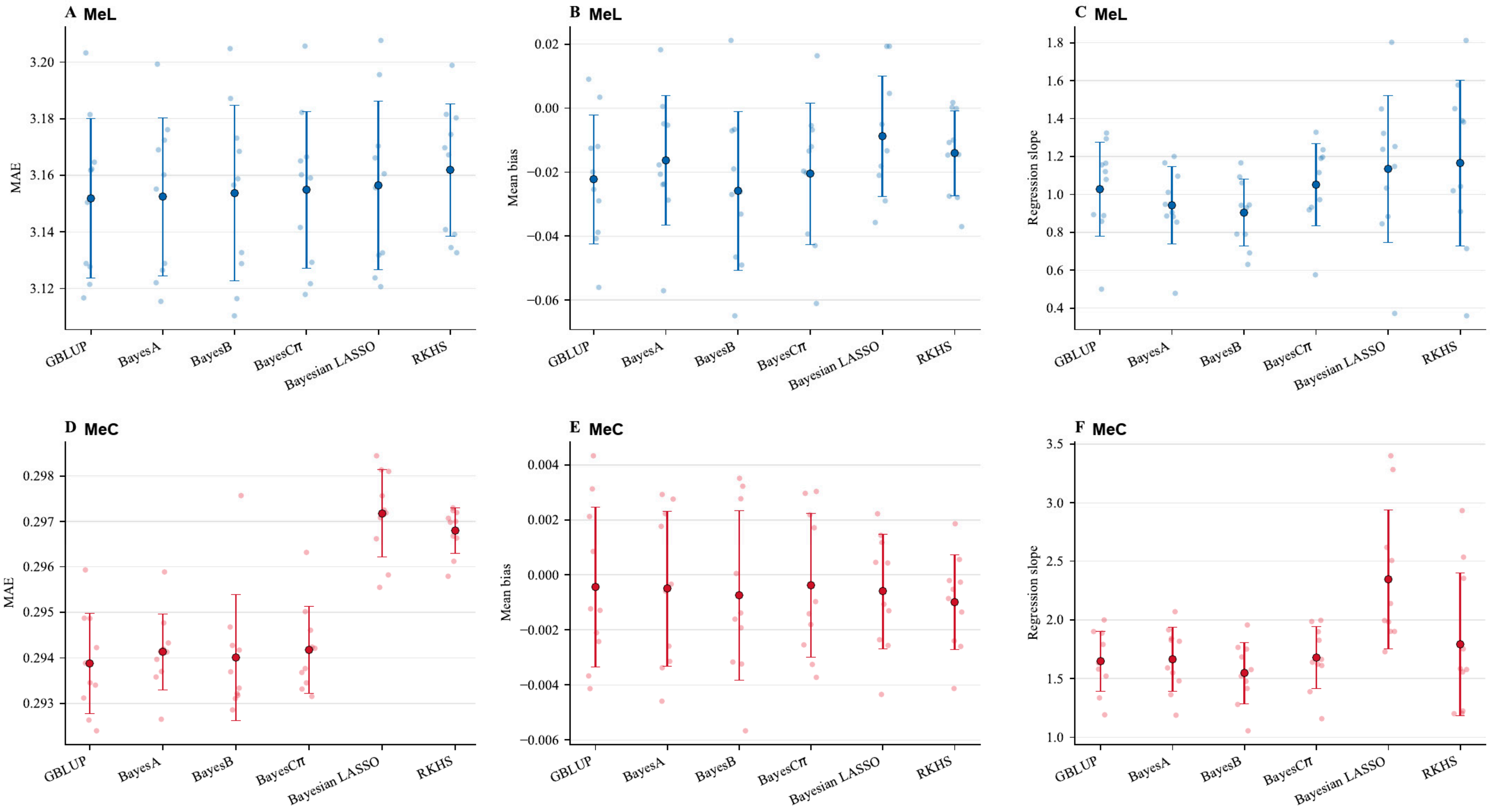

Figure S7. Error and calibration metrics for the six genomic prediction models.
Note: Small points represent the results from the 10 repeated fivefold cross-validation runs. Solid points represent the mean across the 10 repetitions, and error bars represent the standard deviation (SD).

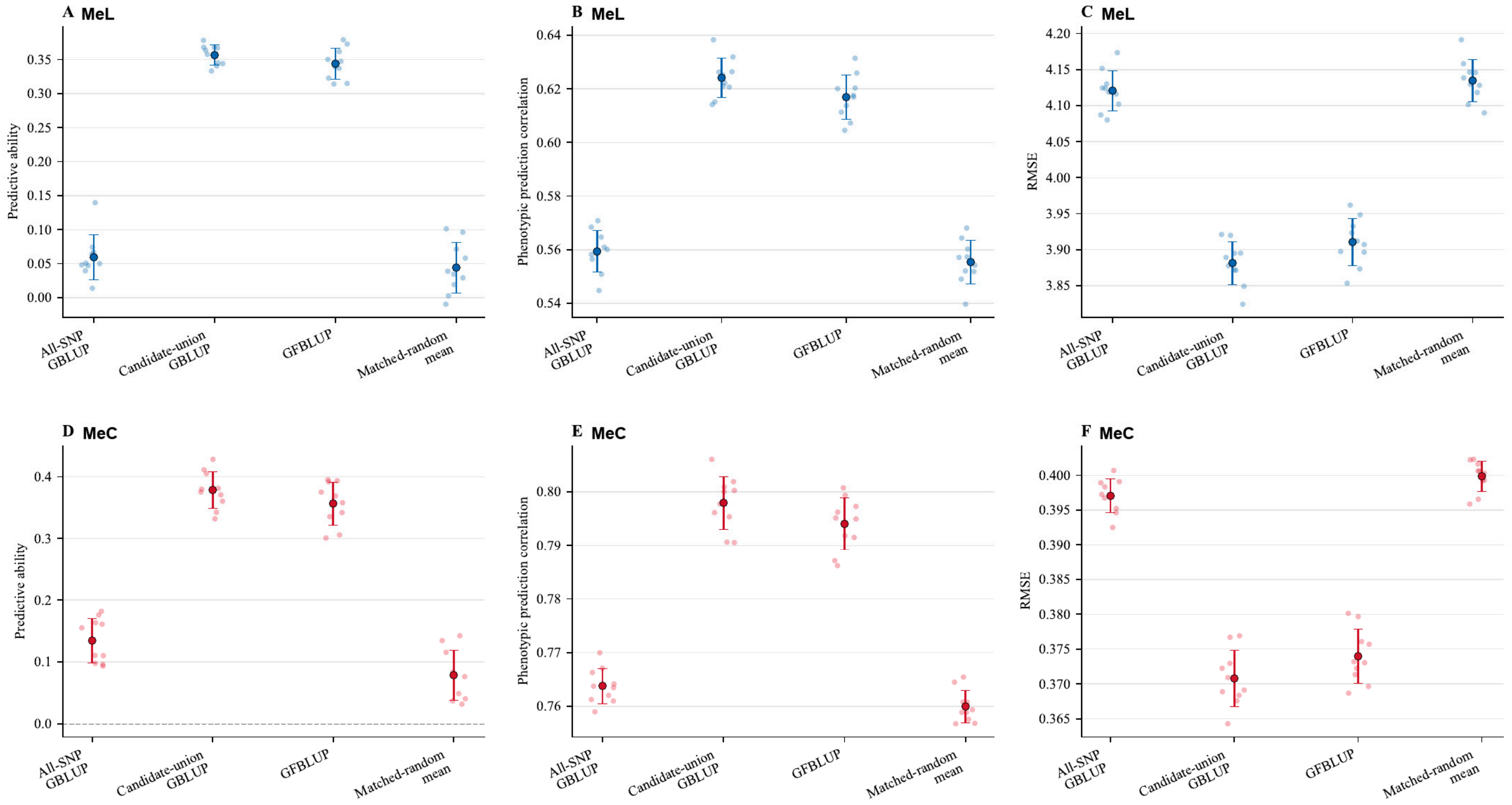

Figure S8. Predictive ability, phenotypic prediction correlation, and root mean square error for the marker-set comparisons.

Note: Small points represent the results from the 10 repeated fivefold cross-validation runs. Solid points represent the mean across the 10 repetitions, and error bars represent the standard deviation (SD).

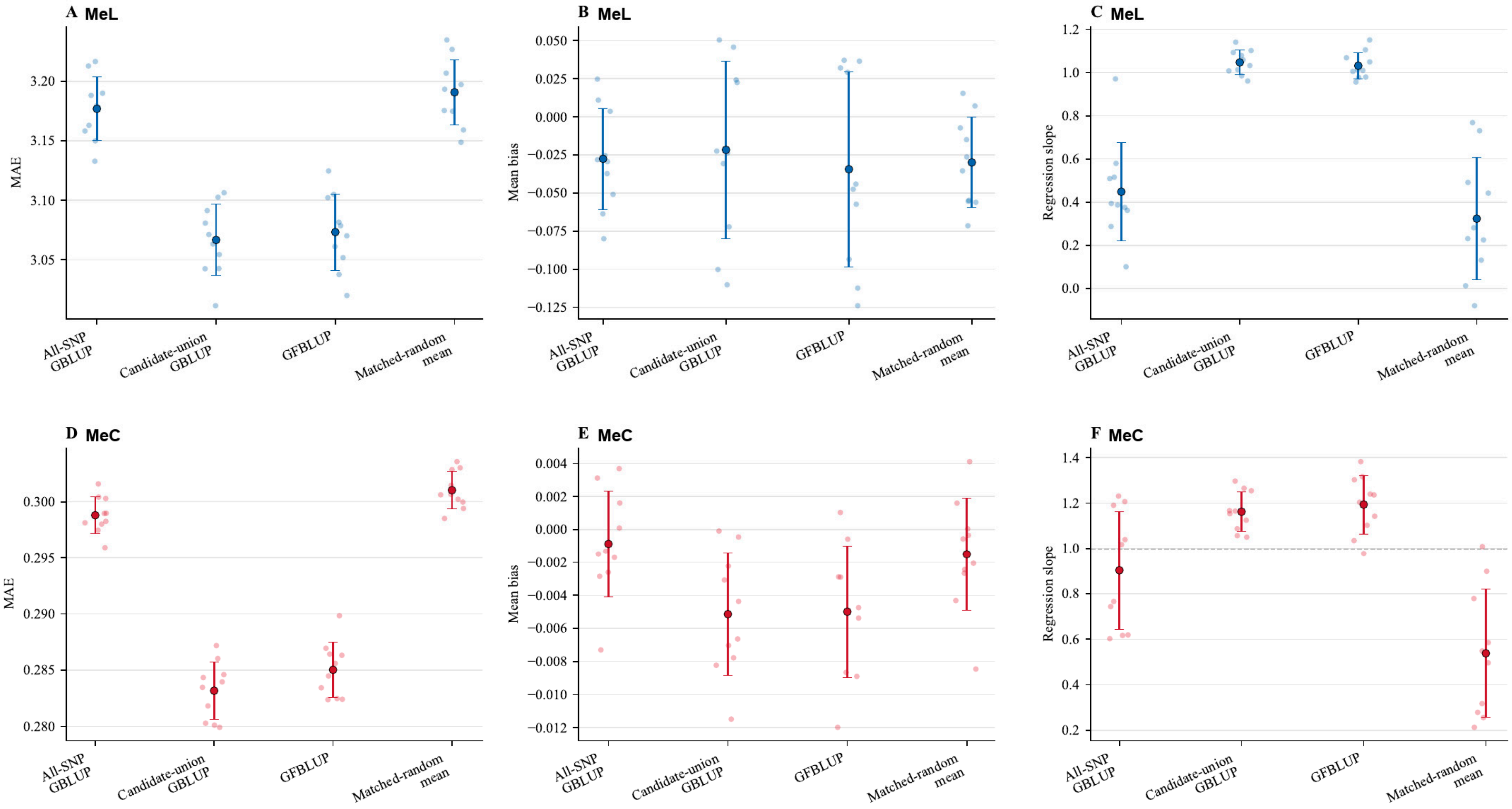

Figure S9. Error and calibration metrics for the marker-set comparisons.

Note: Small points represent the results from the 10 repeated fivefold cross-validation runs. Solid points represent the mean across the 10 repetitions, and error bars represent the standard deviation (SD).

Table S1. Part A. Overview of integrated candidate-marker results.

Trait	Single-trait GWAS SNPs	Bivariate GWAS SNPs	GWAS-union SNPs	Priority SNPs (autosomes)	Initial regions	LD-refined signals	Candidate genes (autosomes)
MeL	102	263	324	55	20	26	37
MeC	165	263	365	62	14	19	30

Table S1 (continued). Part B. Representative priority variants and their supporting evidence.

Trait	SNP	Best P	GWAS evidence	LD signal	Functional annotation	Candidate gene(s)	Selection rationale
MeC	11_7373893	4.909e-08	Single-trait + bivariate	MEC_AUTOSOMES_SIG001	3′ UTR variant	CDCA9, DNAJA2	lead $P<5\times10^{-7}$
MeC	4_52499329	1.219e-07	Single-trait + bivariate	MEC_AUTOSOMES_SIG002	Intergenic region	Unnamed/intergenic region	lead $P<5\times10^{-7}$
MeC	6_3146546	2.127e-07	Bivariate only	MEC_AUTOSOMES_SIG003	Intron variant	LDB3	lead $P<5\times10^{-7}$
MeC	4_51317483	4.496e-07	Single-trait + bivariate	MEC_AUTOSOMES_SIG004	Intergenic region	Unnamed/intergenic region	lead $P<5\times10^{-7}$
MeC	4_52438421	7.490e-06	Bivariate only	MEC_AUTOSOMES_SIG002	Splice-region/non-coding exon variant	Unnamed/intergenic region	LOW impact
MeC	4_52426323	7.739e-06	Bivariate only	MEC_AUTOSOMES_SIG017	Missense variant	ANKRD50	MODERATE impact
MeC/MeL	4_59544384	8.836e-06	Bivariate only	MEC_AUTOSOMES_SIG007; MEL_AUTOSOMES_SIG021	3′ UTR variant	C4H4orf54, MTTP	Shared by MeL and MeC
MeC/MeL	4_59470884	8.897e-06	Bivariate only	MEC_AUTOSOMES_SIG007; MEL_AUTOSOMES_SIG021	Intergenic region	METAP1-ADH6	Shared by MeL and MeC
MeC/MeL	4_59483846	9.993e-06	Bivariate only	MEC_AUTOSOMES_SIG007; MEL_AUTOSOMES_SIG021	Synonymous variant	ADH6	Shared by MeL and MeC; LOW impact
MeL	4_8841620	6.488e-08	Bivariate only	MEL_AUTOSOMES_SIG001	Intron variant	Unnamed/intergenic region	lead $P<5\times10^{-7}$
MeL	1_102807177	4.908e-07	Single-trait + bivariate	MEL_AUTOSOMES_SIG002	Intron variant	APP	lead $P<5\times10^{-7}$

Table S1 (continued). Part C1. MeL prioritized candidate SNPs: association statistics.

TRAIT	SCOPE	CHR	SNP	BP	ALLELE1	ALLELE0	AF	GWAS SOURCE	P SINGLE	P JOINT	BEST P
MeL	autosomes	1	1_102807177	102807177	T	C	0.244	both	4.907622e-07	9.206823e-07	4.907622e-07
MeL	autosomes	1	1_103142183	103142183	T	C	0.176	bivariate_only		6.206678e-06	6.206678e-06
MeL	autosomes	1	1_103981328	103981328	A	C	0.444	bivariate_only		2.430544e-06	2.430544e-06
MeL	autosomes	2	2_16298204	16298204	A	G	0.159	bivariate_only		5.831447e-06	5.831447e-06
MeL	autosomes	2	2_16313576	16313576	C	T	0.138	bivariate_only		7.350077e-06	7.350077e-06
MeL	autosomes	2	2_56602250	56602250	A	T	0.117	bivariate_only		9.610784e-06	9.610784e-06
MeL	autosomes	2	2_56602264	56602264	C	A	0.117	bivariate_only		9.610784e-06	9.610784e-06
MeL	autosomes	2	2_62109190	62109190	T	C	0.071	single_only	9.119914e-06		9.119914e-06
MeL	autosomes	3	3_69045958	69045958	G	C	0.059	bivariate_only		7.951757e-06	7.951757e-06
MeL	autosomes	4	4_4358701	4358701	A	G	0.123	bivariate_only		9.529743e-06	9.529743e-06

TRAIT	SCOPE	CHR	SNP	BP	ALLELE1	ALLELE0	AF	GWAS SOURCE	P SINGLE	P JOINT	BEST P
MeL	autosomes	4	4_8841206	8841206	A	G	0.064	bivariate_only		7.59691e-06	7.59691e-06
MeL	autosomes	4	4_8841620	8841620	C	T	0.059	bivariate_only		6.48835e-08	6.48835e-08
MeL	autosomes	4	4_8841631	8841631	C	A	0.055	bivariate_only		2.892894e-07	2.892894e-07
MeL	autosomes	4	4_8905235	8905235	C	T	0.062	bivariate_only		5.765117e-06	5.765117e-06
MeL	autosomes	4	4_8920040	8920040	G	A	0.073	bivariate_only		2.316085e-06	2.316085e-06
MeL	autosomes	4	4_8932225	8932225	G	C	0.073	bivariate_only		1.471114e-06	1.471114e-06
MeL	autosomes	4	4_9004661	9004661	C	T	0.059	bivariate_only		6.207736e-06	6.207736e-06
MeL	autosomes	4	4_30502663	30502663	A	G	0.324	single_only	5.464323e-06		5.464323e-06
MeL	autosomes	4	4_46640140	46640140	C	T	0.276	single_only	7.094158e-06		7.094158e-06
MeL	autosomes	4	4_50856699	50856699	T	C	0.465	bivariate_only		9.754558e-06	9.754558e-06
MeL	autosomes	4	4_50922179	50922179	G	C	0.386	both	7.016125e-06	8.928057e-06	7.016125e-06
MeL	autosomes	4	4_50924668	50924668	C	T	0.309	bivariate_only		7.119445e-06	7.119445e-06
MeL	autosomes	4	4_50926661	50926661	G	A	0.452	bivariate_only		6.15706e-07	6.15706e-07
MeL	autosomes	4	4_50931021	50931021	A	T	0.364	bivariate_only		9.903117e-06	9.903117e-06
MeL	autosomes	4	4_50960354	50960354	A	G	0.327	bivariate_only		3.740098e-06	3.740098e-06
MeL	autosomes	4	4_58233741	58233741	T	C	0.203	single_only	9.628314e-06		9.628314e-06
MeL	autosomes	4	4_58592454	58592454	G	A	0.058	bivariate_only		6.448875e-06	6.448875e-06
MeL	autosomes	4	4_59470884	59470884	C	A	0.065	bivariate_only		8.897067e-06	8.897067e-06
MeL	autosomes	4	4_59483846	59483846	A	G	0.123	bivariate_only		9.992909e-06	9.992909e-06
MeL	autosomes	4	4_59544384	59544384	G	A	0.083	bivariate_only		8.836219e-06	8.836219e-06
MeL	autosomes	5	5_57964781	57964781	G	A	0.094	single_only	6.778137e-06		6.778137e-06
MeL	autosomes	6	6_2869816	2869816	A	C	0.351	both	4.986949e-06	3.486962e-06	3.486962e-06
MeL	autosomes	8	8_15513773	15513773	C	T	0.145	bivariate_only		5.275673e-06	5.275673e-06
MeL	autosomes	8	8_15579761	15579761	C	T	0.058	bivariate_only		9.40058e-06	9.40058e-06
MeL	autosomes	8	8_15638386	15638386	T	A	0.064	bivariate_only		6.722118e-06	6.722118e-06
MeL	autosomes	8	8_15690023	15690023	C	T	0.158	bivariate_only		6.719674e-06	6.719674e-06
MeL	autosomes	8	8_15718134	15718134	T	C	0.161	bivariate_only		4.906179e-06	4.906179e-06
MeL	autosomes	8	8_15795166	15795166	G	A	0.067	bivariate_only		4.259863e-06	4.259863e-06

TRAIT	SCOPE	CHR	SNP	BP	ALLELE1	ALLELE0	AF	GWAS SOURCE	P SINGLE	P JOINT	BEST P
MeL	autosomes	9	9_20591655	20591655	C	T	0.492	bivariate_only		4.665208e-06	4.665208e-06
MeL	autosomes	9	9_21230441	21230441	T	C	0.436	single_only	6.083867e-06		6.083867e-06
MeL	autosomes	9	9_21248107	21248107	G	A	0.370	single_only	7.282613e-06		7.282613e-06
MeL	autosomes	10	10_3508373	3508373	A	G	0.312	bivariate_only		9.257e-06	9.257e-06
MeL	autosomes	10	10_15917001	15917001	A	G	0.424	both	3.016597e-06	9.048228e-06	3.016597e-06
MeL	autosomes	17	17_10307375	10307375	C	G	0.121	single_only	4.206575e-06		4.206575e-06
MeL	autosomes	17	17_10318885	10318885	A	G	0.127	both	2.744374e-06	8.508006e-06	2.744374e-06
MeL	autosomes	17	17_10368305	10368305	C	G	0.124	single_only	2.121949e-06		2.121949e-06
MeL	autosomes	17	17_10404459	10404459	A	C	0.123	single_only	6.644963e-06		6.644963e-06
MeL	autosomes	17	17_10404889	10404889	T	C	0.145	single_only	6.374408e-06		6.374408e-06
MeL	autosomes	17	17_10405077	10405077	A	G	0.145	single_only	6.612469e-06		6.612469e-06
MeL	autosomes	17	17_10503767	10503767	T	G	0.150	single_only	7.567155e-06		7.567155e-06
MeL	autosomes	17	17_10509545	10509545	G	A	0.135	both	3.977986e-06	9.960788e-06	3.977986e-06
MeL	autosomes	18	18_2601049	2601049	G	A	0.370	single_only	8.437256e-06		8.437256e-06
MeL	autosomes	18	18_2602079	2602079	C	T	0.376	single_only	3.167597e-06		3.167597e-06
MeL	autosomes	18	18_2602114	2602114	G	A	0.379	single_only	8.030864e-06		8.030864e-06
MeL	autosomes	25	25_566688	566688	C	G	0.456	single_only	7.809611e-06		7.809611e-06

Table S1 (continued). Part C2. MeL prioritized candidate SNPs: effect and selection-region information.

TRAIT	SNP	BETA SINGLE	BETA JOINT TRAIT	SELECTION REGION IDS	SELECTION LABELS	SELECTION REGION STARTS	SELECTION REGION ENDS
MeL	1_102807177	1.660378e+00	1.697799e+00	REGION_0031	low_selected	102720001	102930000
MeL	1_103142183		1.392229e+00	REGION_0032	low_selected	103070001	103490000
MeL	1_103981328		1.057825e+00	REGION_0033	low_selected	103890001	104020000
MeL	2_16298204		-1.985256e+00	REGION_0058	high_selected	16210001	16320000
MeL	2_16313576		-1.668924e+00	REGION_0058	high_selected	16210001	16320000
MeL	2_56602250		-1.856952e+00	REGION_0062	high_selected	56260001	56620000
MeL	2_56602264		-1.856952e+00	REGION_0062	high_selected	56260001	56620000
MeL	2_62109190	-2.260251e+00		REGION_0065	high_selected	61620001	62330000
MeL	3_69045958		-6.054451e-01	REGION_0097	low_selected	68550001	69420000

TRAIT	SNP	BETA SINGLE	BETA JOINT TRAIT	SELECTION REGION IDS	SELECTION LABELS	SELECTION REGION STARTS	SELECTION REGION ENDS
MeL	4_4358701		-1.515644e+00	REGION_0107	high_selected	4340001	4480000
MeL	4_8841206		-2.073925e+00	REGION_0108	high_selected	8830001	9010000
MeL	4_8841620		-2.131694e+00	REGION_0108	high_selected	8830001	9010000
MeL	4_8841631		-1.903750e+00	REGION_0108	high_selected	8830001	9010000
MeL	4_8905235		-1.886896e+00	REGION_0108	high_selected	8830001	9010000
MeL	4_8920040		-1.989273e+00	REGION_0108	high_selected	8830001	9010000
MeL	4_8932225		-1.964880e+00	REGION_0108	high_selected	8830001	9010000
MeL	4_9004661		-2.183267e+00	REGION_0108	high_selected	8830001	9010000
MeL	4_30502663	1.236689e+00		REGION_0115	low_selected	30410001	30550000
MeL	4_46640140	-1.273383e+00		REGION_0116	high_selected	46580001	46760000
MeL	4_50856699		8.626344e-01	REGION_0121	high_selected	50780001	50970000
MeL	4_50922179	-1.295711e+00	-1.295418e+00	REGION_0121	high_selected	50780001	50970000
MeL	4_50924668		9.322087e-01	REGION_0121	high_selected	50780001	50970000
MeL	4_50926661		7.655264e-01	REGION_0121	high_selected	50780001	50970000
MeL	4_50931021		9.040042e-01	REGION_0121	high_selected	50780001	50970000
MeL	4_50960354		8.954602e-01	REGION_0121	high_selected	50780001	50970000
MeL	4_58233741	-1.438315e+00		REGION_0123	high_selected	58140001	58330000
MeL	4_58592454		-2.061560e+00	REGION_0124	high_selected	58580001	58750000
MeL	4_59470884		-2.274224e+00	REGION_0125	high_selected	59330001	59590000
MeL	4_59483846		-1.883132e+00	REGION_0125	high_selected	59330001	59590000
MeL	4_59544384		-2.216356e+00	REGION_0125	high_selected	59330001	59590000
MeL	5_57964781	-2.495838e+00		REGION_0135	high_selected	57920001	58060000
MeL	6_2869816	-1.449717e+00	-1.469013e+00	REGION_0136	high_selected	2700001	3070000
MeL	8_15513773		-1.647276e+00	REGION_0145	high_selected	15480001	15870000
MeL	8_15579761		-2.872170e+00	REGION_0145	high_selected	15480001	15870000
MeL	8_15638386		-2.976465e+00	REGION_0145	high_selected	15480001	15870000
MeL	8_15690023		-1.464092e+00	REGION_0145	high_selected	15480001	15870000
MeL	8_15718134		-1.654375e+00	REGION_0145	high_selected	15480001	15870000

TRAIT	SNP	BETA SINGLE	BETA JOINT TRAIT	SELECTION REGION IDS	SELECTION LABELS	SELECTION REGION STARTS	SELECTION REGION ENDS
MeL	8_15795166		-2.622567e+00	REGION_0145	high_selected	15480001	15870000
MeL	9_20591655		-3.561464e-01	REGION_0151	high_selected	20500001	20650000
MeL	9_21230441	1.141721e+00		REGION_0152	high_selected	21130001	21270000
MeL	9_21248107	1.272385e+00		REGION_0152	high_selected	21130001	21270000
MeL	10_3508373		1.258480e+00	REGION_0153	high_selected	3330001	3630000
MeL	10_15917001	-1.418182e+00	-1.426314e+00	REGION_0162	high_selected	15840001	15990000
MeL	17_10307375	2.149241e+00		REGION_0191	low_selected	10280001	10510000
MeL	17_10318885	2.131531e+00	2.127824e+00	REGION_0191	low_selected	10280001	10510000
MeL	17_10368305	2.306085e+00		REGION_0191	low_selected	10280001	10510000
MeL	17_10404459	2.266857e+00		REGION_0191	low_selected	10280001	10510000
MeL	17_10404889	2.122415e+00		REGION_0191	low_selected	10280001	10510000
MeL	17_10405077	2.126592e+00		REGION_0191	low_selected	10280001	10510000
MeL	17_10503767	2.105052e+00		REGION_0191	low_selected	10280001	10510000
MeL	17_10509545	2.128252e+00	2.119543e+00	REGION_0191	low_selected	10280001	10510000
MeL	18_2601049	1.243763e+00		REGION_0193	low_selected	2540001	2700000
MeL	18_2602079	1.373322e+00		REGION_0193	low_selected	2540001	2700000
MeL	18_2602114	1.308185e+00		REGION_0193	low_selected	2540001	2700000
MeL	25_566688	1.497040e+00		REGION_0197	low_selected	440001	570000

Table S1 (continued). Part D1. MeC prioritized candidate SNPs: association statistics.

TRAIT	SCOPE	CHR	SNP	BP	ALLELE1	ALLELE0	AF	GWAS SOURCE	P SINGLE	P JOINT	BEST P
MeC	autosomes	2	2_93938106	93938106	G	A	0.277	both	1.487907e-06	5.839495e-06	1.487907e-06
MeC	autosomes	3	3_45157581	45157581	T	C	0.388	both	5.291313e-06	6.790482e-07	6.790482e-07
MeC	autosomes	3	3_45206132	45206132	G	A	0.402	single_only	6.266275e-06		6.266275e-06
MeC	autosomes	4	4_51236931	51236931	A	G	0.376	single_only	8.1616e-06		8.1616e-06
MeC	autosomes	4	4_51317483	51317483	G	A	0.298	both	4.496044e-07	1.19462e-06	4.496044e-07
MeC	autosomes	4	4_52378327	52378327	T	C	0.473	both	1.801581e-06	5.131126e-06	1.801581e-06
MeC	autosomes	4	4_52426323	52426323	T	C	0.215	bivariate_only		7.73914e-06	7.73914e-06
MeC	autosomes	4	4_52438311	52438311	T	C	0.458	both	7.17521e-06	8.691005e-06	7.17521e-06
MeC	autosomes	4	4_52438385	52438385	G	T	0.461	bivariate_only		6.048303e-06	6.048303e-06

TRAIT	SCOPE	CHR	SNP	BP	ALLELE1	ALLELE0	AF	GWAS SOURCE	P SINGLE	P JOINT	BEST P
MeC	autosomes	4	4_52438421	52438421	G	C	0.468	bivariate_only		7.490187e-06	7.490187e-06
MeC	autosomes	4	4_52438433	52438433	T	A	0.463	bivariate_only		9.725098e-06	9.725098e-06
MeC	autosomes	4	4_52439598	52439598	C	T	0.474	both	2.59009e-06	4.419281e-06	2.59009e-06
MeC	autosomes	4	4_52451790	52451790	A	G	0.303	single_only	3.177579e-06		3.177579e-06
MeC	autosomes	4	4_52464126	52464126	C	T	0.429	both	3.181109e-06	3.145161e-06	3.145161e-06
MeC	autosomes	4	4_52467216	52467216	G	A	0.398	both	4.627274e-06	2.608426e-06	2.608426e-06
MeC	autosomes	4	4_52474334	52474334	T	A	0.438	both	2.118102e-07	4.093483e-07	2.118102e-07
MeC	autosomes	4	4_52474918	52474918	T	G	0.453	single_only	6.644375e-06		6.644375e-06
MeC	autosomes	4	4_52475158	52475158	G	A	0.426	bivariate_only		3.580036e-06	3.580036e-06
MeC	autosomes	4	4_52476344	52476344	A	G	0.479	both	1.043377e-06	1.396771e-06	1.043377e-06
MeC	autosomes	4	4_52479037	52479037	A	G	0.476	bivariate_only		5.220503e-06	5.220503e-06
MeC	autosomes	4	4_52481561	52481561	C	T	0.483	bivariate_only		9.196471e-06	9.196471e-06
MeC	autosomes	4	4_52482069	52482069	A	C	0.492	single_only	6.127771e-06		6.127771e-06
MeC	autosomes	4	4_52482110	52482110	G	A	0.447	both	2.593184e-06	3.145148e-06	2.593184e-06
MeC	autosomes	4	4_52488692	52488692	T	A	0.355	single_only	6.928884e-06		6.928884e-06
MeC	autosomes	4	4_52489714	52489714	G	A	0.426	both	9.937467e-06	9.542654e-06	9.542654e-06
MeC	autosomes	4	4_52490844	52490844	A	G	0.302	bivariate_only		8.540756e-06	8.540756e-06
MeC	autosomes	4	4_52491382	52491382	C	T	0.376	bivariate_only		6.77813e-06	6.77813e-06
MeC	autosomes	4	4_52491401	52491401	A	T	0.380	bivariate_only		5.0504e-06	5.0504e-06
MeC	autosomes	4	4_52491727	52491727	A	G	0.365	single_only	4.781788e-06		4.781788e-06
MeC	autosomes	4	4_52492921	52492921	A	G	0.388	single_only	3.218918e-06		3.218918e-06
MeC	autosomes	4	4_52498509	52498509	A	G	0.379	single_only	9.297157e-06		9.297157e-06
MeC	autosomes	4	4_52498560	52498560	T	C	0.380	both	1.557793e-07	6.376611e-07	1.557793e-07
MeC	autosomes	4	4_52498593	52498593	C	T	0.386	both	2.465436e-07	3.318954e-07	2.465436e-07
MeC	autosomes	4	4_52499329	52499329	T	C	0.417	both	2.1668e-07	1.218608e-07	1.218608e-07
MeC	autosomes	4	4_52663746	52663746	G	C	0.294	single_only	3.014837e-06		3.014837e-06
MeC	autosomes	4	4_56500442	56500442	G	A	0.083	single_only	4.410475e-06		4.410475e-06
MeC	autosomes	4	4_56605141	56605141	C	G	0.177	single_only	7.593171e-06		7.593171e-06
MeC	autosomes	4	4_58890405	58890405	C	T	0.070	bivariate_only		6.426291e-06	6.426291e-06
MeC	autosomes	4	4_59094671	59094671	G	A	0.103	bivariate_only		8.771049e-06	8.771049e-06

TRAIT	SCOPE	CHR	SNP	BP	ALLELE1	ALLELE0	AF	GWAS SOURCE	P SINGLE	P JOINT	BEST P
MeC	autosomes	4	4_59165705	59165705	T	A	0.100	bivariate_only		6.743925e-06	6.743925e-06
MeC	autosomes	4	4_59176477	59176477	T	C	0.094	bivariate_only		1.332099e-06	1.332099e-06
MeC	autosomes	4	4_59178040	59178040	A	G	0.109	bivariate_only		9.263001e-06	9.263001e-06
MeC	autosomes	4	4_59470884	59470884	C	A	0.065	bivariate_only		8.897067e-06	8.897067e-06
MeC	autosomes	4	4_59483846	59483846	A	G	0.123	bivariate_only		9.992909e-06	9.992909e-06
MeC	autosomes	4	4_59544384	59544384	G	A	0.083	bivariate_only		8.836219e-06	8.836219e-06
MeC	autosomes	5	5_20885150	20885150	A	G	0.421	both	1.804264e-06	3.492272e-06	1.804264e-06
MeC	autosomes	5	5_26207271	26207271	T	C	0.061	bivariate_only		5.242739e-06	5.242739e-06
MeC	autosomes	5	5_49728859	49728859	T	G	0.435	single_only	4.156583e-06		4.156583e-06
MeC	autosomes	6	6_3146546	3146546	A	G	0.342	bivariate_only		2.127048e-07	2.127048e-07
MeC	autosomes	6	6_3146578	3146578	T	C	0.067	bivariate_only		5.353719e-07	5.353719e-07
MeC	autosomes	6	6_3172247	3172247	C	G	0.327	bivariate_only		3.040555e-06	3.040555e-06
MeC	autosomes	6	6_3172268	3172268	C	T	0.327	bivariate_only		3.476239e-06	3.476239e-06
MeC	autosomes	9	9_19567769	19567769	T	C	0.435	single_only	9.598931e-06		9.598931e-06
MeC	autosomes	9	9_20921150	20921150	G	A	0.318	single_only	6.054842e-06		6.054842e-06
MeC	autosomes	9	9_20931265	20931265	T	C	0.370	both	8.524788e-06	6.257052e-06	6.257052e-06
MeC	autosomes	9	9_20976534	20976534	T	C	0.347	single_only	8.58782e-06		8.58782e-06
MeC	autosomes	11	11_7373893	7373893	C	G	0.418	both	1.290193e-07	4.908618e-08	4.908618e-08
MeC	autosomes	11	11_7373963	7373963	A	G	0.411	bivariate_only		3.234767e-06	3.234767e-06
MeC	autosomes	11	11_7374024	7374024	T	G	0.459	both	5.503927e-06	2.212882e-06	2.212882e-06
MeC	autosomes	11	11_7614407	7614407	C	T	0.416	bivariate_only		2.976927e-06	2.976927e-06
MeC	autosomes	28	28_521922	521922	A	G	0.489	single_only	2.723685e-06		2.723685e-06
MeC	autosomes	28	28_521968	521968	G	A	0.482	single_only	4.973451e-06		4.973451e-06

Table S1 (continued). Part D2. MeC prioritized candidate SNPs: effect and selection-region information.

TRAIT	SNP	BETA SINGLE	BETA JOINT TRAIT	SELECTION REGION IDS	SELECTION LABELS	SELECTION REGION STARTS	SELECTION REGION ENDS
MeC	2_93938106	-1.560612e-01	-1.561551e-01	REGION_0053;REGION_0054	high_selected;low_selected	93840001;93930001	93980000;94080000
MeC	3_45157581	-1.349037e-01	-1.357262e-01	REGION_0081	high_selected	45070001	45310000
MeC	3_45206132	-1.421780e-01		REGION_0081	high_selected	45070001	45310000
MeC	4_51236931	1.305650e-01		REGION_0099	low_selected	51110001	51470000

TRAIT	SNP	BETA SINGLE	BETA JOINT TRAIT	SELECTION REGION IDS	SELECTION LABELS	SELECTION REGION STARTS	SELECTION REGION ENDS
MeC	4_51317483	1.391499e-01	1.408228e-01	REGION_0099	low_selected	51110001	51470000
MeC	4_52378327	-1.224263e-01	-1.234317e-01	REGION_0100	high_selected	52310001	52500000
MeC	4_52426323		1.368196e-01	REGION_0100	high_selected	52310001	52500000
MeC	4_52438311	-1.322178e-01	-1.323442e-01	REGION_0100	high_selected	52310001	52500000
MeC	4_52438385		-1.232463e-01	REGION_0100	high_selected	52310001	52500000
MeC	4_52438421		-1.177455e-01	REGION_0100	high_selected	52310001	52500000
MeC	4_52438433		-1.171073e-01	REGION_0100	high_selected	52310001	52500000
MeC	4_52439598	1.340376e-01	1.347629e-01	REGION_0100	high_selected	52310001	52500000
MeC	4_52451790	-1.384545e-01		REGION_0100	high_selected	52310001	52500000
MeC	4_52464126	-1.302128e-01	-1.301702e-01	REGION_0100	high_selected	52310001	52500000
MeC	4_52467216	-1.221682e-01	-1.227677e-01	REGION_0100	high_selected	52310001	52500000
MeC	4_52474334	1.398322e-01	1.404478e-01	REGION_0100	high_selected	52310001	52500000
MeC	4_52474918	1.226130e-01		REGION_0100	high_selected	52310001	52500000
MeC	4_52475158		-1.245009e-01	REGION_0100	high_selected	52310001	52500000
MeC	4_52476344	1.237504e-01	1.227011e-01	REGION_0100	high_selected	52310001	52500000
MeC	4_52479037		-1.050622e-01	REGION_0100	high_selected	52310001	52500000
MeC	4_52481561		-1.103168e-01	REGION_0100	high_selected	52310001	52500000
MeC	4_52482069	-1.150872e-01		REGION_0100	high_selected	52310001	52500000
MeC	4_52482110	-1.175681e-01	-1.177271e-01	REGION_0100	high_selected	52310001	52500000
MeC	4_52488692	1.278987e-01		REGION_0100	high_selected	52310001	52500000
MeC	4_52489714	-1.247124e-01	-1.251870e-01	REGION_0100	high_selected	52310001	52500000
MeC	4_52490844		-1.199616e-01	REGION_0100	high_selected	52310001	52500000
MeC	4_52491382		-9.953298e-02	REGION_0100	high_selected	52310001	52500000
MeC	4_52491401		-9.048216e-02	REGION_0100	high_selected	52310001	52500000
MeC	4_52491727	1.275678e-01		REGION_0100	high_selected	52310001	52500000
MeC	4_52492921	-1.271487e-01		REGION_0100	high_selected	52310001	52500000
MeC	4_52498509	1.182884e-01		REGION_0100	high_selected	52310001	52500000
MeC	4_52498560	1.394184e-01	1.394224e-01	REGION_0100	high_selected	52310001	52500000
MeC	4_52498593	1.361737e-01	1.370377e-01	REGION_0100	high_selected	52310001	52500000
MeC	4_52499329	-1.379390e-01	-1.382903e-01	REGION_0100	high_selected	52310001	52500000
MeC	4_52663746	1.360151e-01		REGION_0101	low_selected	52560001	52680000

TRAIT	SNP	BETA SINGLE	BETA JOINT TRAIT	SELECTION REGION IDS	SELECTION LABELS	SELECTION REGION STARTS	SELECTION REGION ENDS
MeC	4_56500442	2.356712e-01		REGION_0102	low_selected	56380001	57810000
MeC	4_56605141	1.506296e-01		REGION_0102	low_selected	56380001	57810000
MeC	4_58890405		1.189506e-01	REGION_0107	low_selected	58720001	60450000
MeC	4_59094671		1.284670e-01	REGION_0107	low_selected	58720001	60450000
MeC	4_59165705		9.617676e-02	REGION_0107	low_selected	58720001	60450000
MeC	4_59176477		1.652374e-01	REGION_0107	low_selected	58720001	60450000
MeC	4_59178040		1.309624e-01	REGION_0107	low_selected	58720001	60450000
MeC	4_59470884		1.832941e-01	REGION_0107	low_selected	58720001	60450000
MeC	4_59483846		1.699541e-01	REGION_0107	low_selected	58720001	60450000
MeC	4_59544384		1.590409e-01	REGION_0107	low_selected	58720001	60450000
MeC	5_20885150	-1.286515e-01	-1.300841e-01	REGION_0115	low_selected	20780001	20930000
MeC	5_26207271		1.937341e-01	REGION_0119	high_selected	26120001	26390000
MeC	5_49728859	1.409835e-01		REGION_0132	high_selected	49650001	49760000
MeC	6_3146546		-8.679306e-02	REGION_0133	high_selected	2870001	3190000
MeC	6_3146578		1.227128e-01	REGION_0133	high_selected	2870001	3190000
MeC	6_3172247		-4.552881e-02	REGION_0133	high_selected	2870001	3190000
MeC	6_3172268		-6.736753e-02	REGION_0133	high_selected	2870001	3190000
MeC	9_19567769	-1.281782e-01		REGION_0162	high_selected	19360001	19620000
MeC	9_20921150	-1.223831e-01		REGION_0164	high_selected	20770001	21020000
MeC	9_20931265	-1.234352e-01	-1.230547e-01	REGION_0164	high_selected	20770001	21020000
MeC	9_20976534	1.309974e-01		REGION_0164	high_selected	20770001	21020000
MeC	11_7373893	1.470800e-01	1.479654e-01	REGION_0170	high_selected	7300001	7430000
MeC	11_7373963		1.243855e-01	REGION_0170	high_selected	7300001	7430000
MeC	11_7374024	1.297600e-01	1.303609e-01	REGION_0170	high_selected	7300001	7430000
MeC	11_7614407		6.770742e-02	REGION_0171	low_selected	7470001	7630000
MeC	28_521922	1.588721e-01		REGION_0184	low_selected	300001	580000
MeC	28_521968	1.497143e-01		REGION_0184	low_selected	300001	580000

Table S1 (continued). Part E1. LD-refined autosomal signals: lead-variant information.

SIGNAL ID	TRAIT	SCOPE	CHR	LEAD SNP	LEAD BP	LEAD BEST P	LEAD GWAS SOURCE
MEC_AUTOSOMES_SIG008	MeC	autosomes	2	2_93938106	93938106	1.48791e-06	both
MEC_AUTOSOMES_SIG006	MeC	autosomes	3	3_45157581	45157581	6.79048e-07	both
MEC_AUTOSOMES_SIG016	MeC	autosomes	3	3_45206132	45206132	6.26628e-06	single_only
MEC_AUTOSOMES_SIG004	MeC	autosomes	4	4_51317483	51317483	4.49604e-07	both
MEC_AUTOSOMES_SIG017	MeC	autosomes	4	4_52426323	52426323	7.73914e-06	bivariate_only
MEC_AUTOSOMES_SIG013	MeC	autosomes	4	4_52491401	52491401	5.0504e-06	bivariate_only
MEC_AUTOSOMES_SIG002	MeC	autosomes	4	4_52499329	52499329	1.21861e-07	both
MEC_AUTOSOMES_SIG012	MeC	autosomes	4	4_56500442	56500442	4.41048e-06	single_only
MEC_AUTOSOMES_SIG007	MeC	autosomes	4	4_59176477	59176477	1.3321e-06	bivariate_only
MEC_AUTOSOMES_SIG009	MeC	autosomes	5	5_20885150	20885150	1.80426e-06	both
MEC_AUTOSOMES_SIG014	MeC	autosomes	5	5_26207271	26207271	5.24274e-06	bivariate_only
MEC_AUTOSOMES_SIG011	MeC	autosomes	5	5_49728859	49728859	4.15658e-06	single_only
MEC_AUTOSOMES_SIG003	MeC	autosomes	6	6_3146546	3146546	2.12705e-07	bivariate_only
MEC_AUTOSOMES_SIG005	MeC	autosomes	6	6_3146578	3146578	5.35372e-07	bivariate_only
MEC_AUTOSOMES_SIG019	MeC	autosomes	9	9_19567769	19567769	9.59893e-06	single_only
MEC_AUTOSOMES_SIG015	MeC	autosomes	9	9_20921150	20921150	6.05484e-06	single_only
MEC_AUTOSOMES_SIG018	MeC	autosomes	9	9_20976534	20976534	8.58782e-06	single_only
MEC_AUTOSOMES_SIG001	MeC	autosomes	11	11_7373893	7373893	4.90862e-08	both
MEC_AUTOSOMES_SIG010	MeC	autosomes	28	28_521922	521922	2.72369e-06	single_only
MEL_AUTOSOMES_SIG002	MeL	autosomes	1	1_102807177	102807177	4.90762e-07	both
MEL_AUTOSOMES_SIG005	MeL	autosomes	1	1_103981328	103981328	2.43054e-06	bivariate_only
MEL_AUTOSOMES_SIG013	MeL	autosomes	2	2_16298204	16298204	5.83145e-06	bivariate_only
MEL_AUTOSOMES_SIG025	MeL	autosomes	2	2_56602250	56602250	9.61078e-06	bivariate_only
MEL_AUTOSOMES_SIG022	MeL	autosomes	2	2_62109190	62109190	9.11991e-06	single_only
MEL_AUTOSOMES_SIG020	MeL	autosomes	3	3_69045958	69045958	7.95176e-06	bivariate_only
MEL_AUTOSOMES_SIG024	MeL	autosomes	4	4_4358701	4358701	9.52974e-06	bivariate_only
MEL_AUTOSOMES_SIG001	MeL	autosomes	4	4_8841620	8841620	6.48835e-08	bivariate_only
MEL_AUTOSOMES_SIG012	MeL	autosomes	4	4_30502663	30502663	5.46432e-06	single_only
MEL_AUTOSOMES_SIG018	MeL	autosomes	4	4_46640140	46640140	7.09416e-06	single_only
MEL_AUTOSOMES_SIG017	MeL	autosomes	4	4_50922179	50922179	7.01612e-06	both
MEL_AUTOSOMES_SIG003	MeL	autosomes	4	4_50926661	50926661	6.15706e-07	bivariate_only
MEL_AUTOSOMES_SIG026	MeL	autosomes	4	4_58233741	58233741	9.62831e-06	single_only
MEL_AUTOSOMES_SIG015	MeL	autosomes	4	4_58592454	58592454	6.44888e-06	bivariate_only
MEL_AUTOSOMES_SIG021	MeL	autosomes	4	4_59544384	59544384	8.83622e-06	bivariate_only
MEL_AUTOSOMES_SIG016	MeL	autosomes	5	5_57964781	57964781	6.77814e-06	single_only
MEL_AUTOSOMES_SIG008	MeL	autosomes	6	6_2869816	2869816	3.48696e-06	both

SIGNAL ID	TRAIT	SCOPE	CHR	LEAD SNP	LEAD BP	LEAD BEST P	LEAD GWAS SOURCE
MEL_AUTOSOMES_SIG011	MeL	autosomes	8	8_15513773	15513773	5.27567e-06	bivariate_only
MEL_AUTOSOMES_SIG009	MeL	autosomes	8	8_15795166	15795166	4.25986e-06	bivariate_only
MEL_AUTOSOMES_SIG010	MeL	autosomes	9	9_20591655	20591655	4.66521e-06	bivariate_only
MEL_AUTOSOMES_SIG014	MeL	autosomes	9	9_21230441	21230441	6.08387e-06	single_only
MEL_AUTOSOMES_SIG023	MeL	autosomes	10	10_3508373	3508373	9.257e-06	bivariate_only
MEL_AUTOSOMES_SIG006	MeL	autosomes	10	10_15917001	15917001	3.0166e-06	both
MEL_AUTOSOMES_SIG004	MeL	autosomes	17	17_10368305	10368305	2.12195e-06	single_only
MEL_AUTOSOMES_SIG007	MeL	autosomes	18	18_2602079	2602079	3.1676e-06	single_only
MEL_AUTOSOMES_SIG019	MeL	autosomes	25	25_566688	566688	7.80961e-06	single_only

Table S1 (continued). Part E2. LD-refined autosomal signals: membership and interval information.

SIGNAL ID	LEAD SNP	N PRIORITY MEMBERS R2 GE 0.2	PRIORITY MEMBERS	N HIGH LD VARIANTS R2 GE 0.8	LD REFINED START	LD REFINED END
MEC_AUTOSOMES_SIG008	2_93938106	1	2_93938106	1	93938106	93938106
MEC_AUTOSOMES_SIG006	3_45157581	1	3_45157581	2	45157581	45157632
MEC_AUTOSOMES_SIG016	3_45206132	1	3_45206132	1	45206132	45206132
MEC_AUTOSOMES_SIG004	4_51317483	2	4_51236931;4_51317483	1	51317483	51317483
MEC_AUTOSOMES_SIG017	4_52426323	1	4_52426323	1	52426323	52426323
MEC_AUTOSOMES_SIG013	4_52491401	2	4_52491382;4_52491401	2	52491382	52491401
MEC_AUTOSOMES_SIG002	4_52499329	27	4_52378327;4_52438311;4_52438385;4_52438421;4_52438433;4_52439598;4_52451790;4_52464126;4_52467216;4_52474334;4_52474918;4_52475158;4_52476344;4_52479037;4_52481561;4_52482069;4_52482110;4_52488692;4_52489714;4_52490844;4_52491727;4_52492921;4_52498509;4_52498560;4_52498593;4_52499329;4_52663746	1	52499329	52499329
MEC_AUTOSOMES_SIG012	4_56500442	2	4_56500442;4_56605141	1	56500442	56500442
MEC_AUTOSOMES_SIG007	4_59176477	8	4_58890405;4_59094671;4_59165705;4_59176477;4_59178040;4_59470884;4_59483846;4_59544384	1	59176477	59176477
MEC_AUTOSOMES_SIG009	5_20885150	1	5_20885150	1	20885150	20885150
MEC_AUTOSOMES_SIG014	5_26207271	1	5_26207271	1	26207271	26207271
MEC_AUTOSOMES_SIG011	5_49728859	1	5_49728859	1	49728859	49728859
MEC_AUTOSOMES_SIG003	6_3146546	3	6_3146546;6_3172247;6_3172268	1	3146546	3146546
MEC_AUTOSOMES_SIG005	6_3146578	1	6_3146578	1	3146578	3146578
MEC_AUTOSOMES_SIG019	9_19567769	1	9_19567769	1	19567769	19567769
MEC_AUTOSOMES_SIG015	9_20921150	2	9_20921150;9_20931265	1	20921150	20921150
MEC_AUTOSOMES_SIG018	9_20976534	1	9_20976534	1	20976534	20976534
MEC_AUTOSOMES_SIG001	11_7373893	4	11_7373893;11_7373963;11_7374024;11_7614407	1	7373893	7373893
MEC_AUTOSOMES_SIG010	28_521922	2	28_521922;28_521968	2	521922	521968
MEL_AUTOSOMES_SIG002	1_102807177	2	1_102807177;1_103142183	1	102807177	102807177
MEL_AUTOSOMES_SIG005	1_103981328	1	1_103981328	2	103981309	103981328

SIGNAL ID	LEAD SNP	N PRIORITY MEMBERS R2 GE 0.2	PRIORITY MEMBERS	N HIGH LD VARIANTS R2 GE 0.8	LD REFINED START	LD REFINED END
MEL_AUTOSOMES_SIG013	2_16298204	2	2_16298204;2_16313576	1	16298204	16298204
MEL_AUTOSOMES_SIG025	2_56602250	2	2_56602250;2_56602264	3	56602236	56602264
MEL_AUTOSOMES_SIG022	2_62109190	1	2_62109190	1	62109190	62109190
MEL_AUTOSOMES_SIG020	3_69045958	1	3_69045958	1	69045958	69045958
MEL_AUTOSOMES_SIG024	4_4358701	1	4_4358701	1	4358701	4358701
MEL_AUTOSOMES_SIG001	4_8841620	7	4_8841206;4_8841620;4_8841631;4_8905235;4_8920040;4_8932225;4_9004661	2	8841620	8841631
MEL_AUTOSOMES_SIG012	4_30502663	1	4_30502663	1	30502663	30502663
MEL_AUTOSOMES_SIG018	4_46640140	1	4_46640140	1	46640140	46640140
MEL_AUTOSOMES_SIG017	4_50922179	1	4_50922179	1	50922179	50922179
MEL_AUTOSOMES_SIG003	4_50926661	5	4_50856699;4_50924668;4_50926661;4_50931021;4_50960354	1	50926661	50926661
MEL_AUTOSOMES_SIG026	4_58233741	1	4_58233741	1	58233741	58233741
MEL_AUTOSOMES_SIG015	4_58592454	1	4_58592454	1	58592454	58592454
MEL_AUTOSOMES_SIG021	4_59544384	3	4_59470884;4_59483846;4_59544384	2	59544379	59544384
MEL_AUTOSOMES_SIG016	5_57964781	1	5_57964781	1	57964781	57964781
MEL_AUTOSOMES_SIG008	6_2869816	1	6_2869816	1	2869816	2869816
MEL_AUTOSOMES_SIG011	8_15513773	1	8_15513773	1	15513773	15513773
MEL_AUTOSOMES_SIG009	8_15795166	5	8_15579761;8_15638386;8_15690023;8_15718134;8_15795166	1	15795166	15795166
MEL_AUTOSOMES_SIG010	9_20591655	1	9_20591655	1	20591655	20591655
MEL_AUTOSOMES_SIG014	9_21230441	2	9_21230441;9_21248107	1	21230441	21230441
MEL_AUTOSOMES_SIG023	10_3508373	1	10_3508373	1	3508373	3508373
MEL_AUTOSOMES_SIG006	10_15917001	1	10_15917001	1	15917001	15917001
MEL_AUTOSOMES_SIG004	17_10368305	8	17_10307375;17_10318885;17_10368305;17_10404459;17_10404889;17_10405077;17_10503767;17_10509545	1	10368305	10368305
MEL_AUTOSOMES_SIG007	18_2602079	3	18_2601049;18_2602079;18_2602114	2	2602079	2602114
MEL_AUTOSOMES_SIG019	25_566688	1	25_566688	2	566663	566688

Table S1 (continued). Part F. MeL chromosome Z signal.

SIGNAL ID	TRAIT	SCOPE	CHR	LEAD SNP	LEAD BP	LEAD BEST P	LEAD GWAS SOURCE	N PRIORITY MEMBERS R2 GE 0.2	PRIORITY MEMBERS	N HIGH LD VARIANTS R2 GE 0.8	LD REFINED START	LD REFINED END
MEL_CHRZ_SIG001	MeL	chrZ	Z	chrZ_24478892	24478892	3.62441e-07	bivariate_only	1	chrZ_24478892	1	24478892	24478892

Table S1 (continued). Part G1. Integrated candidate variants: locus, priority, and LD information.

CHR	BP	SNP	REF	ALT	IS PRIORITY	PRIORITY SETS	LD SIGNALS	TRAITS	SCOPES	MAX R2 TO ANY LEAD
1	102807177	1_102807177	C	T	1	MeL:autosomes	MEL_AUTOSOMES_SIG002	MeL	autosomes	1
1	103142183	1_103142183	C	T	1	MeL:autosomes		MeL	autosomes	0

CHR	BP	SNP	REF	ALT	IS PRIORITY	PRIORITY SETS	LD SIGNALS	TRAITS	SCOPES	MAX R2 TO ANY LEAD
1	103981309	1_103981309	C	T	0		MEL_AUTOSOMES_SIG005	MeL	autosomes	0.909834
1	103981328	1_103981328	A	C	1	MeL:autosomes	MEL_AUTOSOMES_SIG005	MeL	autosomes	1
2	16298204	2_16298204	G	A	1	MeL:autosomes	MEL_AUTOSOMES_SIG013	MeL	autosomes	1
2	16313576	2_16313576	T	C	1	MeL:autosomes		MeL	autosomes	0
2	56602236	2_56602236	C	T	0		MEL_AUTOSOMES_SIG025	MeL	autosomes	0.897632
2	56602250	2_56602250	T	A	1	MeL:autosomes	MEL_AUTOSOMES_SIG025	MeL	autosomes	1
2	56602264	2_56602264	A	C	1	MeL:autosomes	MEL_AUTOSOMES_SIG025	MeL	autosomes	1
2	62109190	2_62109190	T	C	1	MeL:autosomes	MEL_AUTOSOMES_SIG022	MeL	autosomes	1
2	93938106	2_93938106	A	G	1	MeC:autosomes	MEC_AUTOSOMES_SIG008	MeC	autosomes	1
3	45157581	3_45157581	C	T	1	MeC:autosomes	MEC_AUTOSOMES_SIG006	MeC	autosomes	1
3	45157632	3_45157632	T	G	0		MEC_AUTOSOMES_SIG006	MeC	autosomes	0.830388
3	45206132	3_45206132	A	G	1	MeC:autosomes	MEC_AUTOSOMES_SIG016	MeC	autosomes	1
3	69045958	3_69045958	G	C	1	MeL:autosomes	MEL_AUTOSOMES_SIG020	MeL	autosomes	1
4	4358701	4_4358701	G	A	1	MeL:autosomes	MEL_AUTOSOMES_SIG024	MeL	autosomes	1
4	8841206	4_8841206	G	A	1	MeL:autosomes		MeL	autosomes	0
4	8841620	4_8841620	T	C	1	MeL:autosomes	MEL_AUTOSOMES_SIG001	MeL	autosomes	1
4	8841631	4_8841631	A	C	1	MeL:autosomes	MEL_AUTOSOMES_SIG001	MeL	autosomes	0.944376
4	8905235	4_8905235	T	C	1	MeL:autosomes		MeL	autosomes	0
4	8920040	4_8920040	A	G	1	MeL:autosomes		MeL	autosomes	0
4	8932225	4_8932225	C	G	1	MeL:autosomes		MeL	autosomes	0
4	9004661	4_9004661	T	C	1	MeL:autosomes		MeL	autosomes	0
4	30502663	4_30502663	A	G	1	MeL:autosomes	MEL_AUTOSOMES_SIG012	MeL	autosomes	1
4	46640140	4_46640140	C	T	1	MeL:autosomes	MEL_AUTOSOMES_SIG018	MeL	autosomes	1
4	50856699	4_50856699	T	C	1	MeL:autosomes		MeL	autosomes	0
4	50922179	4_50922179	C	G	1	MeL:autosomes	MEL_AUTOSOMES_SIG017	MeL	autosomes	1
4	50924668	4_50924668	T	C	1	MeL:autosomes		MeL	autosomes	0
4	50926661	4_50926661	G	A	1	MeL:autosomes	MEL_AUTOSOMES_SIG003	MeL	autosomes	1
4	50931021	4_50931021	T	A	1	MeL:autosomes		MeL	autosomes	0
4	50960354	4_50960354	G	A	1	MeL:autosomes		MeL	autosomes	0
4	51236931	4_51236931	G	A	1	MeC:autosomes		MeC	autosomes	0
4	51317483	4_51317483	A	G	1	MeC:autosomes	MEC_AUTOSOMES_SIG004	MeC	autosomes	1
4	52378327	4_52378327	C	T	1	MeC:autosomes		MeC	autosomes	0

CHR	BP	SNP	REF	ALT	IS PRIORITY	PRIORITY SETS	LD SIGNALS	TRAITS	SCOPES	MAX R2 TO ANY LEAD
4	52426323	4_52426323	C	T	1	MeC:autosomes	MEC_AUTOSOMES_SIG017	MeC	autosomes	1
4	52438311	4_52438311	C	T	1	MeC:autosomes		MeC	autosomes	0
4	52438385	4_52438385	T	G	1	MeC:autosomes		MeC	autosomes	0
4	52438421	4_52438421	C	G	1	MeC:autosomes		MeC	autosomes	0
4	52438433	4_52438433	A	T	1	MeC:autosomes		MeC	autosomes	0
4	52439598	4_52439598	C	T	1	MeC:autosomes		MeC	autosomes	0
4	52451790	4_52451790	G	A	1	MeC:autosomes		MeC	autosomes	0
4	52464126	4_52464126	T	C	1	MeC:autosomes		MeC	autosomes	0
4	52467216	4_52467216	A	G	1	MeC:autosomes		MeC	autosomes	0
4	52474334	4_52474334	T	A	1	MeC:autosomes		MeC	autosomes	0
4	52474918	4_52474918	T	G	1	MeC:autosomes		MeC	autosomes	0
4	52475158	4_52475158	A	G	1	MeC:autosomes		MeC	autosomes	0
4	52476344	4_52476344	A	G	1	MeC:autosomes		MeC	autosomes	0
4	52479037	4_52479037	G	A	1	MeC:autosomes		MeC	autosomes	0
4	52481561	4_52481561	T	C	1	MeC:autosomes		MeC	autosomes	0
4	52482069	4_52482069	C	A	1	MeC:autosomes		MeC	autosomes	0
4	52482110	4_52482110	A	G	1	MeC:autosomes		MeC	autosomes	0
4	52488692	4_52488692	T	A	1	MeC:autosomes		MeC	autosomes	0
4	52489714	4_52489714	A	G	1	MeC:autosomes		MeC	autosomes	0
4	52490844	4_52490844	G	A	1	MeC:autosomes		MeC	autosomes	0
4	52491382	4_52491382	T	C	1	MeC:autosomes	MEC_AUTOSOMES_SIG013	MeC	autosomes	0.87229
4	52491401	4_52491401	T	A	1	MeC:autosomes	MEC_AUTOSOMES_SIG013	MeC	autosomes	1
4	52491727	4_52491727	A	G	1	MeC:autosomes		MeC	autosomes	0
4	52492921	4_52492921	G	A	1	MeC:autosomes		MeC	autosomes	0
4	52498509	4_52498509	A	G	1	MeC:autosomes		MeC	autosomes	0
4	52498560	4_52498560	T	C	1	MeC:autosomes		MeC	autosomes	0
4	52498593	4_52498593	C	T	1	MeC:autosomes		MeC	autosomes	0
4	52499329	4_52499329	C	T	1	MeC:autosomes	MEC_AUTOSOMES_SIG002	MeC	autosomes	1

CHR	BP	SNP	REF	ALT	IS PRIORITY	PRIORITY SETS	LD SIGNALS	TRAITS	SCOPES	MAX R2 TO ANY LEAD
4	52663746	4_52663746	C	G	1	MeC:autosomes		MeC	autosomes	0
4	56500442	4_56500442	A	G	1	MeC:autosomes	MEC_AUTOSOMES_SIG012	MeC	autosomes	1
4	56605141	4_56605141	C	G	1	MeC:autosomes		MeC	autosomes	0
4	58233741	4_58233741	T	C	1	MeL:autosomes	MEL_AUTOSOMES_SIG026	MeL	autosomes	1
4	58592454	4_58592454	A	G	1	MeL:autosomes	MEL_AUTOSOMES_SIG015	MeL	autosomes	1
4	58890405	4_58890405	T	C	1	MeC:autosomes		MeC	autosomes	0
4	59094671	4_59094671	A	G	1	MeC:autosomes		MeC	autosomes	0
4	59165705	4_59165705	A	T	1	MeC:autosomes		MeC	autosomes	0
4	59176477	4_59176477	C	T	1	MeC:autosomes	MEC_AUTOSOMES_SIG007	MeC	autosomes	1
4	59178040	4_59178040	G	A	1	MeC:autosomes		MeC	autosomes	0
4	59470884	4_59470884	A	C	1	MeC:autosomes;MeL:autosomes		MeC;MeL	autosomes	0
4	59483846	4_59483846	G	A	1	MeC:autosomes;MeL:autosomes		MeC;MeL	autosomes	0
4	59544379	4_59544379	C	T	0		MEL_AUTOSOMES_SIG021	MeL	autosomes	0.948668
4	59544384	4_59544384	A	G	1	MeC:autosomes;MeL:autosomes	MEL_AUTOSOMES_SIG021	MeC;MeL	autosomes	1
5	20885150	5_20885150	G	A	1	MeC:autosomes	MEC_AUTOSOMES_SIG009	MeC	autosomes	1
5	26207271	5_26207271	T	C	1	MeC:autosomes	MEC_AUTOSOMES_SIG014	MeC	autosomes	1
5	49728859	5_49728859	T	G	1	MeC:autosomes	MEC_AUTOSOMES_SIG011	MeC	autosomes	1
5	57964781	5_57964781	A	G	1	MeL:autosomes	MEL_AUTOSOMES_SIG016	MeL	autosomes	1
6	2869816	6_2869816	A	C	1	MeL:autosomes	MEL_AUTOSOMES_SIG008	MeL	autosomes	1
6	3146546	6_3146546	A	G	1	MeC:autosomes	MEC_AUTOSOMES_SIG003	MeC	autosomes	1
6	3146578	6_3146578	T	C	1	MeC:autosomes	MEC_AUTOSOMES_SIG005	MeC	autosomes	1
6	3172247	6_3172247	C	G	1	MeC:autosomes		MeC	autosomes	0
6	3172268	6_3172268	C	T	1	MeC:autosomes		MeC	autosomes	0
8	15513773	8_15513773	T	C	1	MeL:autosomes	MEL_AUTOSOMES_SIG011	MeL	autosomes	1
8	15579761	8_15579761	T	C	1	MeL:autosomes		MeL	autosomes	0
8	15638386	8_15638386	A	T	1	MeL:autosomes		MeL	autosomes	0
8	15690023	8_15690023	T	C	1	MeL:autosomes		MeL	autosomes	0
8	15718134	8_15718134	C	T	1	MeL:autosomes		MeL	autosomes	0
8	15795166	8_15795166	A	G	1	MeL:autosomes	MEL_AUTOSOMES_SIG009	MeL	autosomes	1
9	19567769	9_19567769	T	C	1	MeC:autosomes	MEC_AUTOSOMES_SIG019	MeC	autosomes	1
9	20591655	9_20591655	T	C	1	MeL:autosomes	MEL_AUTOSOMES_SIG010	MeL	autosomes	1
9	20921150	9_20921150	A	G	1	MeC:autosomes	MEC_AUTOSOMES_SIG015	MeC	autosomes	1

CHR	BP	SNP	REF	ALT	IS PRIORITY	PRIORITY SETS	LD SIGNALS	TRAITS	SCOPES	MAX R2 TO ANY LEAD
9	20931265	9_20931265	C	T	1	MeC:autosomes		MeC	autosomes	0
9	20976534	9_20976534	C	T	1	MeC:autosomes	MEC_AUTOSOMES_SIG018	MeC	autosomes	1
9	21230441	9_21230441	T	C	1	MeL:autosomes	MEL_AUTOSOMES_SIG014	MeL	autosomes	1
9	21248107	9_21248107	G	A	1	MeL:autosomes		MeL	autosomes	0
10	3508373	10_3508373	G	A	1	MeL:autosomes	MEL_AUTOSOMES_SIG023	MeL	autosomes	1
10	15917001	10_15917001	A	G	1	MeL:autosomes	MEL_AUTOSOMES_SIG006	MeL	autosomes	1
11	7373893	11_7373893	G	C	1	MeC:autosomes	MEC_AUTOSOMES_SIG001	MeC	autosomes	1
11	7373963	11_7373963	G	A	1	MeC:autosomes		MeC	autosomes	0
11	7374024	11_7374024	G	T	1	MeC:autosomes		MeC	autosomes	0
11	7614407	11_7614407	T	C	1	MeC:autosomes		MeC	autosomes	0
17	10307375	17_10307375	G	C	1	MeL:autosomes		MeL	autosomes	0
17	10318885	17_10318885	G	A	1	MeL:autosomes		MeL	autosomes	0
17	10368305	17_10368305	G	C	1	MeL:autosomes	MEL_AUTOSOMES_SIG004	MeL	autosomes	1
17	10404459	17_10404459	C	A	1	MeL:autosomes		MeL	autosomes	0
17	10404889	17_10404889	C	T	1	MeL:autosomes		MeL	autosomes	0
17	10405077	17_10405077	G	A	1	MeL:autosomes		MeL	autosomes	0
17	10503767	17_10503767	G	T	1	MeL:autosomes		MeL	autosomes	0
17	10509545	17_10509545	A	G	1	MeL:autosomes		MeL	autosomes	0
18	2601049	18_2601049	G	A	1	MeL:autosomes		MeL	autosomes	0
18	2602079	18_2602079	C	T	1	MeL:autosomes	MEL_AUTOSOMES_SIG007	MeL	autosomes	1
18	2602114	18_2602114	G	A	1	MeL:autosomes	MEL_AUTOSOMES_SIG007	MeL	autosomes	0.83105
25	566663	25_566663	A	C	0		MEL_AUTOSOMES_SIG019	MeL	autosomes	0.803948
25	566688	25_566688	C	G	1	MeL:autosomes	MEL_AUTOSOMES_SIG019	MeL	autosomes	1
28	521922	28_521922	G	A	1	MeC:autosomes	MEC_AUTOSOMES_SIG010	MeC	autosomes	1
28	521968	28_521968	A	G	1	MeC:autosomes	MEC_AUTOSOMES_SIG010	MeC	autosomes	0.885105
Z	24478892	chrZ_24478892	G	C	1	MeL:chrZ	MEL_CHRZ_SIG001	MeL	chrZ	1

Table S1 (continued). Part G2. Integrated candidate variants: functional annotation and gene mapping.

CHR	BP	SNP	MAX IMPACT	TOP ANNOTATION	GENE IDS	GENE NAMES	ALL EFFECTS
1	102807177	1_102807177	MODIFIER	intron_variant	ENSGALG00010010915;GENE_ENSGALE00010106181;GENE_ENSGALE00010106289;GENE_ENSGALE00010106312;gene:ENSGALG00010010915	APP;ENSGALE00010106181;ENSGALE00010106289;ENSGALE00010106312;gene:ENSGALG00010010915	intragenic_variant;intron_variant;non_coding_transcript_variant
1	103142183	1_103142183	MODIFIER	intergenic_region	ENSGALG00010010659-GENE_ENSGALE00010138381	ADAMTS1-ENSGALE00010138381	intergenic_region

CHR	BP	SNP	MAX IMPACT	TOP ANNOTATION	GENE IDS	GENE NAMES	ALL EFFECTS
1	103981309	1_103981309	MODIFIER	intergenic_region	GENE_ENSGALE00010141744-GENE_ENSGALE00010138869	ENSGALE00010141744-ENSGALE00010138869	intergenic_region
1	103981328	1_103981328	MODIFIER	intergenic_region	GENE_ENSGALE00010141744-GENE_ENSGALE00010138869	ENSGALE00010141744-ENSGALE00010138869	intergenic_region
2	16298204	2_16298204	MODIFIER	intron_variant	ENSGALG00010005447;GENE_ENSGALE00010052553;GENE_ENSGALE00010052556;GENE_ENSGALE00010052608;GENE_ENSGALE00010052619;gene:ENSGALG00010005447	ENSGALE00010052553;ENSGALE00010052556;ENSGALE00010052608;ENSGALE00010052619;MYO3A;gene:ENSGALG00010005447	intragenic_variant;intron_variant;non_coding_transcript_variant
2	16313576	2_16313576	MODIFIER	downstream_gene_variant	ENSGALG00010005447-GENE_ENSGALE00010062134;GENE_ENSGALE00010062134	ENSGALE00010062134;MYO3A-ENSGALE00010062134	downstream_gene_variant;intergenic_region
2	56602236	2_56602236	MODIFIER	upstream_gene_variant	ENSGALG00010007319-GENE_ENSGALE00010069718;GENE_ENSGALE00010069578;GENE_ENSGALE00010069580;GENE_ENSGALE00010069590;GENE_ENSGALE00010069608;GENE_ENSGALE00010069647;GENE_ENSGALE00010069655;gene:ENSGALG00010007319	ATP9B-ENSGALE00010069718;ENSGALE00010069578;ENSGALE00010069580;ENSGALE00010069590;ENSGALE00010069608;ENSGALE00010069647;ENSGALE00010069655;gene:ENSGALG00010007319	downstream_gene_variant;intergenic_region;upstream_gene_variant
2	56602250	2_56602250	MODIFIER	upstream_gene_variant	ENSGALG00010007319-GENE_ENSGALE00010069718;GENE_ENSGALE00010069578;GENE_ENSGALE00010069580;GENE_ENSGALE00010069590;GENE_ENSGALE00010069608;GENE_ENSGALE00010069647;GENE_ENSGALE00010069655;gene:ENSGALG00010007319	ATP9B-ENSGALE00010069718;ENSGALE00010069578;ENSGALE00010069580;ENSGALE00010069590;ENSGALE00010069608;ENSGALE00010069647;ENSGALE00010069655;gene:ENSGALG00010007319	downstream_gene_variant;intergenic_region;upstream_gene_variant
2	56602264	2_56602264	MODIFIER	upstream_gene_variant	ENSGALG00010007319-GENE_ENSGALE00010069718;GENE_ENSGALE00010069578;GENE_ENSGALE00010069580;GENE_ENSGALE00010069590;GENE_ENSGALE00010069608;GENE_ENSGALE00010069647;GENE_ENSGALE00010069655;gene:ENSGALG00010007319	ATP9B-ENSGALE00010069718;ENSGALE00010069578;ENSGALE00010069580;ENSGALE00010069590;ENSGALE00010069608;ENSGALE00010069647;ENSGALE00010069655;gene:ENSGALG00010007319	downstream_gene_variant;intergenic_region;upstream_gene_variant
2	62109190	2_62109190	MODIFIER	intron_variant	ENSGALG00010010146;GENE_ENSGALE00010097921;GENE_ENSGALE00010097955;GENE_ENSGALE00010098094;GENE_ENSGALE00010098228;gene:ENSGALG00010010146	ENSGALE00010097921;ENSGALE00010097955;ENSGALE00010098094;ENSGALE00010098228;PHACTR1;gene:ENSGALG00010010146	intragenic_variant;intron_variant;non_coding_transcript_variant
2	93938106	2_93938106	MODIFIER	intron_variant	GENE_ENSGALE00010018306;GENE_ENSGALE00010018311	ENSGALE00010018306;ENSGALE00010018311	intron_variant
3	45157581	3_45157581	MODIFIER	downstream_gene_variant	ENSGALG00010006318;ENSGALG00010006339;GENE_ENSGALE00010061414;GENE_ENSGALE00010061589;gene:ENSGALG00010006318;gene:ENSGALG00010006339	ENSGALE00010061414;ENSGALE00010061589;SLC22A1;gene:ENSGALG00010006318;gene:ENSGALG00010006339	downstream_gene_variant;intragenic_variant;intron_variant;non_coding_transcript_variant
3	45157632	3_45157632	MODIFIER	downstream_gene_variant	ENSGALG00010006318;ENSGALG00010006339;GENE_ENSGALE00010061414;GENE_ENSGALE00010061589;gene:ENSGALG00010006318;gene:ENSGALG00010006339	ENSGALE00010061414;ENSGALE00010061589;SLC22A1;gene:ENSGALG00010006318;gene:ENSGALG00010006339	downstream_gene_variant;intragenic_variant;intron_variant;non_coding_transcript_variant
3	45206132	3_45206132	MODIFIER	intron_variant	ENSGALG00010006351;GENE_ENSGALE00010061977;gene:ENSGALG00010006351	ENSGALE00010061977;IGF2R;gene:ENSGALG00010006351	intragenic_variant;intron_variant;non_coding_transcript_variant
3	69045958	3_69045958	MODIFIER	intron_variant	GENE_ENSGALE00010155756	ENSGALE00010155756	intron_variant
4	4358701	4_4358701	MODIFIER	upstream_gene_variant	ENSGALG00010015866;GENE_ENSGALE00010157296;GENE_ENSGALE00010157317;GENE_ENSGALE00010157386;GENE_ENSGALE00010157415;GENE_ENSGALE00010157745;gene:ENSGALG00010015866;gene:ENSGALG00010015895	ARHGEF6;ENSGALE00010157296;ENSGALE00010157317;ENSGALE00010157386;ENSGALE00010157415;ENSGALE00010157745;gene:ENSGALG00010015866;gene:ENSGALG00010015895	downstream_gene_variant;intragenic_variant;intron_variant;non_coding_transcript_variant;upstream_gene_variant

CHR	BP	SNP	MAX IMPACT	TOP ANNOTATION	GENE IDS	GENE NAMES	ALL EFFECTS
4	8841206	4_8841206	MODIFIER	intron_variant	ENSGALG00010019979;GENE_ENSGALE00010201577;gene:ENSGALG00010019979	ENSGALE00010201577;gene:ENSGALG00010019979	intragenic_variant;intron_variant;non_coding_transcript_variant
4	8841620	4_8841620	MODIFIER	intron_variant	ENSGALG00010019979;GENE_ENSGALE00010201577;gene:ENSGALG00010019979	ENSGALE00010201577;gene:ENSGALG00010019979	intragenic_variant;intron_variant;non_coding_transcript_variant
4	8841631	4_8841631	MODIFIER	intron_variant	ENSGALG00010019979;GENE_ENSGALE00010201577;gene:ENSGALG00010019979	ENSGALE00010201577;gene:ENSGALG00010019979	intragenic_variant;intron_variant;non_coding_transcript_variant
4	8905235	4_8905235	MODIFIER	intron_variant	ENSGALG00010015449;GENE_ENSGALE00010152631;GENE_ENSGALE00010152654;gene:ENSGALG00010015449	ENSGALE00010152631;ENSGALE00010152654;HDX;gene:ENSGALG00010015449	intragenic_variant;intron_variant;non_coding_transcript_variant
4	8920040	4_8920040	MODIFIER	upstream_gene_variant	ENSGALG00010015449;GENE_ENSGALE00010152631;GENE_ENSGALE00010152654;GENE_ENSGALE00010197467;gene:ENSGALG00010015449	ENSGALE00010152631;ENSGALE00010152654;ENSGALE00010197467;HDX;gene:ENSGALG00010015449	intragenic_variant;intron_variant;non_coding_transcript_variant;upstream_gene_variant
4	8932225	4_8932225	MODIFIER	downstream_gene_variant	GENE_ENSGALE00010197467;GENE_ENSGALE00010197477;GENE_ENSGALE00010197477-GENE_ENSGALE00010153026	ENSGALE00010197467;ENSGALE00010197477;ENSGALE00010197477-ENSGALE00010153026	downstream_gene_variant;intergenic_region
4	9004661	4_9004661	MODIFIER	intergenic_region	GENE_ENSGALE00010197516-ENSGALG00010019346	ENSGALE00010197516-POU3F4	intergenic_region
4	30502663	4_30502663	MODIFIER	downstream_gene_variant	ENSGALG00010006323;GENE_ENSGALE00010061461;GENE_ENSGALE00010061493;GENE_ENSGALE00010061505;GENE_ENSGALE00010061515;GENE_ENSGALE00010061523;GENE_ENSGALE00010061528;GENE_ENSGALE00010061541;GENE_ENSGALE00010061556;GENE_ENSGALE00010061596;gene:ENSGALG00010006323;gene:ENSGALG00010006341	ANAPC10;ENSGALE00010061461;ENSGALE00010061493;ENSGALE00010061505;ENSGALE00010061515;ENSGALE00010061523;ENSGALE00010061528;ENSGALE00010061541;ENSGALE00010061556;ENSGALE00010061596;gene:ENSGALG00010006323;gene:ENSGALG00010006341	downstream_gene_variant;intragenic_variant;intron_variant;non_coding_transcript_variant
4	46640140	4_46640140	MODIFIER	intergenic_region	GENE_ENSGALE00010043391-GENE_ENSGALE00010045700	ENSGALE00010043391-ENSGALE00010045700	intergenic_region
4	50856699	4_50856699	MODIFIER	intergenic_region	ENSGALG00010005178-GENE_ENSGALE00010054454	EPHA5-ENSGALE00010054454	intergenic_region
4	50922179	4_50922179	MODIFIER	upstream_gene_variant	GENE_ENSGALE00010055441;GENE_ENSGALE00010055454;GENE_ENSGALE00010055531;GENE_ENSGALE00010055540;GENE_ENSGALE00010055543;GENE_ENSGALE00010055549;GENE_ENSGALE00010055550	ENSGALE00010055441;ENSGALE00010055454;ENSGALE00010055531;ENSGALE00010055540;ENSGALE00010055543;ENSGALE00010055549;ENSGALE00010055550	downstream_gene_variant;intron_variant;upstream_gene_variant
4	50924668	4_50924668	MODIFIER	intron_variant	GENE_ENSGALE00010055441;GENE_ENSGALE00010055454;GENE_ENSGALE00010055540;GENE_ENSGALE00010055549;GENE_ENSGALE00010055550	ENSGALE00010055441;ENSGALE00010055454;ENSGALE00010055540;ENSGALE00010055549;ENSGALE00010055550	intron_variant;non_coding_transcript_exon_variant
4	50926661	4_50926661	MODIFIER	intron_variant	GENE_ENSGALE00010055441;GENE_ENSGALE00010055454;GENE_ENSGALE00010055540;GENE_ENSGALE00010055549;GENE_ENSGALE00010055550	ENSGALE00010055441;ENSGALE00010055454;ENSGALE00010055540;ENSGALE00010055549;ENSGALE00010055550	intron_variant;non_coding_transcript_exon_variant
4	50931021	4_50931021	MODIFIER	downstream_gene_variant	GENE_ENSGALE00010055441;GENE_ENSGALE00010055454;GENE_ENSGALE00010055540;GENE_ENSGALE00010055549;GENE_ENSGALE00010055550	ENSGALE00010055441;ENSGALE00010055454;ENSGALE00010055540;ENSGALE00010055549;ENSGALE00010055550	downstream_gene_variant;intron_variant
4	50960354	4_50960354	MODIFIER	intron_variant	GENE_ENSGALE00010054481;GENE_ENSGALE00010055441;GENE_ENSGALE00010055454	ENSGALE00010054481;ENSGALE00010055441;ENSGALE00010055454	intron_variant
4	51236931	4_51236931	MODIFIER	intron_variant	ENSGALG00010004924;GENE_ENSGALE00010045798;gene:ENSGALG00010004924	ENSGALE00010045798;TMPRSS11F;gene:ENSGALG00010004924	intragenic_variant;intron_variant;non_coding_transcript_variant
4	51317483	4_51317483	MODIFIER	intergenic_region	GENE_ENSGALE00010054512-GENE_ENSGALE00010054532	ENSGALE00010054512-ENSGALE00010054532	intergenic_region
4	52378327	4_52378327	MODIFIER	intergenic_region	GENE_ENSGALE00010054632-ENSGALG00010004095	ENSGALE00010054632-ANKRD50	intergenic_region

CHR	BP	SNP	MAX IMPACT	TOP ANNOTATION	GENE IDS	GENE NAMES	ALL EFFECTS
4	52426323	4_52426323	MODERATE	missense_variant	ENSGALG00010004095;GENE_ENSGALE00010038198;GENE_ENSGALE00010054661;GENE_ENSGALE00010054669;gene:ENSGALG00010004095	ANKRD50;ENSGALE00010038198;ENSGALE00010054661;ENSGALE00010054669;gene:ENSGALG00010004095	intragenic_variant;missense_variant;non_coding_transcript_variant;upstream_gene_variant
4	52438311	4_52438311	MODIFIER	upstream_gene_variant	GENE_ENSGALE00010054661-GENE_ENSGALE00010055786;GENE_ENSGALE00010055786;GENE_ENSGALE00010055799;GENE_ENSGALE00010055815	ENSGALE00010054661-ENSGALE00010055786;ENSGALE00010055786;ENSGALE00010055799;ENSGALE00010055815	intergenic_region;upstream_gene_variant
4	52438385	4_52438385	MODIFIER	non_coding_transcript_exon_variant	GENE_ENSGALE00010055786;GENE_ENSGALE00010055799;GENE_ENSGALE00010055815	ENSGALE00010055786;ENSGALE00010055799;ENSGALE00010055815	non_coding_transcript_exon_variant
4	52438421	4_52438421	LOW	splice_region_variant&non_coding_transcript_exon_variant	GENE_ENSGALE00010055786;GENE_ENSGALE00010055799;GENE_ENSGALE00010055815	ENSGALE00010055786;ENSGALE00010055799;ENSGALE00010055815	splice_region_variant&non_coding_transcript_exon_variant
4	52438433	4_52438433	MODIFIER	intron_variant	GENE_ENSGALE00010055786;GENE_ENSGALE00010055799;GENE_ENSGALE00010055815	ENSGALE00010055786;ENSGALE00010055799;ENSGALE00010055815	intron_variant
4	52439598	4_52439598	MODIFIER	intron_variant	GENE_ENSGALE00010055786;GENE_ENSGALE00010055799;GENE_ENSGALE00010055815	ENSGALE00010055786;ENSGALE00010055799;ENSGALE00010055815	intron_variant
4	52451790	4_52451790	MODIFIER	intron_variant	GENE_ENSGALE00010055786;GENE_ENSGALE00010055799;GENE_ENSGALE00010055815	ENSGALE00010055786;ENSGALE00010055799;ENSGALE00010055815	intron_variant
4	52464126	4_52464126	MODIFIER	intron_variant	GENE_ENSGALE00010055786;GENE_ENSGALE00010055799;GENE_ENSGALE00010055815	ENSGALE00010055786;ENSGALE00010055799;ENSGALE00010055815	intron_variant
4	52467216	4_52467216	MODIFIER	downstream_gene_variant	GENE_ENSGALE00010054677;GENE_ENSGALE00010055786;GENE_ENSGALE00010055799;GENE_ENSGALE00010055815	ENSGALE00010054677;ENSGALE00010055786;ENSGALE00010055799;ENSGALE00010055815	downstream_gene_variant;intron_variant
4	52474334	4_52474334	MODIFIER	downstream_gene_variant	GENE_ENSGALE00010054677;GENE_ENSGALE00010054697;GENE_ENSGALE00010055786;GENE_ENSGALE00010055799;GENE_ENSGALE00010055815	ENSGALE00010054677;ENSGALE00010054697;ENSGALE00010055786;ENSGALE00010055799;ENSGALE00010055815	downstream_gene_variant;intron_variant
4	52474918	4_52474918	MODIFIER	downstream_gene_variant	GENE_ENSGALE00010054677;GENE_ENSGALE00010054697;GENE_ENSGALE00010055786;GENE_ENSGALE00010055799;GENE_ENSGALE00010055815	ENSGALE00010054677;ENSGALE00010054697;ENSGALE00010055786;ENSGALE00010055799;ENSGALE00010055815	downstream_gene_variant;intron_variant
4	52475158	4_52475158	MODIFIER	downstream_gene_variant	GENE_ENSGALE00010054677;GENE_ENSGALE00010054697;GENE_ENSGALE00010055786;GENE_ENSGALE00010055799;GENE_ENSGALE00010055815	ENSGALE00010054677;ENSGALE00010054697;ENSGALE00010055786;ENSGALE00010055799;ENSGALE00010055815	downstream_gene_variant;intron_variant
4	52476344	4_52476344	MODIFIER	intron_variant	GENE_ENSGALE00010054677;GENE_ENSGALE00010054697;GENE_ENSGALE00010055786;GENE_ENSGALE00010055799;GENE_ENSGALE00010055815	ENSGALE00010054677;ENSGALE00010054697;ENSGALE00010055786;ENSGALE00010055799;ENSGALE00010055815	intron_variant;non_coding_transcript_exon_variant
4	52479037	4_52479037	MODIFIER	upstream_gene_variant	GENE_ENSGALE00010054677;GENE_ENSGALE00010054697;GENE_ENSGALE00010055786;GENE_ENSGALE00010055799;GENE_ENSGALE00010055815	ENSGALE00010054677;ENSGALE00010054697;ENSGALE00010055786;ENSGALE00010055799;ENSGALE00010055815	intron_variant;non_coding_transcript_exon_variant;upstream_gene_variant
4	52481561	4_52481561	MODIFIER	upstream_gene_variant	GENE_ENSGALE00010054677;GENE_ENSGALE00010054697;GENE_ENSGALE00010055786;GENE_ENSGALE00010055799;GENE_ENSGALE00010055815	ENSGALE00010054677;ENSGALE00010054697;ENSGALE00010055786;ENSGALE00010055799;ENSGALE00010055815	downstream_gene_variant;intron_variant;upstream_gene_variant
4	52482069	4_52482069	MODIFIER	upstream_gene_variant	GENE_ENSGALE00010054677;GENE_ENSGALE00010055786;GENE_ENSGALE00010055799;GENE_ENSGALE00010055815	ENSGALE00010054677;ENSGALE00010055786;ENSGALE00010055799;ENSGALE00010055815	downstream_gene_variant;intron_variant;upstream_gene_variant
4	52482110	4_52482110	MODIFIER	upstream_gene_variant	GENE_ENSGALE00010054677;GENE_ENSGALE00010055786;GENE_ENSGALE00010055799;GENE_ENSGALE00010055815	ENSGALE00010054677;ENSGALE00010055786;ENSGALE00010055799;ENSGALE00010055815	downstream_gene_variant;intron_variant;upstream_gene_variant
4	52488692	4_52488692	MODIFIER	downstream_gene_variant	GENE_ENSGALE00010055799;GENE_ENSGALE00010055799-GENE_ENSGALE00010054715	ENSGALE00010055799;ENSGALE00010055799-ENSGALE00010054715	downstream_gene_variant;intergenic_region

CHR	BP	SNP	MAX IMPACT	TOP ANNOTATION	GENE IDS	GENE NAMES	ALL EFFECTS
4	52489714	4_52489714	MODIFIER	downstream_gene_variant	GENE_ENSGALE00010055799;GENE_ENSGALE00010055799-GENE_ENSGALE00010054715	ENSGALE00010055799;ENSGALE00010055799-ENSGALE00010054715	downstream_gene_variant;intergenic_region
4	52490844	4_52490844	MODIFIER	downstream_gene_variant	GENE_ENSGALE00010055799;GENE_ENSGALE00010055799-GENE_ENSGALE00010054715	ENSGALE00010055799;ENSGALE00010055799-ENSGALE00010054715	downstream_gene_variant;intergenic_region
4	52491382	4_52491382	MODIFIER	intergenic_region	GENE_ENSGALE00010055799-GENE_ENSGALE00010054715	ENSGALE00010055799-ENSGALE00010054715	intergenic_region
4	52491401	4_52491401	MODIFIER	intergenic_region	GENE_ENSGALE00010055799-GENE_ENSGALE00010054715	ENSGALE00010055799-ENSGALE00010054715	intergenic_region
4	52491727	4_52491727	MODIFIER	intergenic_region	GENE_ENSGALE00010055799-GENE_ENSGALE00010054715	ENSGALE00010055799-ENSGALE00010054715	intergenic_region
4	52492921	4_52492921	MODIFIER	intergenic_region	GENE_ENSGALE00010055799-GENE_ENSGALE00010054715	ENSGALE00010055799-ENSGALE00010054715	intergenic_region
4	52498509	4_52498509	MODIFIER	intergenic_region	GENE_ENSGALE00010055799-GENE_ENSGALE00010054715	ENSGALE00010055799-ENSGALE00010054715	intergenic_region
4	52498560	4_52498560	MODIFIER	intergenic_region	GENE_ENSGALE00010055799-GENE_ENSGALE00010054715	ENSGALE00010055799-ENSGALE00010054715	intergenic_region
4	52498593	4_52498593	MODIFIER	intergenic_region	GENE_ENSGALE00010055799-GENE_ENSGALE00010054715	ENSGALE00010055799-ENSGALE00010054715	intergenic_region
4	52499329	4_52499329	MODIFIER	intergenic_region	GENE_ENSGALE00010055799-GENE_ENSGALE00010054715	ENSGALE00010055799-ENSGALE00010054715	intergenic_region
4	52663746	4_52663746	MODIFIER	intron_variant	GENE_ENSGALE00010055911;GENE_ENSGALE00010055928	ENSGALE00010055911;ENSGALE00010055928	intron_variant
4	56500442	4_56500442	MODIFIER	intron_variant	ENSGALG00010003978;GENE_ENSGALE00010036940;GENE_ENSGALE00010036979;gene:ENSGALG00010003978	ANK2;ENSGALE00010036940;ENSGALE00010036979;gene:ENSGALG00010003978	intragenic_variant;intron_variant;non_coding_transcript_variant
4	56605141	4_56605141	MODIFIER	downstream_gene_variant	ENSGALG00010004001;GENE_ENSGALE00010037176;GENE_ENSGALE00010037201;GENE_ENSGALE00010057875;GENE_ENSGALE00010057900;GENE_ENSGALE00010057916;GENE_ENSGALE00010057939;GENE_ENSGALE00010057972;GENE_ENSGALE00010057999;gene:ENSGALG00010004001;gene:ENSGALG00010005945;gene:ENSGALG00010005950;gene:ENSGALG00010005953;gene:ENSGALG00010005958;gene:ENSGALG00010005968;gene:ENSGALG00010005971	ENSGALE00010037176;ENSGALE00010037201;ENSGALE00010057875;ENSGALE00010057900;ENSGALE00010057916;ENSGALE00010057939;ENSGALE00010057972;ENSGALE00010057999;LARP7;gene:ENSGALG00010004001;gene:ENSGALG00010005945;gene:ENSGALG00010005950;gene:ENSGALG00010005953;gene:ENSGALG00010005958;gene:ENSGALG00010005968;gene:ENSGALG00010005971	downstream_gene_variant;intragenic_variant;intron_variant;non_coding_transcript_variant
4	58233741	4_58233741	MODIFIER	intron_variant	ENSGALG00010004366;GENE_ENSGALE00010041035;GENE_ENSGALE00010041084;gene:ENSGALG00010004366	ENSGALE00010041035;ENSGALE00010041084;UNC5C;gene:ENSGALG00010004366	intragenic_variant;intron_variant;non_coding_transcript_variant
4	58592454	4_58592454	MODIFIER	intergenic_region	GENE_ENSGALE00010057452-GENE_ENSGALE00010057523	ENSGALE00010057452-ENSGALE00010057523	intergenic_region
4	58890405	4_58890405	MODIFIER	upstream_gene_variant	GENE_ENSGALE00010055155;GENE_ENSGALE00010057523;GENE_ENSGALE00010057654;GENE_ENSGALE00010057679;GENE_ENSGALE00010057692	ENSGALE00010055155;ENSGALE00010057523;ENSGALE00010057654;ENSGALE00010057679;ENSGALE00010057692	intron_variant;upstream_gene_variant
4	59094671	4_59094671	MODIFIER	intron_variant	GENE_ENSGALE00010057654;GENE_ENSGALE00010057679;GENE_ENSGALE00010057692	ENSGALE00010057654;ENSGALE00010057679;ENSGALE00010057692	intron_variant
4	59165705	4_59165705	MODIFIER	intron_variant	GENE_ENSGALE00010057654;GENE_ENSGALE00010057679;GENE_ENSGALE00010057692	ENSGALE00010057654;ENSGALE00010057679;ENSGALE00010057692	intron_variant
4	59176477	4_59176477	MODIFIER	intron_variant	GENE_ENSGALE00010057654;GENE_ENSGALE00010057679;GENE_ENSGALE00010057692	ENSGALE00010057654;ENSGALE00010057679;ENSGALE00010057692	intron_variant

CHR	BP	SNP	MAX IMPACT	TOP ANNOTATION	GENE IDS	GENE NAMES	ALL EFFECTS
4	59178040	4_59178040	MODIFIER	intron_variant	GENE_ENSGALE00010057654;GENE_ENSGALE00010057679;GENE_ENSGALE00010057692	ENSGALE00010057654;ENSGALE00010057679;ENSGALE00010057692	intron_variant
4	59470884	4_59470884	MODIFIER	intergenic_region	ENSGALG00010004641-ENSGALG00010004716	METAP1-ADH6	intergenic_region
4	59483846	4_59483846	LOW	synonymous_variant	ENSGALG00010004716;GENE_ENSGALE00010043746;GENE_ENSGALE00010043789;gene:ENSGALG00010004716	ADH6;ENSGALE00010043746;ENSGALE00010043789;gene:ENSGALG00010004716	intragenic_variant;intron_variant;non_coding_transcript_variant;synonymous_variant
4	59544379	4_59544379	MODIFIER	3_prime_UTR_variant	ENSGALG00010005241;ENSGALG00010005268;GENE_ENSGALE00010049451;GENE_ENSGALE00010049457;GENE_ENSGALE00010049824;gene:ENSGALG00010005241;gene:ENSGALG00010005268	C4H4orf54;ENSGALE00010049451;ENSGALE00010049457;ENSGALE00010049824;MTTP;gene:ENSGALG00010005241;gene:ENSGALG00010005268	3_prime_UTR_variant;intragenic_variant;intron_variant;non_coding_transcript_variant
4	59544384	4_59544384	MODIFIER	3_prime_UTR_variant	ENSGALG00010005241;ENSGALG00010005268;GENE_ENSGALE00010049451;GENE_ENSGALE00010049457;GENE_ENSGALE00010049824;gene:ENSGALG00010005241;gene:ENSGALG00010005268	C4H4orf54;ENSGALE00010049451;ENSGALE00010049457;ENSGALE00010049824;MTTP;gene:ENSGALG00010005241;gene:ENSGALG00010005268	3_prime_UTR_variant;intragenic_variant;intron_variant;non_coding_transcript_variant
5	20885150	5_20885150	MODIFIER	intron_variant	GENE_ENSGALE00010246276;GENE_ENSGALE00010246293;GENE_ENSGALE00010246313;GENE_ENSGALE00010246335;GENE_ENSGALE00010246420	ENSGALE00010246276;ENSGALE00010246293;ENSGALE00010246313;ENSGALE00010246335;ENSGALE00010246420	intron_variant
5	26207271	5_26207271	MODIFIER	upstream_gene_variant	ENSGALG00010021257;GENE_ENSGALE00010217682;GENE_ENSGALE00010217685;GENE_ENSGALE00010217780;GENE_ENSGALE00010217789;GENE_ENSGALE00010217796;GENE_ENSGALE00010245609;gene:ENSGALG00010021257	ENSGALE00010217682;ENSGALE00010217685;ENSGALE00010217780;ENSGALE00010217789;ENSGALE00010217796;ENSGALE00010245609;RGS6;gene:ENSGALG00010021257	downstream_gene_variant;intragenic_variant;intron_variant;non_coding_transcript_variant;upstream_gene_variant
5	49728859	5_49728859	MODIFIER	downstream_gene_variant	ENSGALG00010015759;GENE_ENSGALE00010156179;GENE_ENSGALE00010187204;gene:ENSGALG00010015759;gene:ENSGALG00010018475	EIF5;ENSGALE00010156179;ENSGALE00010187204;gene:ENSGALG00010015759;gene:ENSGALG00010018475	downstream_gene_variant;intragenic_variant;intron_variant;non_coding_transcript_variant
5	57964781	5_57964781	MODIFIER	downstream_gene_variant	ENSGALG00010018032;GENE_ENSGALE00010181238;GENE_ENSGALE00010182068;GENE_ENSGALE00010182081;GENE_ENSGALE00010182103;GENE_ENSGALE00010182123;gene:ENSGALG00010017976;gene:ENSGALG00010018032	ENSGALE00010181238;ENSGALE00010182068;ENSGALE00010182081;ENSGALE00010182103;ENSGALE00010182123;ERO1A;gene:ENSGALG00010017976;gene:ENSGALG00010018032	downstream_gene_variant;intragenic_variant;intron_variant;non_coding_transcript_variant
6	2869816	6_2869816	MODIFIER	intergenic_region	GENE_ENSGALE00010108921-GENE_ENSGALE00010109995	ENSGALE00010108921-ENSGALE00010109995	intergenic_region
6	3146546	6_3146546	MODIFIER	intron_variant	ENSGALG00010010438;GENE_ENSGALE00010101343;GENE_ENSGALE00010101439;gene:ENSGALG00010010438	ENSGALE00010101343;ENSGALE00010101439;LDB3;gene:ENSGALG00010010438	intragenic_variant;intron_variant;non_coding_transcript_variant
6	3146578	6_3146578	MODIFIER	intron_variant	ENSGALG00010010438;GENE_ENSGALE00010101343;GENE_ENSGALE00010101439;gene:ENSGALG00010010438	ENSGALE00010101343;ENSGALE00010101439;LDB3;gene:ENSGALG00010010438	intragenic_variant;intron_variant;non_coding_transcript_variant
6	3172247	6_3172247	MODIFIER	intron_variant	ENSGALG00010010438;GENE_ENSGALE00010101343;GENE_ENSGALE00010101439;gene:ENSGALG00010010438	ENSGALE00010101343;ENSGALE00010101439;LDB3;gene:ENSGALG00010010438	intragenic_variant;intron_variant;non_coding_transcript_variant
6	3172268	6_3172268	MODIFIER	intron_variant	ENSGALG00010010438;GENE_ENSGALE00010101343;GENE_ENSGALE00010101439;gene:ENSGALG00010010438	ENSGALE00010101343;ENSGALE00010101439;LDB3;gene:ENSGALG00010010438	intragenic_variant;intron_variant;non_coding_transcript_variant
8	15513773	8_15513773	MODIFIER	intron_variant	GENE_ENSGALE00010255807;GENE_ENSGALE00010255836	ENSGALE00010255807;ENSGALE00010255836	intron_variant
8	15579761	8_15579761	MODIFIER	intron_variant	ENSGALG00010023030;GENE_ENSGALE00010239521;gene:ENSGALG00010023030	ENSGALE00010239521;HS2ST1;gene:ENSGALG00010023030	intragenic_variant;intron_variant;non_coding_transcript_variant
8	15638386	8_15638386	MODIFIER	intron_variant	ENSGALG00010023059;GENE_ENSGALE00010239849;GENE_ENSGALE00010239883;GENE_ENSGALE00010239934;gene:ENSGALG00010023059	ENSGALE00010239849;ENSGALE00010239883;ENSGALE00010239934;SELENOF;gene:ENSGALG00010023059	intragenic_variant;intron_variant;non_coding_transcript_variant

CHR	BP	SNP	MAX IMPACT	TOP ANNOTATION	GENE IDS	GENE NAMES	ALL EFFECTS
8	15690023	8_15690023	MODIFIER	intron_variant	ENSGALG00010023101;GENE_ENSGALE00010240550;gene:ENSGALG00010023101	CLCA2;ENSGALE00010240550;gene:ENSGALG00010023101	intragenic_variant;intron_variant;non_coding_transcript_variant
8	15718134	8_15718134	MODIFIER	upstream_gene_variant	ENSGALG00010023101-ENSGALG00010023161;GENE_ENSGALE00010240473;GENE_ENSGALE00010241342;GENE_ENSGALE00010241369;GENE_ENSGALE00010241414;GENE_ENSGALE00010241470;gene:ENSGALG00010023101;gene:ENSGALG00010023161	CLCA2-ODF2L;ENSGALE00010240473;ENSGALE00010241342;ENSGALE00010241369;ENSGALE00010241414;ENSGALE00010241470;gene:ENSGALG00010023101;gene:ENSGALG00010023161	downstream_gene_variant;intergenic_region;upstream_gene_variant
8	15795166	8_15795166	MODIFIER	intron_variant	ENSGALG00010023195;GENE_ENSGALE00010241822;GENE_ENSGALE00010241921;GENE_ENSGALE00010242085;gene:ENSGALG00010023195	COL24A1;ENSGALE00010241822;ENSGALE00010241921;ENSGALE00010242085;gene:ENSGALG00010023195	intragenic_variant;intron_variant;non_coding_transcript_variant
9	19567769	9_19567769	MODIFIER	3_prime_UTR_variant	ENSGALG00010016761;GENE_ENSGALE00010166312;GENE_ENSGALE00010166365;GENE_ENSGALE00010166441;GENE_ENSGALE00010166590;GENE_ENSGALE00010166604;GENE_ENSGALE00010166618;gene:ENSGALG00010016733;gene:ENSGALG00010016761	ENSGALE00010166312;ENSGALE00010166365;ENSGALE00010166441;ENSGALE00010166590;ENSGALE00010166604;ENSGALE00010166618;GPR160;gene:ENSGALG00010016733;gene:ENSGALG00010016761	3_prime_UTR_variant;downstream_gene_variant;intragenic_variant;intron_variant;non_coding_transcript_variant
9	20591655	9_20591655	MODIFIER	intron_variant	ENSGALG00010001267;GENE_ENSGALE00010010995;GENE_ENSGALE00010010998;gene:ENSGALG00010001267	BCHE;ENSGALE00010010995;ENSGALE00010010998;gene:ENSGALG00010001267	intragenic_variant;intron_variant;non_coding_transcript_variant
9	20921150	9_20921150	MODIFIER	upstream_gene_variant	ENSGALG00010001273-GENE_ENSGALE00010014369;GENE_ENSGALE00010014369;GENE_ENSGALE00010014448	ENSGALE00010014369;ENSGALE00010014448;SI-ENSGALE00010014369	downstream_gene_variant;intergenic_region;upstream_gene_variant
9	20931265	9_20931265	MODIFIER	downstream_gene_variant	GENE_ENSGALE00010014017;GENE_ENSGALE00010014369;GENE_ENSGALE00010014448	ENSGALE00010014017;ENSGALE00010014369;ENSGALE00010014448	downstream_gene_variant;intron_variant
9	20976534	9_20976534	MODIFIER	intron_variant	GENE_ENSGALE00010014396;GENE_ENSGALE00010014448;GENE_ENSGALE00010014450	ENSGALE00010014396;ENSGALE00010014448;ENSGALE00010014450	intron_variant
9	21230441	9_21230441	MODIFIER	intergenic_region	GENE_ENSGALE00010014448-ENSGALG00010001137	ENSGALE00010014448-OTOL1	intergenic_region
9	21248107	9_21248107	MODIFIER	intergenic_region	GENE_ENSGALE00010014448-ENSGALG00010001137	ENSGALE00010014448-OTOL1	intergenic_region
10	3508373	10_3508373	MODIFIER	intron_variant	ENSGALG00010024121;GENE_ENSGALE00010252628;gene:ENSGALG00010024121	ENSGALE00010252628;NRG4;gene:ENSGALG00010024121	intragenic_variant;intron_variant;non_coding_transcript_variant
10	15917001	10_15917001	MODIFIER	intergenic_region	ENSGALG00010016558-GENE_ENSGALE00010208035	NR2F2-ENSGALE00010208035	intergenic_region
11	7373893	11_7373893	MODIFIER	3_prime_UTR_variant	ENSGALG00010023405;ENSGALG00010023513;GENE_ENSGALE00010244642;GENE_ENSGALE00010244670;GENE_ENSGALE00010245532;GENE_ENSGALE00010245578;GENE_ENSGALE00010245588;GENE_ENSGALE00010245627;gene:ENSGALG00010023405;gene:ENSGALG00010023513	CDCA9;DNAJA2;ENSGALE00010244642;ENSGALE00010244670;ENSGALE00010245532;ENSGALE00010245578;ENSGALE00010245588;ENSGALE00010245627;gene:ENSGALG00010023405;gene:ENSGALG00010023513	3_prime_UTR_variant;downstream_gene_variant;intragenic_variant;intron_variant;non_coding_transcript_variant;upstream_gene_variant
11	7373963	11_7373963	MODIFIER	3_prime_UTR_variant	ENSGALG00010023405;ENSGALG00010023513;GENE_ENSGALE00010244642;GENE_ENSGALE00010244670;GENE_ENSGALE00010245532;GENE_ENSGALE00010245578;GENE_ENSGALE00010245588;GENE_ENSGALE00010245627;gene:ENSGALG00010023405;gene:ENSGALG00010023513	CDCA9;DNAJA2;ENSGALE00010244642;ENSGALE00010244670;ENSGALE00010245532;ENSGALE00010245578;ENSGALE00010245588;ENSGALE00010245627;gene:ENSGALG00010023405;gene:ENSGALG00010023513	3_prime_UTR_variant;downstream_gene_variant;intragenic_variant;intron_variant;non_coding_transcript_variant;upstream_gene_variant
11	7374024	11_7374024	MODIFIER	3_prime_UTR_variant	ENSGALG00010023405;ENSGALG00010023513;GENE_ENSGALE00010244642;GENE_ENSGALE00010244670;GENE_ENSGALE00010245532;GENE_ENSGALE00010245578;GENE_ENSGALE00010245588;GENE_	CDCA9;DNAJA2;ENSGALE00010244642;ENSGALE00010244670;ENSGALE00010245532;ENSGALE00010245578;ENSGALE00010245588;ENSGALE00010245627;gene:ENSGALG00010023405;gene:ENSGALG00010023513	3_prime_UTR_variant;downstream_gene_variant;intragenic_variant;intron_variant;non_coding_transcript_variant;upstream_gene_variant

CHR	BP	SNP	MAX IMPACT	TOP ANNOTATION	GENE IDS	GENE NAMES	ALL EFFECTS
					ENSGALE00010245627;gene:ENSGALG00010023405;gene:ENSGALG00010023513		
11	7614407	11_7614407	MODIFIER	intergenic_region	GENE_ENSGALE00010269417-GENE_ENSGALE00010271178	ENSGALE00010269417-ENSGALE00010271178	intergenic_region
17	10307375	17_10307375	MODIFIER	intron_variant	ENSGALG00010027399;GENE_ENSGALE00010286773;GENE_ENSGALE00010286777;gene:ENSGALG00010027399	ENSGALE00010286773;ENSGALE00010286777;MAPKAP1;gene:ENSGALG00010027399	intragenic_variant;intron_variant;non_coding_transcript_variant
17	10318885	17_10318885	MODIFIER	intron_variant	ENSGALG00010027399;GENE_ENSGALE00010286773;GENE_ENSGALE00010286777;gene:ENSGALG00010027399	ENSGALE00010286773;ENSGALE00010286777;MAPKAP1;gene:ENSGALG00010027399	intragenic_variant;intron_variant;non_coding_transcript_variant
17	10368305	17_10368305	MODIFIER	intron_variant	ENSGALG00010027414;GENE_ENSGALE00010286924;GENE_ENSGALE00010286934;gene:ENSGALG00010027414	ENSGALE00010286924;ENSGALE00010286934;PBX3;gene:ENSGALG00010027414	intragenic_variant;intron_variant;non_coding_transcript_variant
17	10404459	17_10404459	MODIFIER	intron_variant	ENSGALG00010027414;GENE_ENSGALE00010286924;GENE_ENSGALE00010286934;gene:ENSGALG00010027414	ENSGALE00010286924;ENSGALE00010286934;PBX3;gene:ENSGALG00010027414	intragenic_variant;intron_variant;non_coding_transcript_variant
17	10404889	17_10404889	MODIFIER	intron_variant	ENSGALG00010027414;GENE_ENSGALE00010286924;GENE_ENSGALE00010286934;gene:ENSGALG00010027414	ENSGALE00010286924;ENSGALE00010286934;PBX3;gene:ENSGALG00010027414	intragenic_variant;intron_variant;non_coding_transcript_variant
17	10405077	17_10405077	MODIFIER	intron_variant	ENSGALG00010027414;GENE_ENSGALE00010286924;GENE_ENSGALE00010286934;gene:ENSGALG00010027414	ENSGALE00010286924;ENSGALE00010286934;PBX3;gene:ENSGALG00010027414	intragenic_variant;intron_variant;non_coding_transcript_variant
17	10503767	17_10503767	MODIFIER	intergenic_region	ENSGALG00010027414-GENE_ENSGALE00010290401	PBX3-ENSGALE00010290401	intergenic_region
17	10509545	17_10509545	MODIFIER	intergenic_region	ENSGALG00010027414-GENE_ENSGALE00010290401	PBX3-ENSGALE00010290401	intergenic_region
18	2601049	18_2601049	MODIFIER	intron_variant	ENSGALG00010029363;GENE_ENSGALE00010307302;GENE_ENSGALE00010307305;gene:ENSGALG00010029363	ENSGALE00010307302;ENSGALE00010307305;PIK3R6;gene:ENSGALG00010029363	intragenic_variant;intron_variant;non_coding_transcript_variant
18	2602079	18_2602079	MODIFIER	intron_variant	ENSGALG00010029363;GENE_ENSGALE00010307302;GENE_ENSGALE00010307305;gene:ENSGALG00010029363	ENSGALE00010307302;ENSGALE00010307305;PIK3R6;gene:ENSGALG00010029363	intragenic_variant;intron_variant;non_coding_transcript_variant
18	2602114	18_2602114	MODIFIER	intron_variant	ENSGALG00010029363;GENE_ENSGALE00010307302;GENE_ENSGALE00010307305;gene:ENSGALG00010029363	ENSGALE00010307302;ENSGALE00010307305;PIK3R6;gene:ENSGALG00010029363	intragenic_variant;intron_variant;non_coding_transcript_variant
25	566663	25_566663	MODIFIER	intergenic_region	ENSGALG00010027354-GENE_ENSGALE00010301425	gene:ENSGALG00010027354-ENSGALE00010301425	intergenic_region
25	566688	25_566688	MODIFIER	intergenic_region	ENSGALG00010027354-GENE_ENSGALE00010301425	gene:ENSGALG00010027354-ENSGALE00010301425	intergenic_region
28	521922	28_521922	MODIFIER	intron_variant	ENSGALG00010028283;GENE_ENSGALE00010296509;GENE_ENSGALE00010296515;GENE_ENSGALE00010296517;GENE_ENSGALE00010296522;gene:ENSGALG00010028283	CERS4;ENSGALE00010296509;ENSGALE00010296515;ENSGALE00010296517;ENSGALE00010296522;gene:ENSGALG00010028283	intragenic_variant;intron_variant;non_coding_transcript_variant
28	521968	28_521968	MODIFIER	intron_variant	ENSGALG00010028283;GENE_ENSGALE00010296509;GENE_ENSGALE00010296515;GENE_ENSGALE00010296517;GENE_ENSGALE00010296522;gene:ENSGALG00010028283	CERS4;ENSGALE00010296509;ENSGALE00010296515;ENSGALE00010296517;ENSGALE00010296522;gene:ENSGALG00010028283	intragenic_variant;intron_variant;non_coding_transcript_variant
Z	24478892	chrZ_24478892	MODIFIER	intron_variant	ENSGALG00010010985;GENE_ENSGALE00010107190;gene:ENSGALG00010010985	COL4A3BP;ENSGALE00010107190;gene:ENSGALG00010010985	intragenic_variant;intron_variant;non_coding_transcript_variant

Table S1 (continued). Part H1. Candidate genes: genomic coordinates and biotypes.

GENE ID	GENE NAME	CHR	START	END	STRAND	BIOTYPE	TRAITS
ENSGALG00010010915	APP	1	102713376	102979763	-	protein_coding	MeL
ENSGALG00010013988	ENSGALG00010013988	1	104001544	104008177	+	lncRNA	MeL
ENSGALG00010005447	MYO3A	2	16209614	16306062	-	protein_coding	MeL
ENSGALG00010006395	ENSGALG00010006395	2	16316947	16342158	-	lncRNA	MeL
ENSGALG00010007319	ATP9B	2	56433404	56601425	-	protein_coding	MeL
ENSGALG00010010146	PHACTR1	2	61968635	62308616	-	protein_coding	MeL
ENSGALG00010002055	ENSGALG00010002055	2	93929025	93946587	+	lncRNA	MeC
ENSGALG00010006318	ENSGALG00010006318	3	45130023	45161912	+	protein_coding	MeC
ENSGALG00010006339	SLC22A1	3	45155902	45168238	-	protein_coding	MeC
ENSGALG00010006351	IGF2R	3	45169354	45223705	-	protein_coding	MeC
ENSGALG00010015726	ENSGALG00010015726	3	68998471	69111901	+	lncRNA	MeL
ENSGALG00010015866	ARHGEF6	4	4329627	4360781	-	protein_coding	MeL
ENSGALG00010019979	ENSGALG00010019979	4	8761775	8863779	-	protein_coding	MeL
ENSGALG00010015449	HDX	4	8888635	8924125	+	protein_coding	MeL
ENSGALG00010019544	ENSGALG00010019544	4	8929554	8931379	+	lncRNA	MeL
ENSGALG00010006323	ANAPC10	4	30412426	30584370	-	protein_coding	MeL
ENSGALG00010004665	ENSGALG00010004665	4	46589750	46593860	-	lncRNA	MeL
ENSGALG00010005718	ENSGALG00010005718	4	50888531	50926810	+	lncRNA	MeL
ENSGALG00010005702	ENSGALG00010005702	4	50895062	51017684	-	lncRNA	MeL
ENSGALG00010005579	ENSGALG00010005579	4	50956889	50976245	+	lncRNA	MeL
ENSGALG00010004924	TMPRSS11F	4	51214879	51259486	-	protein_coding	MeC
ENSGALG00010005582	ENSGALG00010005582	4	51310713	51311843	-	lncRNA	MeC
ENSGALG00010004095	ANKRD50	4	52390954	52426448	+	protein_coding	MeC
ENSGALG00010005750	ENSGALG00010005750	4	52438340	52486173	+	lncRNA	MeC
ENSGALG00010005603	ENSGALG00010005603	4	52472150	52479418	-	lncRNA	MeC
ENSGALG00010005606	ENSGALG00010005606	4	52475211	52476699	-	lncRNA	MeC
ENSGALG00010005766	ENSGALG00010005766	4	52619852	52842411	-	lncRNA	MeC
ENSGALG00010003978	ANK2	4	56240974	56552967	-	protein_coding	MeC
ENSGALG00010004001	LARP7	4	56588026	56615800	-	protein_coding	MeC
ENSGALG00010004366	UNC5C	4	58117403	58357037	-	protein_coding	MeL
ENSGALG00010005897	ENSGALG00010005897	4	58503354	58543604	+	lncRNA	MeL
ENSGALG00010005904	ENSGALG00010005904	4	58660832	58925783	+	lncRNA	MeC
ENSGALG00010005914	ENSGALG00010005914	4	58801245	59181365	-	lncRNA	MeC
ENSGALG00010004716	ADH6	4	59481502	59493307	-	protein_coding	MeC;MeL
ENSGALG00010005241	C4H4orf54	4	59539008	59612363	-	protein_coding	MeC;MeL
ENSGALG00010005268	MTTP	4	59541505	59570552	+	protein_coding	MeC;MeL

GENE ID	GENE NAME	CHR	START	END	STRAND	BIOTYPE	TRAITS
ENSGALG00010023599	ENSGALG00010023599	5	20759263	20937515	+	lncRNA	MeC
ENSGALG00010023610	ENSGALG00010023610	5	20860160	20924999	-	lncRNA	MeC
ENSGALG00010021257	RGS6	5	26202559	26437124	-	protein_coding	MeC
ENSGALG00010015759	EIF5	5	49724445	49731754	+	protein_coding	MeC
ENSGALG00010018032	ERO1A	5	57963933	57982179	-	protein_coding	MeL
ENSGALG00010011180	ENSGALG00010011180	6	2862704	2863990	+	lncRNA	MeL
ENSGALG00010010438	LDB3	6	3072380	3190542	+	protein_coding	MeC
ENSGALG00010024404	ENSGALG00010024404	8	15468257	15542315	-	lncRNA	MeL
ENSGALG00010023030	HS2ST1	8	15559264	15631433	-	protein_coding	MeL
ENSGALG00010023059	SELENOF	8	15631823	15650917	+	protein_coding	MeL
ENSGALG00010023101	CLCA2	8	15684106	15716170	-	protein_coding	MeL
ENSGALG00010023161	ODF2L	8	15719944	15741097	+	protein_coding	MeL
ENSGALG00010023195	COL24A1	8	15769616	15885073	+	protein_coding	MeL
ENSGALG00010016761	GPR160	9	19557000	19573164	-	protein_coding	MeC
ENSGALG00010001267	BCHE	9	20533815	20709958	+	protein_coding	MeL
ENSGALG00010001625	ENSGALG00010001625	9	20921658	20946264	+	lncRNA	MeC
ENSGALG00010001631	ENSGALG00010001631	9	20923467	21073286	-	lncRNA	MeC;MeL
ENSGALG00010001629	ENSGALG00010001629	9	20949667	21008587	+	lncRNA	MeC
ENSGALG00010024121	NRG4	10	3490438	3512279	+	protein_coding	MeL
ENSGALG00010016558	NR2F2	10	15685086	15856587	+	protein_coding	MeL
ENSGALG00010023405	DNAJA2	11	7360707	7374405	+	protein_coding	MeC
ENSGALG00010023513	CDCA9	11	7372868	7380271	-	protein_coding	MeC
ENSGALG00010027399	MAPKAP1	17	10248646	10333782	-	protein_coding	MeL
ENSGALG00010027414	PBX3	17	10335682	10432332	+	protein_coding	MeL
ENSGALG00010029363	PIK3R6	18	2582753	2605437	-	protein_coding	MeL
ENSGALG00010028815	ENSGALG00010028815	25	584448	595470	+	lncRNA	MeL
ENSGALG00010028283	CERS4	28	509337	566508	-	protein_coding	MeC
ENSGALG00010010985	COL4A3BP	Z	24446204	24520373	+	protein_coding	MeL

Table S1 (continued). Part H2. Candidate genes: variant and LD-region evidence.

GENE ID	GENE NAME	STRICT VARIANT EVIDENCE	STRICT VARIANTS	STRICT MAPPING MODE	REGION GENE EVIDENCE	REGION SIGNALS	REGION MAPPING MODE
ENSGALG00010010915	APP	1	1_102807177	variant_gene_overlap	1	MEL_AUTOSOMES_SIG002	ld_interval_overlap
ENSGALG00010013988	ENSGALG00010013988	0			1	MEL_AUTOSOMES_SIG005	nearest_within_500kb
ENSGALG00010005447	MYO3A	1	2_16298204	variant_gene_overlap	1	MEL_AUTOSOMES_SIG013	ld_interval_overlap
ENSGALG00010006395	ENSGALG00010006395	1	2_16313576	variant_nearest_within_5kb	0		
ENSGALG00010007319	ATP9B	1	2_56602236;2_56602250;2_56602264	variant_nearest_within_5kb	1	MEL_AUTOSOMES_SIG025	nearest_within_500kb

GENE ID	GENE NAME	STRICT VARIANT EVIDENCE	STRICT VARIANTS	STRICT MAPPING MODE	REGION GENE EVIDENCE	REGION SIGNALS	REGION MAPPING MODE
ENSGALG00010010146	PHACTR1	1	2_62109190	variant_gene_overlap	1	MEL_AUTOSOMES_SIG022	ld_interval_overlap
ENSGALG00010002055	ENSGALG00010002055	1	2_93938106	variant_gene_overlap	1	MEC_AUTOSOMES_SIG008	ld_interval_overlap
ENSGALG00010006318	ENSGALG00010006318	1	3_45157581;3_45157632	variant_gene_overlap	1	MEC_AUTOSOMES_SIG006	ld_interval_overlap
ENSGALG00010006339	SLC22A1	1	3_45157581;3_45157632	variant_gene_overlap	1	MEC_AUTOSOMES_SIG006	ld_interval_overlap
ENSGALG00010006351	IGF2R	1	3_45206132	variant_gene_overlap	1	MEC_AUTOSOMES_SIG016	ld_interval_overlap
ENSGALG00010015726	ENSGALG00010015726	1	3_69045958	variant_gene_overlap	1	MEL_AUTOSOMES_SIG020	ld_interval_overlap
ENSGALG00010015866	ARHGEF6	1	4_4358701	variant_gene_overlap	1	MEL_AUTOSOMES_SIG024	ld_interval_overlap
ENSGALG00010019979	ENSGALG00010019979	1	4_8841206;4_8841620;4_8841631	variant_gene_overlap	1	MEL_AUTOSOMES_SIG001	ld_interval_overlap
ENSGALG00010015449	HDX	1	4_8905235;4_8920040	variant_gene_overlap	0		
ENSGALG00010019544	ENSGALG00010019544	1	4_8932225	variant_nearest_within_5kb	0		
ENSGALG00010006323	ANAPC10	1	4_30502663	variant_gene_overlap	1	MEL_AUTOSOMES_SIG012	ld_interval_overlap
ENSGALG00010004665	ENSGALG00010004665	0			1	MEL_AUTOSOMES_SIG018	nearest_within_500kb
ENSGALG00010005718	ENSGALG00010005718	1	4_50922179;4_50924668;4_50926661	variant_gene_overlap	1	MEL_AUTOSOMES_SIG003;MEL_AUTOSOMES_SIG017	ld_interval_overlap
ENSGALG00010005702	ENSGALG00010005702	1	4_50922179;4_50924668;4_50926661;4_50931021;4_50960354	variant_gene_overlap	1	MEL_AUTOSOMES_SIG003;MEL_AUTOSOMES_SIG017	ld_interval_overlap
ENSGALG00010005579	ENSGALG00010005579	1	4_50960354	variant_gene_overlap	0		
ENSGALG00010004924	TMPRSS11F	1	4_51236931	variant_gene_overlap	0		
ENSGALG00010005582	ENSGALG00010005582	0			1	MEC_AUTOSOMES_SIG004	nearest_within_500kb
ENSGALG00010004095	ANKRD50	1	4_52426323	variant_gene_overlap	1	MEC_AUTOSOMES_SIG017	ld_interval_overlap
ENSGALG00010005750	ENSGALG00010005750	1	4_52438311;4_52438385;4_52438421;4_52438433;4_52439598;4_52451790;4_52464126;4_52467216;4_52474334;4_52474918;4_52475158;4_52476344;4_52479037;4_52481561;4_52482069;4_52482110;4_52488692;4_52489714;4_52490844	variant_gene_overlap;variant_nearest_within_5kb	1	MEC_AUTOSOMES_SIG002;MEC_AUTOSOMES_SIG013	nearest_within_500kb
ENSGALG00010005603	ENSGALG00010005603	1	4_52474334;4_52474918;4_52475158;4_52476344;4_52479037	variant_gene_overlap	0		
ENSGALG00010005606	ENSGALG00010005606	1	4_52476344	variant_gene_overlap	0		
ENSGALG00010005766	ENSGALG00010005766	1	4_52663746	variant_gene_overlap	0		
ENSGALG00010003978	ANK2	1	4_56500442	variant_gene_overlap	1	MEC_AUTOSOMES_SIG012	ld_interval_overlap
ENSGALG00010004001	LARP7	1	4_56605141	variant_gene_overlap	0		
ENSGALG00010004366	UNC5C	1	4_58233741	variant_gene_overlap	1	MEL_AUTOSOMES_SIG026	ld_interval_overlap
ENSGALG00010005897	ENSGALG00010005897	0			1	MEL_AUTOSOMES_SIG015	nearest_within_500kb
ENSGALG00010005904	ENSGALG00010005904	1	4_58890405	variant_gene_overlap	0		
ENSGALG00010005914	ENSGALG00010005914	1	4_58890405;4_59094671;4_59165705;4_59176477;4_59178040	variant_gene_overlap	1	MEC_AUTOSOMES_SIG007	ld_interval_overlap

GENE ID	GENE NAME	STRICT VARIANT EVIDENCE	STRICT VARIANTS	STRICT MAPPING MODE	REGION GENE EVIDENCE	REGION SIGNALS	REGION MAPPING MODE
ENSGALG00010004716	ADH6	1	4_59483846	variant_gene_overlap	0		
ENSGALG00010005241	C4H4orf54	1	4_59544379;4_59544384	variant_gene_overlap	1	MEL_AUTOSOMES_SIG021	ld_interval_overlap
ENSGALG00010005268	MTTP	1	4_59544379;4_59544384	variant_gene_overlap	1	MEL_AUTOSOMES_SIG021	ld_interval_overlap
ENSGALG00010023599	ENSGALG00010023599	1	5_20885150	variant_gene_overlap	1	MEC_AUTOSOMES_SIG009	ld_interval_overlap
ENSGALG00010023610	ENSGALG00010023610	1	5_20885150	variant_gene_overlap	1	MEC_AUTOSOMES_SIG009	ld_interval_overlap
ENSGALG00010021257	RGS6	1	5_26207271	variant_gene_overlap	1	MEC_AUTOSOMES_SIG014	ld_interval_overlap
ENSGALG00010015759	EIF5	1	5_49728859	variant_gene_overlap	1	MEC_AUTOSOMES_SIG011	ld_interval_overlap
ENSGALG00010018032	ERO1A	1	5_57964781	variant_gene_overlap	1	MEL_AUTOSOMES_SIG016	ld_interval_overlap
ENSGALG00010011180	ENSGALG00010011180	0			1	MEL_AUTOSOMES_SIG008	nearest_within_500kb
ENSGALG00010010438	LDB3	1	6_3146546;6_3146578;6_3172247;6_3172268	variant_gene_overlap	1	MEC_AUTOSOMES_SIG003;MEC_AUTOSOMES_SIG005	ld_interval_overlap
ENSGALG00010024404	ENSGALG00010024404	1	8_15513773	variant_gene_overlap	1	MEL_AUTOSOMES_SIG011	ld_interval_overlap
ENSGALG00010023030	HS2ST1	1	8_15579761	variant_gene_overlap	0		
ENSGALG00010023059	SELENOF	1	8_15638386	variant_gene_overlap	0		
ENSGALG00010023101	CLCA2	1	8_15690023	variant_gene_overlap	0		
ENSGALG00010023161	ODF2L	1	8_15718134	variant_nearest_within_5kb	0		
ENSGALG00010023195	COL24A1	1	8_15795166	variant_gene_overlap	1	MEL_AUTOSOMES_SIG009	ld_interval_overlap
ENSGALG00010016761	GPR160	1	9_19567769	variant_gene_overlap	1	MEC_AUTOSOMES_SIG019	ld_interval_overlap
ENSGALG00010001267	BCHE	1	9_20591655	variant_gene_overlap	1	MEL_AUTOSOMES_SIG010	ld_interval_overlap
ENSGALG00010001625	ENSGALG00010001625	1	9_20921150;9_20931265	variant_gene_overlap;variant_nearest_within_5kb	1	MEC_AUTOSOMES_SIG015	nearest_within_500kb
ENSGALG00010001631	ENSGALG00010001631	1	9_20931265;9_20976534	variant_gene_overlap	1	MEC_AUTOSOMES_SIG018;MEL_AUTOSOMES_SIG014	ld_interval_overlap;nearest_within_500kb
ENSGALG00010001629	ENSGALG00010001629	1	9_20976534	variant_gene_overlap	1	MEC_AUTOSOMES_SIG018	ld_interval_overlap
ENSGALG00010024121	NRG4	1	10_3508373	variant_gene_overlap	1	MEL_AUTOSOMES_SIG023	ld_interval_overlap
ENSGALG00010016558	NR2F2	0			1	MEL_AUTOSOMES_SIG006	nearest_within_500kb
ENSGALG00010023405	DNAJA2	1	11_7373893;11_7373963;11_7374024	variant_gene_overlap	1	MEC_AUTOSOMES_SIG001	ld_interval_overlap
ENSGALG00010023513	CDCA9	1	11_7373893;11_7373963;11_7374024	variant_gene_overlap	1	MEC_AUTOSOMES_SIG001	ld_interval_overlap
ENSGALG00010027399	MAPKAP1	1	17_10307375;17_10318885	variant_gene_overlap	0		
ENSGALG00010027414	PBX3	1	17_10368305;17_10404459;17_10404889;17_10405077	variant_gene_overlap	1	MEL_AUTOSOMES_SIG004	ld_interval_overlap
ENSGALG00010029363	PIK3R6	1	18_2601049;18_2602079;18_2602114	variant_gene_overlap	1	MEL_AUTOSOMES_SIG007	ld_interval_overlap
ENSGALG00010028815	ENSGALG00010028815	0			1	MEL_AUTOSOMES_SIG019	nearest_within_500kb
ENSGALG00010028283	CERS4	1	28_521922;28_521968	variant_gene_overlap	1	MEC_AUTOSOMES_SIG010	ld_interval_overlap
ENSGALG00010010985	COL4A3BP	1	chrZ_24478892	variant_gene_overlap	1	MEL_CHRZ_SIG001	ld_interval_overlap

Table S2. Part A1. Six genomic prediction models: predictive ability, scaled accuracy, and RMSE.

Trait	Model	Repeats (n)	Predictive ability mean	Predictive ability SD	Scaled accuracy mean	Scaled accuracy SD	RMSE mean	RMSE SD
MeL	GBLUP	10	0.112924	0.0255878	0.218979	0.0496193	4.08436	0.0299818
MeL	BayesA	10	0.11837	0.0187759	0.22954	0.0364098	4.08603	0.0300075
MeL	BayesB	10	0.116464	0.0181795	0.225844	0.0352533	4.08647	0.0301222
MeL	BayesCpi	10	0.111556	0.0201485	0.216327	0.0390714	4.08595	0.0284759
MeL	Bayesian LASSO	10	0.101691	0.0354384	0.197198	0.0687213	4.08805	0.0298811
MeL	RKHS	10	0.106404	0.0442314	0.206336	0.0857725	4.09267	0.0263116
MeC	GBLUP	10	0.231514	0.0310255	0.322227	0.0431822	0.391453	0.00176232
MeC	BayesA	10	0.226877	0.0309721	0.315774	0.0431077	0.391843	0.00175953
MeC	BayesB	10	0.221186	0.036372	0.307853	0.0506235	0.39163	0.00210954
MeC	BayesCpi	10	0.230151	0.0327282	0.320331	0.0455519	0.391686	0.00186741
MeC	Bayesian LASSO	10	0.204068	0.0329377	0.284027	0.0458436	0.394711	0.0014938
MeC	RKHS	10	0.19614	0.0531416	0.272993	0.0739638	0.394581	0.00128784

Table S2 (continued). Part A2. Six genomic prediction models: MAE, mean bias, and calibration slope.

Trait	Model	MAE mean	MAE SD	Mean bias mean	Mean bias SD	Regression slope mean	Regression slope SD
MeL	GBLUP	3.15185	0.0282114	-0.0222108	0.0201597	1.02759	0.247144
MeL	BayesA	3.1525	0.0279218	-0.0163286	0.0202157	0.942521	0.204638
MeL	BayesB	3.15371	0.0310654	-0.0258323	0.0247783	0.903672	0.176019
MeL	BayesCpi	3.15492	0.0277106	-0.0204596	0.0221121	1.05122	0.215882
MeL	Bayesian LASSO	3.15647	0.0297818	-0.00874898	0.0187564	1.13461	0.386711
MeL	RKHS	3.16192	0.0233473	-0.0140495	0.0132408	1.16564	0.437636
MeC	GBLUP	0.293879	0.00110541	-0.000443614	0.00290612	1.64855	0.256412
MeC	BayesA	0.294134	0.000831279	-0.000495784	0.00281855	1.66424	0.274666
MeC	BayesB	0.294008	0.00138304	-0.000744273	0.00308901	1.548	0.261003
MeC	BayesCpi	0.294175	0.000953756	-0.000381258	0.0026162	1.67892	0.26467
MeC	Bayesian LASSO	0.297174	0.000962543	-0.000592161	0.0020888	2.34545	0.593116
MeC	RKHS	0.296801	0.000502568	-0.000994957	0.00171594	1.79207	0.608795

Table S2 (continued). Part B1. Marker-set comparisons: predictive ability, scaled accuracy, and RMSE.

Trait	Model	Repeats (n)	Predictive ability mean	Predictive ability SD	Scaled accuracy mean	Scaled accuracy SD	RMSE mean	RMSE SD
MeL	ALL	10	0.0593158	0.0327675	0.115024	0.063542	4.12078	0.0279121
MeL	CANDIDATE	10	0.35661	0.0148511	0.69153	0.0287989	3.88149	0.0297994
MeL	GFBLUP	10	0.343983	0.0228478	0.667045	0.0443059	3.91057	0.0328946
MeL	RANDOM_MEAN	10	0.0441411	0.0372621	0.0855974	0.0722579	4.13477	0.0288612

Trait	Model	Repeats (n)	Predictive ability mean	Predictive ability SD	Scaled accuracy mean	Scaled accuracy SD	RMSE mean	RMSE SD
MeC	ALL	10	0.134301	0.0359243	0.186923	0.0500004	0.397042	0.00241822
MeC	CANDIDATE	10	0.37858	0.0300417	0.526918	0.0418128	0.370787	0.00399832
MeC	GFBLUP	10	0.356584	0.034875	0.496303	0.0485399	0.373962	0.00388442
MeC	RANDOM_MEAN	10	0.0785539	0.0406143	0.109333	0.0565281	0.399862	0.00219671

Table S2 (continued). Part B2. Marker-set comparisons: MAE, mean bias, and calibration slope.

Trait	Model	MAE mean	MAE SD	Mean bias mean	Mean bias SD	Regression slope mean	Regression slope SD
MeL	ALL	3.17694	0.0266589	-0.0275382	0.0331534	0.448164	0.227149
MeL	CANDIDATE	3.06665	0.0299632	-0.0216587	0.0581577	1.0472	0.0568991
MeL	GFBLUP	3.07325	0.0321687	-0.0343927	0.0641019	1.03145	0.0616874
MeL	RANDOM_MEAN	3.19079	0.0275113	-0.0299372	0.0296097	0.323519	0.283106
MeC	ALL	0.29881	0.00164057	-0.000876993	0.00322058	0.902989	0.26061
MeC	CANDIDATE	0.283175	0.00254977	-0.0051392	0.00370443	1.16198	0.0869965
MeC	GFBLUP	0.285035	0.00244907	-0.00499205	0.00397802	1.19363	0.12905
MeC	RANDOM_MEAN	0.301039	0.00166883	-0.0015098	0.00340911	0.537957	0.281741

Table S2 (continued). Part C. Paired comparisons of prediction accuracy.

Part	Trait	Comparison	Mean difference	SD of difference	Paired t-test P	Exact Wilcoxon P
Part1	MeL	BAYESA - GBLUP	0.0105612	0.0261946	0.234248	0.322266
Part1	MeC	BAYESA - GBLUP	-0.00645334	0.0144783	0.192292	0.232422
Part1	MeL	BAYESB - GBLUP	0.00686495	0.0376404	0.578251	1
Part1	MeC	BAYESB - GBLUP	-0.014374	0.0295784	0.158732	0.160156
Part1	MeL	BAYESCPI - GBLUP	-0.0026514	0.0373086	0.827207	1
Part1	MeC	BAYESCPI - GBLUP	-0.00189626	0.0151037	0.700603	0.695312
Part1	MeL	BL - GBLUP	-0.0217811	0.0336628	0.0710594	0.105469
Part1	MeC	BL - GBLUP	-0.0382004	0.0198444	0.000182014	0.00195312
Part1	MeL	RKHS - GBLUP	-0.0126423	0.0892124	0.664654	0.556641
Part1	MeC	RKHS - GBLUP	-0.0492337	0.083638	0.0955878	0.0644531
Part2	MeL	CANDIDATE - ALL	0.576507	0.0552388	1.05977e-10	0.00195312
Part2	MeC	CANDIDATE - ALL	0.339995	0.0268836	1.90127e-11	0.00195312
Part2	MeL	GFBLUP - ALL	0.552021	0.0432463	1.74996e-11	0.00195312
Part2	MeC	GFBLUP - ALL	0.309379	0.0203154	3.59753e-12	0.00195312
Part2	MeL	CANDIDATE - RANDOM_MEAN	0.605933	0.0513638	3.54415e-11	0.00195312
Part2	MeC	CANDIDATE - RANDOM_MEAN	0.417585	0.0393752	9.18359e-11	0.00195312

Part	Trait	Comparison	Mean difference	SD of difference	Paired t-test P	Exact Wilcoxon P
Part2	MeL	GFBLUP - RANDOM_MEAN	0.581447	0.0426314	9.66651e-12	0.00195312
Part2	MeC	GFBLUP - RANDOM_MEAN	0.386969	0.0381588	1.36978e-10	0.00195312
Part2	MeL	CANDIDATE - GFBLUP	0.0244858	0.0287965	0.0248414	0.0273438
Part2	MeC	CANDIDATE - GFBLUP	0.0306154	0.0165417	0.000242921	0.00195312

Table S2 (continued). Part D. Paired tests for the six-model comparison.

TRAIT	MODEL	Mean difference	SD of difference	Paired t-test P	Wilcoxon P
MeC	BAYESA	-0.00645334	0.0144783	0.192292	0.221272
MeC	BAYESB	-0.014374	0.0295784	0.158732	0.153576
MeC	BAYESCPI	-0.00189626	0.0151037	0.700603	0.683481
MeC	BL	-0.0382004	0.0198444	0.000182014	0.00592154
MeC	RKHS	-0.0492337	0.083638	0.0955878	0.0665457
MeL	BAYESA	0.0105612	0.0261946	0.234248	0.308063
MeL	BAYESB	0.00686495	0.0376404	0.578251	1
MeL	BAYESCPI	-0.0026514	0.0373086	0.827207	1
MeL	BL	-0.0217811	0.0336628	0.0710594	0.102918
MeL	RKHS	-0.0126423	0.0892124	0.664654	0.540818

Table S2 (continued). Part E. Paired tests for the marker-set comparison.

TRAIT	MODEL	Mean difference	SD of difference	Paired t-test P	Wilcoxon P
MeC	CANDIDATE	0.339995	0.0268836	1.90127e-11	0.00592154
MeC	GFBLUP	0.309379	0.0203154	3.59753e-12	0.00592154
MeC	RANDOM1	-0.0705977	0.0469298	0.00103387	0.00804524
MeC	RANDOM2	-0.0794162	0.0258354	4.52769e-06	0.00592154
MeC	RANDOM3	-0.075876	0.0295997	1.99181e-05	0.00592154
MeC	RANDOM4	-0.0770453	0.0457215	0.000475463	0.00592154
MeC	RANDOM5	-0.0850144	0.0222811	7.34939e-07	0.00592154
MeL	CANDIDATE	0.576507	0.0552388	1.05977e-10	0.00592154
MeL	GFBLUP	0.552021	0.0432463	1.74996e-11	0.00592154
MeL	RANDOM1	-0.0366137	0.0675352	0.120598	0.102918
MeL	RANDOM2	-0.0408196	0.067211	0.0869777	0.153576
MeL	RANDOM3	-0.0257825	0.0647735	0.239801	0.185144
MeL	RANDOM4	-0.0119891	0.067773	0.589532	0.475533
MeL	RANDOM5	-0.0319267	0.0636509	0.147162	0.262193

Table S3. Bioinformatics and statistical software used in this study

Software	Version	Main application in this study	Website/URL
PLINK	v1.90b6.21	Genotype quality control, LD pruning, and linkage disequilibrium analysis	https://www.cog-genomics.org/plink/1.9/
GEMMA	v0.98.5	Genomic heritability estimation, single-trait GWAS, and bivariate GWAS	https://github.com/genetics-statistics/GEMMA
VCFtools	v0.1.16	Genetic differentiation (F_ST) and nucleotide diversity (π) analyses	https://vcftools.github.io/
Matplotlib	V3.11	Generation of Manhattan and Q–Q plots	https://matplotlib.org/
SnpEff	v4.3.1t	Functional annotation and prediction of variant effects	https://pcingola.github.io/SnpEff/
R	v4.2.3	Statistical analyses and implementation of genomic prediction models	https://www.r-project.org/
BGLR	v1.1.2	Implementation of BayesA, BayesB, BayesCπ, Bayesian LASSO, and RKHS models	https://search.r-project.org/CRAN/refmans/BGLR/html/00Index.html
HIBLUP	v1.6.0	Construction of additive genomic relationship matrices	https://www.hiblup.com/
ASReml-R	v4.2	Fitting GBLUP and GFBLUP models	https://vsni.co.uk/software/asreml-r/

Table S4. Retention of prioritized SNPs across alternative cutoffs

Trait	Scope	10% priority SNPs	Retained at 15%	Retained at 20%	Retained at both	Retained at either
MeL	Autosomes	55	37/55 (67.27%)	36/55 (65.45%)	34/55 (61.82%)	39/55 (70.91%)
MeL	Chr Z	1	1/1 (100.00%)	1/1 (100.00%)	1/1 (100.00%)	1/1 (100.00%)
MeC	Autosomes	62	48/62 (77.42%)	47/62 (75.81%)	42/62 (67.74%)	53/62 (85.48%)
MeC	Chr Z	0	NA	NA	NA	NA

Note: The primary analysis used the upper and lower 10% of the within-line, body-weight-adjusted phenotype distributions. Each extreme group contained 33 birds at the 10% cutoff (16 HAF and 17 LAF), 50 at 15% (24 HAF and 26 LAF), and 66 at 20% (32 HAF and 34 LAF). A prioritized SNP was considered retained if its position remained within a candidate region with the same phenotypic direction as in the primary analysis. Percentages use the number of prioritized SNPs in the primary analysis as the denominator. "Both" denotes retention at both alternative cutoffs, and "either" denotes retention at one or both cutoffs. Chromosome Z results are reported separately from the primary autosomal analysis. NA indicates that no prioritized SNP was present in that trait–chromosome category in the primary analysis. HAF and LAF denote the high- and low-abdominal-fat lines, respectively.
